# Supplementary material for: Djakonoviosides A, A1, A2, B1–B4 — Triterpene Monosulfated Tetra- and Pentaosides from the Sea Cucumber Cucumaria djakonovi: The First Finding of a Hemiketal Fragment in the Aglycones; Activity against Human Breast Cancer Cell Lines
Source: Int J Mol Sci. 2023 Jul 5;24(13):11128. doi: 10.3390/ijms241311128 (PMC10342010; doi:10.3390/ijms241311128)
Supplement: Supplementary file 1 [file ijms-24-11128-s001.zip › ijms-2493011-supplementary.pdf]

Supplementary data content page

Djakonoviosides A, A_1, A_2, B_1–B_4 — Triterpene Monosulfated Tetra- and Pentaosides from the Sea Cucumber *Cucumaria djakonovi*: The First Finding of a Hemiketal Fragment in the Aglycones; Activity against Human Breast Cancer Cell Lines

Alexandra S. Silchenko [1,*], Anatoly I. Kalinovsky [1], Sergey A. Avilov [1], Roman S. Popov [1], Pavel S. Dmitrenok [1], Ekaterina A. Chingizova [1], Ekaterina S. Menchinskaya [1], Elena G. Panina [2], Vadim G. Stepanov [2], Vladimir I. Kalinin [1,*] and Valentin A. Stonik [1]

[1] G.B. Elyakov Pacific Institute of Bioorganic Chemistry, Far Eastern Branch of the Russian Academy of Sciences, Pr. 100-letya Vladivostoka 159, 690022 Vladivostok, Russia; kaaniv@piboc.dvo.ru (A.I.K.); avilov_sa@piboc.dvo.ru (S.A.A.); popov_rs@piboc.dvo.ru (R.S.P.); paveldmt@piboc.dvo.ru (P.S.D.); chingizova_ea@piboc.dvo.ru (E.A.C.); ekaterinamenchinskaya@gmail.com (E.S.M.); stonik@piboc.dvo.ru (V.A.S.)

[2] Kamchatka Branch of Pacific Institute of Geography, Far Eastern Branch of the Russian Academy of Sciences, Partizanskaya st. 6, 683000 Petropavlovsk-Kamchatsky, Russia; egpanina777@gmail.com (E.G.P.); stepanovvadim24@gmail.com (V.G.S.)

* Correspondence: silchenko_als@piboc.dvo.ru (A.S.S.); kalininv@piboc.dvo.ru (V.I.K.); Tel./Fax: +7-(423)2-31-40-50 (A.S.S. & V.I.K.)

Content:

Figure S1. The ^{13}C NMR (125.67 MHz) spectrum of djakonovioside A (**1**) in C_5D_5N

Figure S2. The ^{1}H NMR (500.12 MHz) spectrum of djakonovioside A (**1**) in C_5D_5N

Figure S3. The COSY (500.12 MHz) spectrum of djakonovioside A (**1**) in C_5D_5N

Figure S4. The HSQC (500.12 MHz) spectrum of djakonovioside A (**1**) in C_5D_5N

Figure S5. The ROESY (500.12 MHz) spectrum of djakonovioside A (**1**) in C_5D_5N

Figure S6. The HMBC (500.12 MHz) spectrum of djakonovioside A (**1**) in C_5D_5N

Figure S7. 1 D TOCSY (500.12 MHz) spectra of Xyl1, Qui2, Xyl3, MeGlc4 of djakonovioside A (**1**) in C_5D_5N

Figure S8. HR-ESI-MS and ESI-MS/MS spectra of djakonovioside A (**1**)

Figure S9. Δ^{SR} sign distribution in 23-O-MTPA esters of **1** and absolute configuration of C-23 stereocenter.

Figure S10. The ^{13}C NMR (125.67 MHz) spectrum of djakonovioside A_1 (**2**) in C_5D_5N/D_2O (4/1)

Figure S11. The ^{1}H NMR (500.12 MHz) spectrum of djakonovioside A_1 (**2**) in C_5D_5N/D_2O (4/1)

Figure S12. The COSY (500.12 MHz) spectrum of djakonovioside A_1 (**2**) in C_5D_5N/D_2O (4/1)

Figure S13. The HSQC (500.12 MHz) spectrum of djakonovioside A_1 (**2**) in C_5D_5N/D_2O (4/1)

Figure S14. The HMBC (500.12 MHz) spectrum of djakonovioside A_1 (**2**) in C_5D_5N/D_2O (4/1)

Figure S15. The ROESY (500.12 MHz) spectrum of djakonovioside A$_1$ (**2**) in C_5D_5N/D_2O (4/1)

Figure S16. 1D TOCSY (500.12 MHz) spectra of Xyl1, Qui2, Xyl3, MeGlc4 of djakonovioside A$_1$ (**2**) in C_5D_5N/D_2O (4/1)

Figure S17. HR-ESI-MS and ESI-MS/MS spectra of djakonovioside A$_1$ (**2**)

Table S1. ^{13}C and ^1H NMR chemical shifts, HMBC and ROESY correlations of the carbohydrate part of djakonovioside A$_1$ (**2**)

Figure S18. The ^{13}C NMR (125.67 MHz) spectrum of djakonovioside A$_2$ (**3**) in C_5D_5N/D_2O (4/1)

Figure S19. The ^1H NMR (500.12 MHz) spectrum of djakonovioside A$_2$ (**3**) in C_5D_5N/D_2O (4/1)

Figure S20. The COSY (500.12 MHz) spectrum of djakonovioside A$_2$ (**3**) in C_5D_5N/D_2O (4/1)

Figure S21. The HSQC (500.12 MHz) spectrum of djakonovioside A$_2$ (**3**) in C_5D_5N/D_2O (4/1)

Figure S22. The ROESY (500.12 MHz) spectrum of djakonovioside A$_2$ (**3**) in C_5D_5N/D_2O (4/1)

Figure S23. The HMBC (500.12 MHz) spectrum of djakonovioside A$_2$ (**3**) in C_5D_5N/D_2O (4/1)

Figure S24. 1D TOCSY (500.12 MHz) spectra of Xyl1, Qui2, Xyl3, MeGlc4 of djakonovioside A$_2$ (**3**) in C_5D_5N/D_2O (4/1)

Figure S25. HR-ESI-MS and ESI-MS/MS spectra of djakonovioside A$_2$ (**3**)

Table S2. ^{13}C and ^1H NMR chemical shifts, HMBC and ROESY correlations of the carbohydrate part of djakonovioside A$_2$ (**3**)

Figure S26. The ^{13}C NMR (125.67 MHz) spectrum of djakonovioside B$_1$ (**4**) in C_5D_5N/D_2O (4/1)

Figure S27. The ^1H NMR (500.12 MHz) spectrum of djakonovioside B$_1$ (**4**) in C_5D_5N/D_2O (4/1)

Figure S28. The COSY (500.12 MHz) spectrum of djakonovioside B$_1$ (**4**) in C_5D_5N/D_2O (4/1)

Figure S29. The HSQC (500.12 MHz) spectrum of djakonovioside B$_1$ (**4**) in C_5D_5N/D_2O (4/1)

Figure S30. The ROESY (500.12 MHz) spectrum of djakonovioside B$_1$ (**4**) in C_5D_5N/D_2O (4/1)

Figure S31. The HMBC (500.12 MHz) spectrum of djakonovioside B$_1$ (**4**) in C_5D_5N/D_2O (4/1)

Figure S32. 1D TOCSY (500.12 MHz) spectra of Xyl1, Qui2, Xyl3, MeGlc4, Xyl5 of djakonovioside B$_1$ (**4**) in C_5D_5N/D_2O (4/1)

Figure S33. HR-ESI-MS and ESI-MS/MS spectra of djakonovioside B$_1$ (**4**)

Table S3. ^{13}C and ^1H NMR chemical shifts, HMBC and ROESY correlations of the aglycone part of djakonovioside B$_1$ (**4**)

Figure S34. The ^{13}C NMR (125.67 MHz) spectrum of djakonovioside B$_2$ (**5**) in C_5D_5N/D_2O (4/1)

Figure S35. The ^1H NMR (500.12 MHz) spectrum of djakonovioside B$_2$ (**5**) in C_5D_5N/D_2O (4/1)

Figure S36. The COSY (500.12 MHz) spectrum of djakonovioside B$_2$ (**5**) in C_5D_5N/D_2O (4/1)

Figure S37. The HSQC (500.12 MHz) spectrum of djakonovioside B$_2$ (**5**) in C_5D_5N/D_2O (4/1)

Figure S38. The ROESY (500.12 MHz) spectrum of djakonovioside B$_2$ (**5**) in C_5D_5N/D_2O (4/1)

Figure S39. The HMBC (500.12 MHz) spectrum of djakonovioside B$_2$ (**5**) in C_5D_5N/D_2O (4/1)

Figure S40. 1D TOCSY (500.12 MHz) spectra of Xyl1, Qui2, Xyl3, MeGlc4, Xyl5 of djakonovioside B$_2$ (**5**) in C_5D_5N/D_2O (4/1)

Figure S41. HR-ESI-MS and ESI-MS/MS spectra of djakonovioside B$_2$ (**5**)

Table S4. ^{13}C and ^{1}H NMR chemical shifts, HMBC and ROESY correlations of the carbohydrate part of djakonovioside B$_2$ (**5**)

Figure S42. The ^{13}C NMR (125.67 MHz) spectrum of djakonovioside B$_3$ (**6**) in C_5D_5N/D_2O (4/1)

Figure S43. The ^{1}H NMR (500.12 MHz) spectrum of djakonovioside B$_3$ (**6**) in C_5D_5N/D_2O (4/1)

Figure S44. The COSY (500.12 MHz) spectrum of djakonovioside B$_3$ (**6**) in C_5D_5N/D_2O (4/1)

Figure S45. The HSQC (500.12 MHz) spectrum of djakonovioside B$_3$ (**6**) in C_5D_5N/D_2O (4/1)

Figure S46. The ROESY (500.12 MHz) spectrum of djakonovioside B$_3$ (**6**) in C_5D_5N/D_2O (4/1)

Figure S47. The HMBC (500.12 MHz) spectrum of djakonovioside B$_3$ (**6**) in C_5D_5N/D_2O (4/1)

Figure S48. 1D TOCSY (500.12 MHz) spectra of Xyl1, Qui2, Xyl3, MeGlc4, Xyl5 of djakonovioside B$_3$ (**6**) in C_5D_5N/D_2O (4/1)

Figure S49. HR-ESI-MS and ESI-MS/MS spectra of djakonovioside B$_3$ (**6**)

Table S5. ^{13}C and ^{1}H NMR chemical shifts, HMBC and ROESY correlations of the carbohydrate part of djakonovioside B$_3$ (**6**)

Figure S50. The ^{13}C NMR (125.67 MHz) spectrum of djakonovioside B$_4$ (**7**) in C_5D_5N/D_2O (4/1)

Figure S51. The ^{1}H NMR (500.12 MHz) spectrum of djakonovioside B$_4$ (**7**) in C_5D_5N/D_2O (4/1)

Figure S52. The COSY (500.12 MHz) spectrum of djakonovioside B$_4$ (**7**) in C_5D_5N/D_2O (4/1)

Figure S53. The HSQC (500.12 MHz) spectrum of djakonovioside B$_4$ (**7**) in C_5D_5N/D_2O (4/1)

Figure S54. The ROESY (500.12 MHz) spectrum of djakonovioside B$_4$ (**7**) in C_5D_5N/D_2O (4/1)

Figure S55. The HMBC (500.12 MHz) spectrum of djakonovioside B$_4$ (**7**) in C_5D_5N/D_2O (4/1)

Figure S56. 1D TOCSY (500.12 MHz) spectra of Xyl1, Qui2, Xyl3, MeGlc4, Xyl5 of djakonovioside B$_4$ (**7**) in C_5D_5N/D_2O (4/1)

Figure S57. HR-ESI-MS and ESI-MS/MS spectra of djakonovioside B$_4$ (**7**)

Table S6. ^{13}C and ^{1}H NMR chemical shifts, HMBC and ROESY correlations of the carbohydrate part of djakonovioside B$_4$ (**7**)

Table S7. ^{13}C and ^{1}H NMR chemical shifts, HMBC and ROESY correlations of the aglycone part of djakonovioside B$_4$ (**7**)

Figure S58. The ^{13}C NMR (125.67 MHz) spectrum of acetylated derivative **7a** in C_5D_5N

Figure S59. The ^{1}H NMR (500.12 MHz) spectrum of acetylated derivative **7a** in C_5D_5N

Figure S60. The COSY (500.12 MHz) spectrum of acetylated derivative **7a** in C_5D_5N

Figure S61. The HSQC (500.12 MHz) spectrum of acetylated derivative **7a** in C_5D_5N

Figure S62. The ROESY (500.12 MHz) spectrum of acetylated derivative **7a** in C_5D_5N

Figure S63. The HMBC (500.12 MHz) spectrum of acetylated derivative **7a** in C_5D_5N

Figure S64. HR-ESI-MS spectrum of acetylated derivatives of djakonovioside B_4 (**7**)

Table S8. ^{13}C and ^{1}H NMR chemical shifts, HMBC and ROESY correlations of the aglycone part of acetylated derivative **7a**

Figure S65. Formulae of known glycosides isolated from *C. djakonovi*

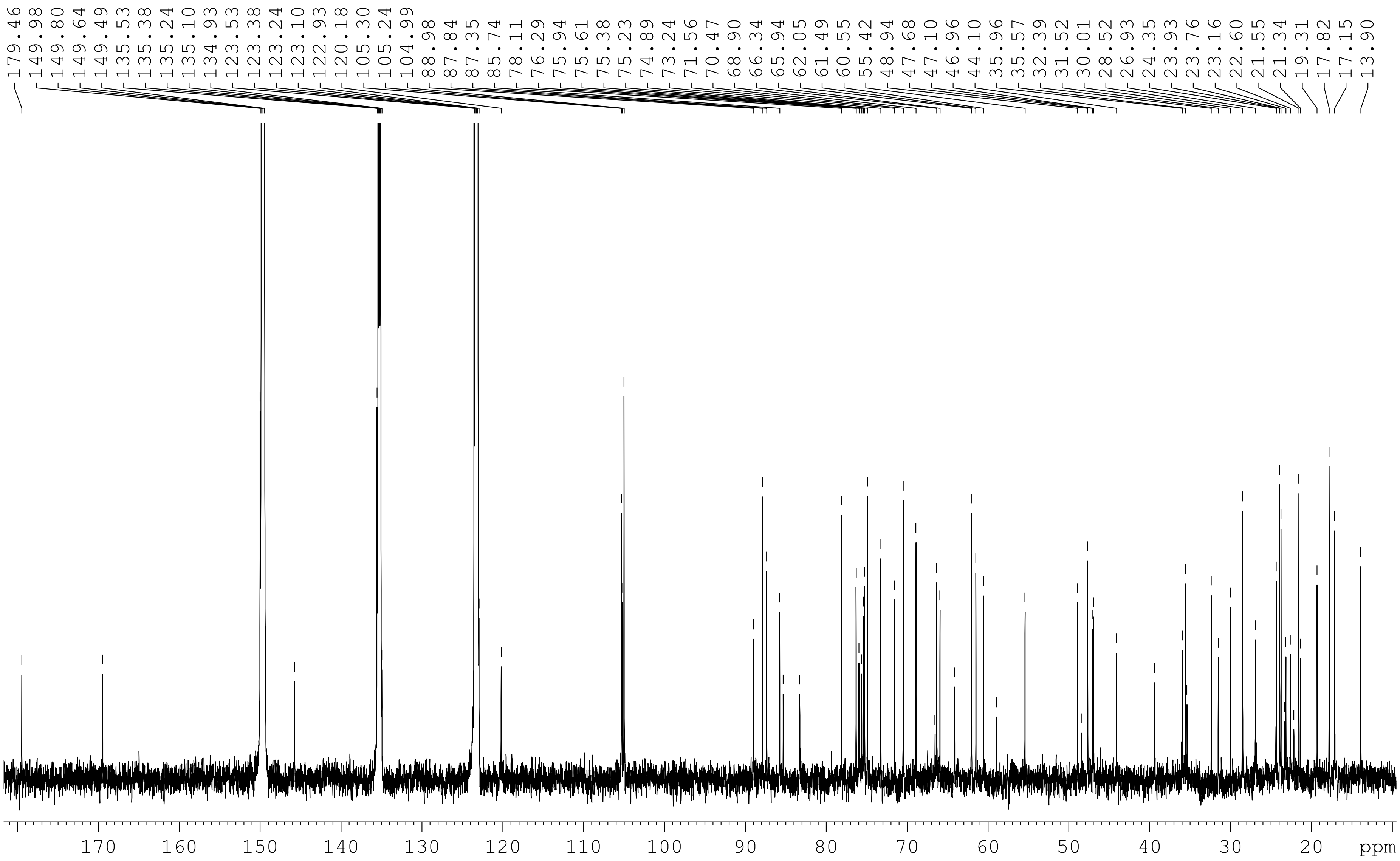

Figure S1. The ^{13}C NMR (125.67 MHz) spectrum of djakonovioside A (**1**) in C_5D_5N

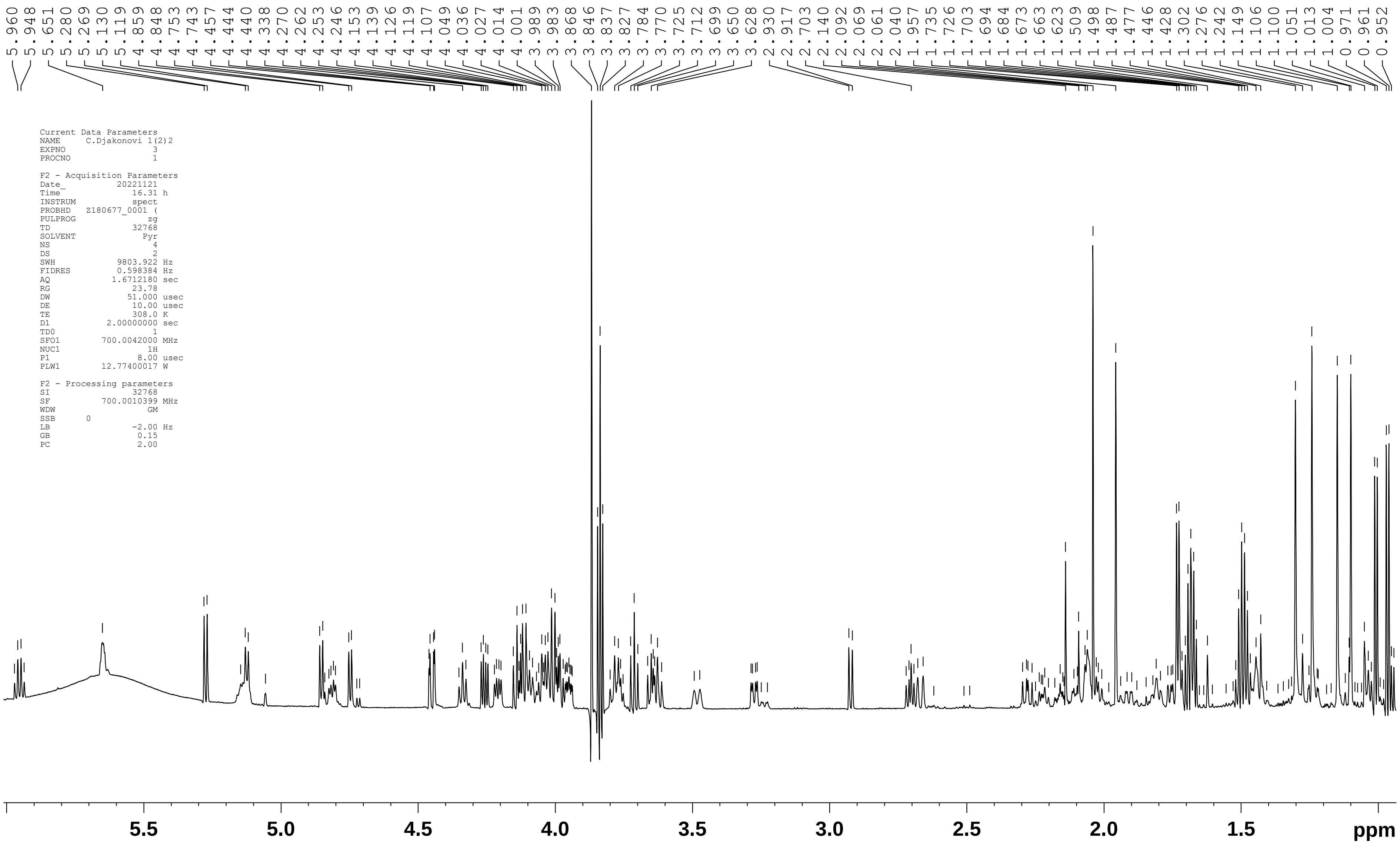

Figure S2. The ^{1}H NMR (500.12 MHz) spectrum of djakonovioside A (**1**) in C_5D_5N

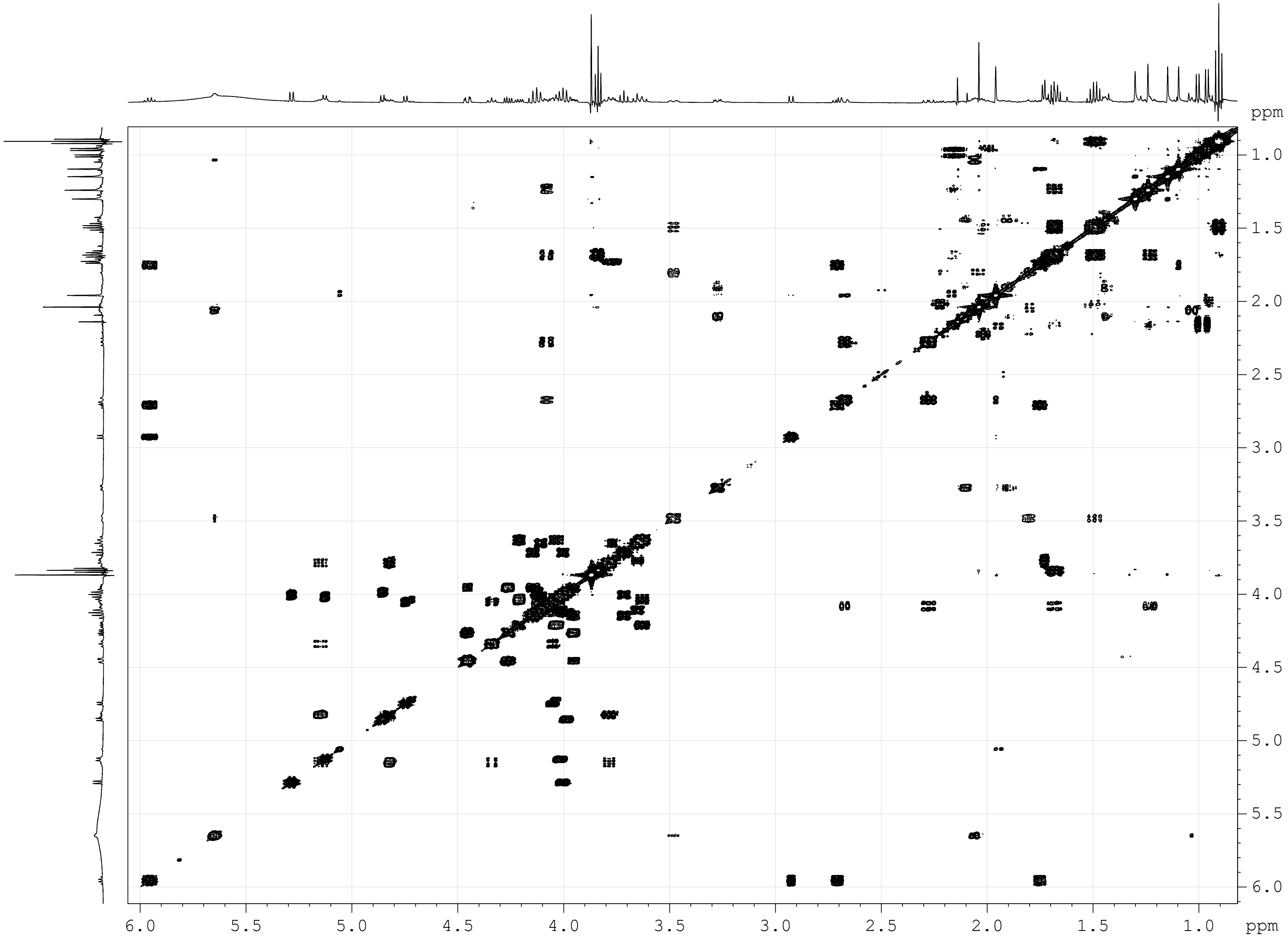

Figure S3. The COSY (500.12 MHz) spectrum of djakonovioside A (**1**) in C_5D_5N

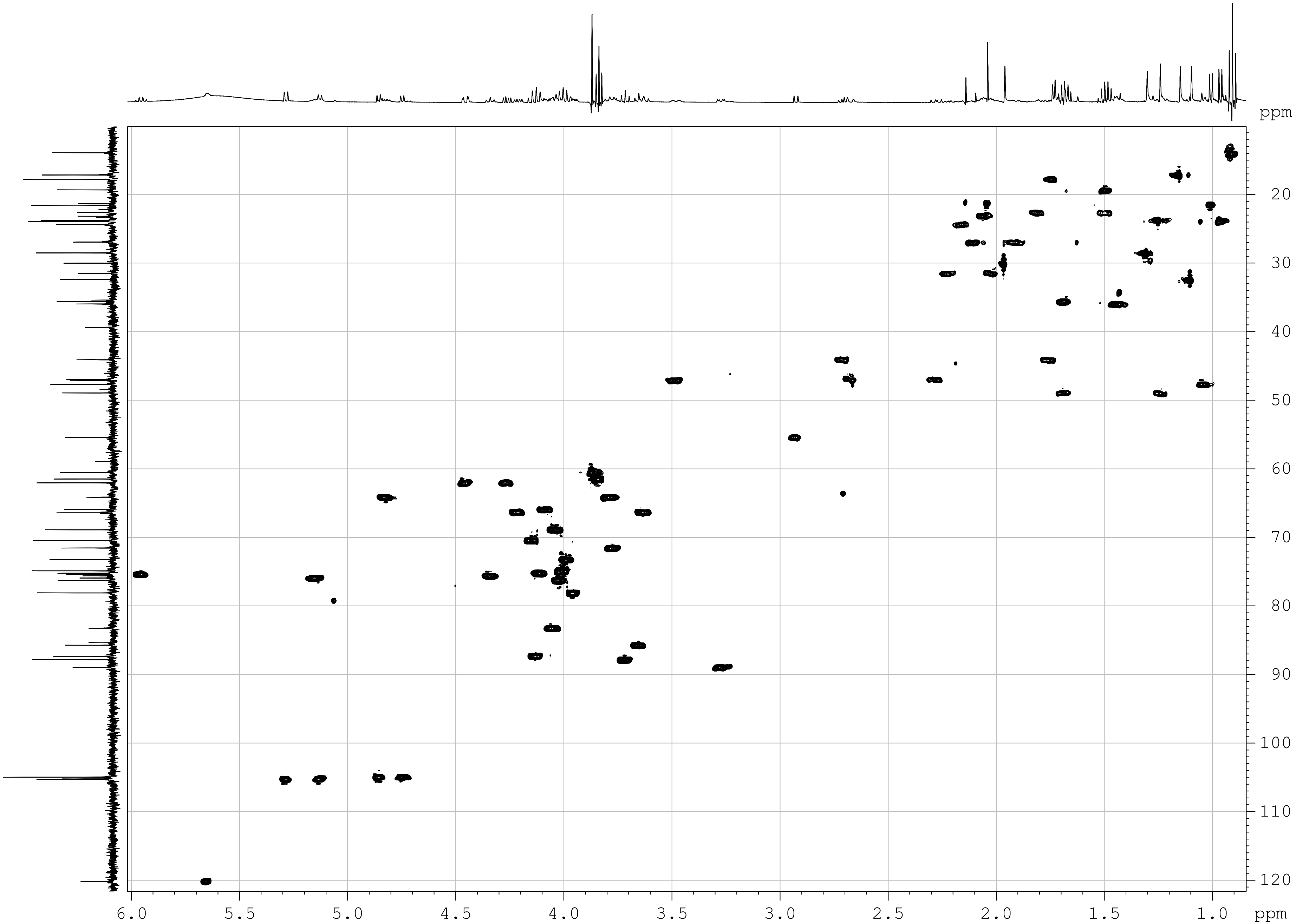

Figure S4. The HSQC (500.12 MHz) spectrum of djakonovioside A (**1**) in C_5D_5N

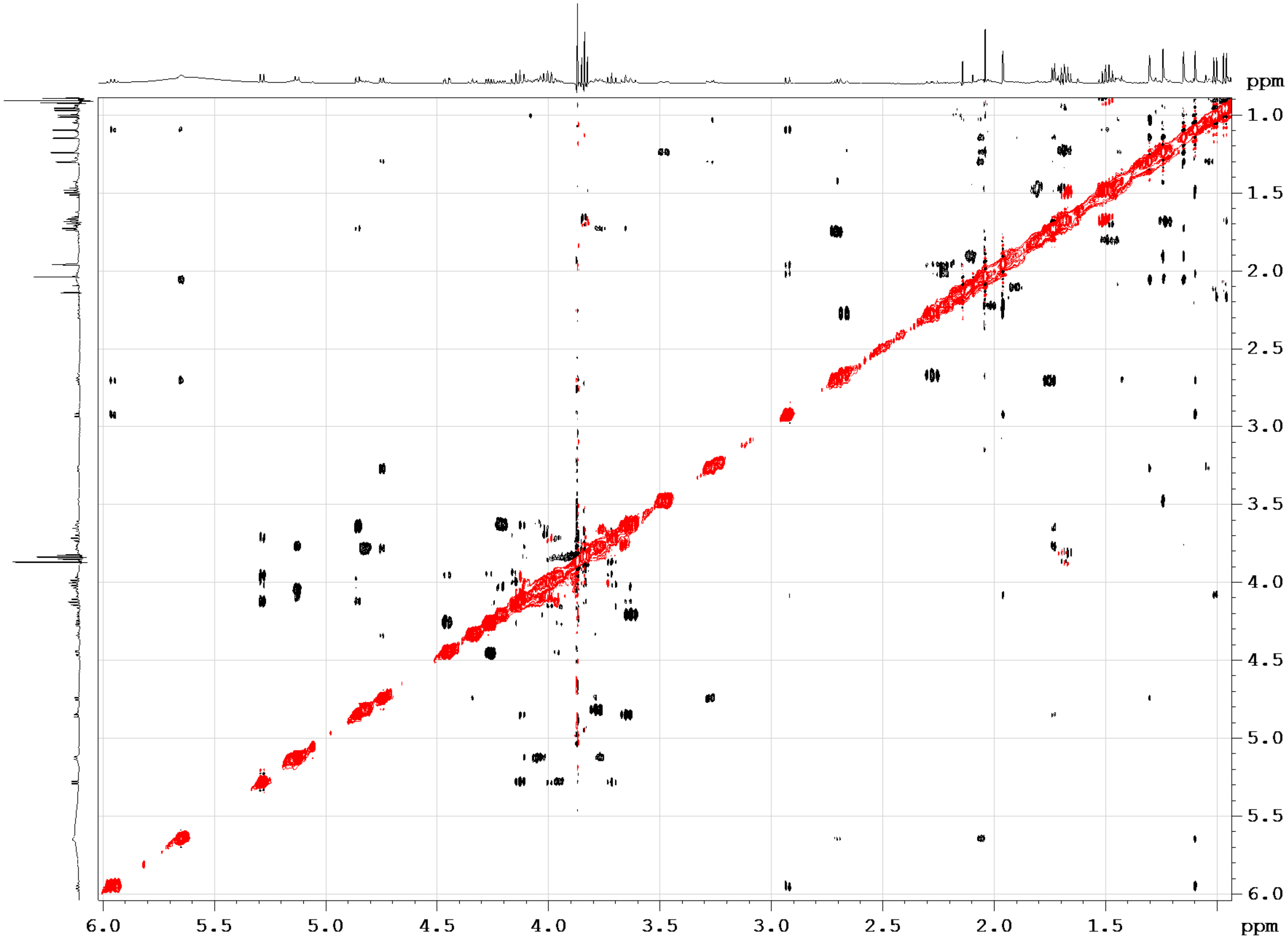

Figure S5. The ROESY (500.12 MHz) spectrum of djakonovioside A (**1**) in C_5D_5N

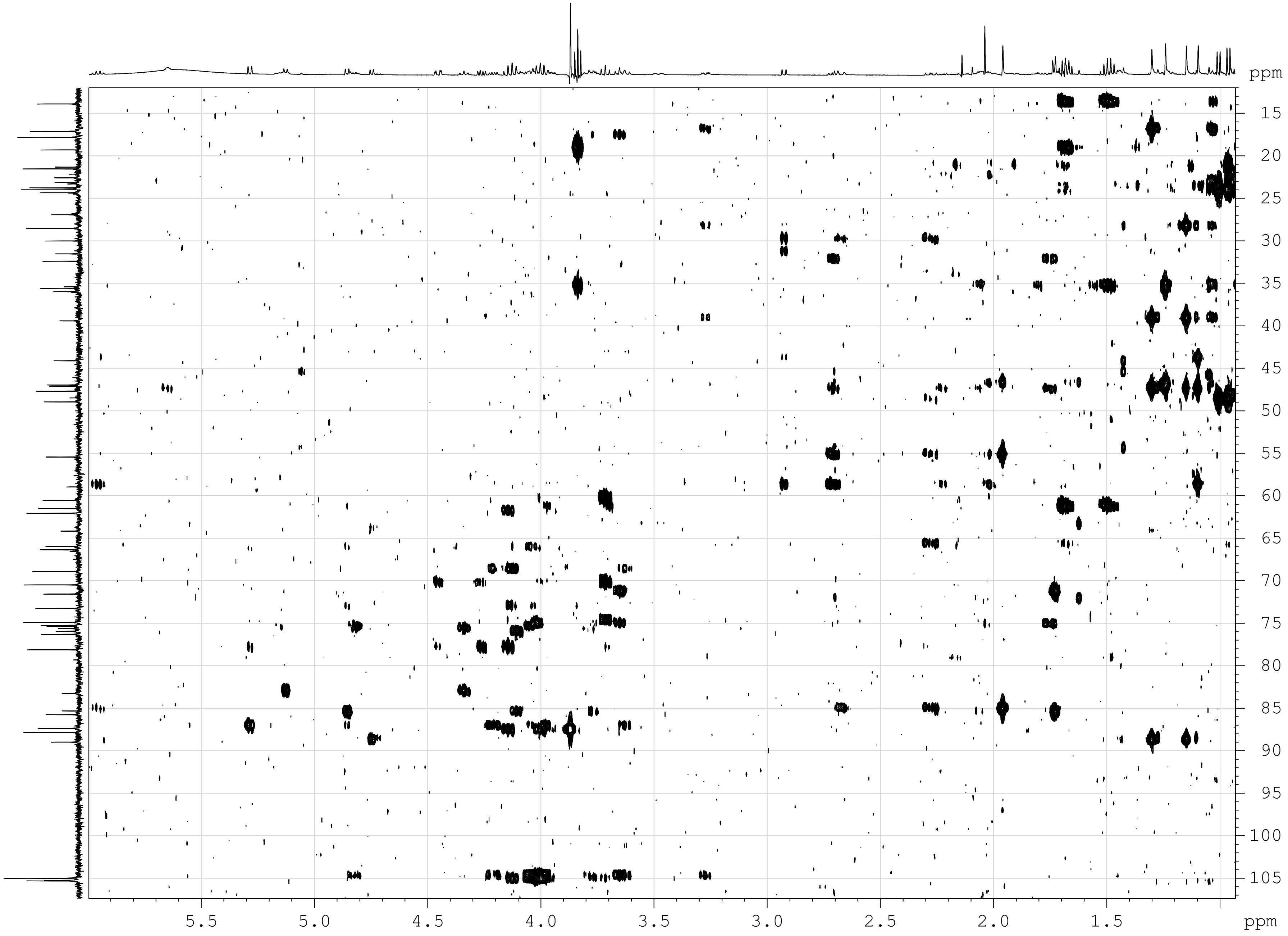

Figure S6. The HMBC (500.12 MHz) spectrum of djakonovioside A (**1**) in C_5D_5N

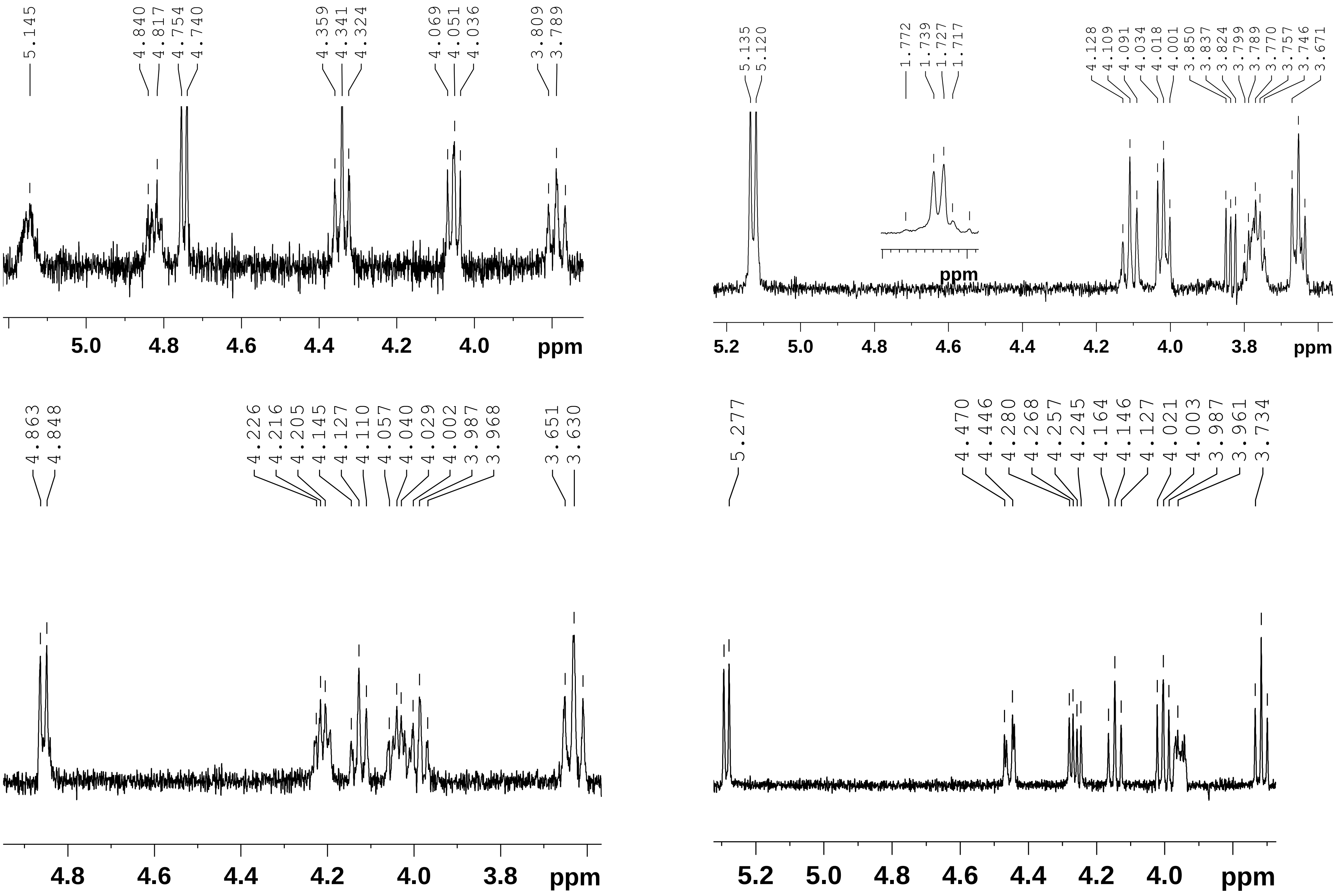

Figure S7. 1 D TOCSY (500.12 MHz) spectra of Xyl1, Qui2, Xyl3, MeGlc4 of djakonovioside A (**1**) in C_5D_5N

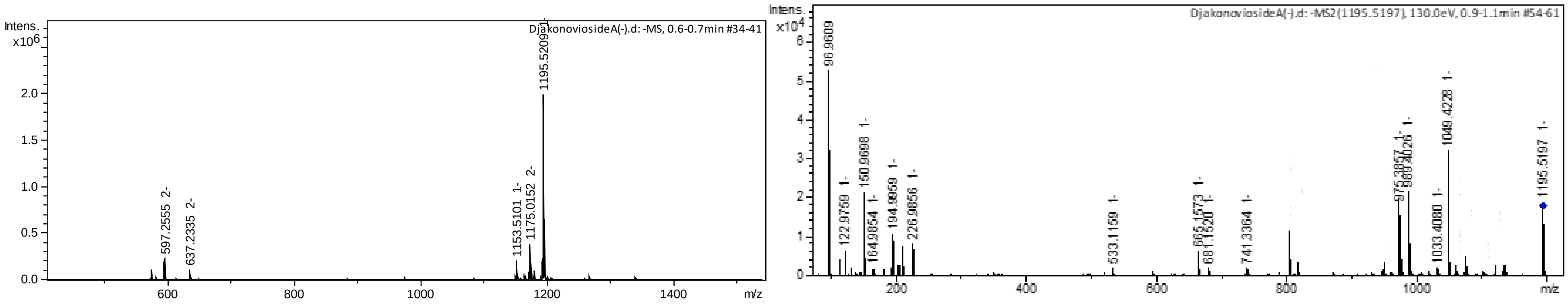

Figure S8. HR-ESI-MS and ESI-MS/MS spectra of djakonovioside A (**1**)

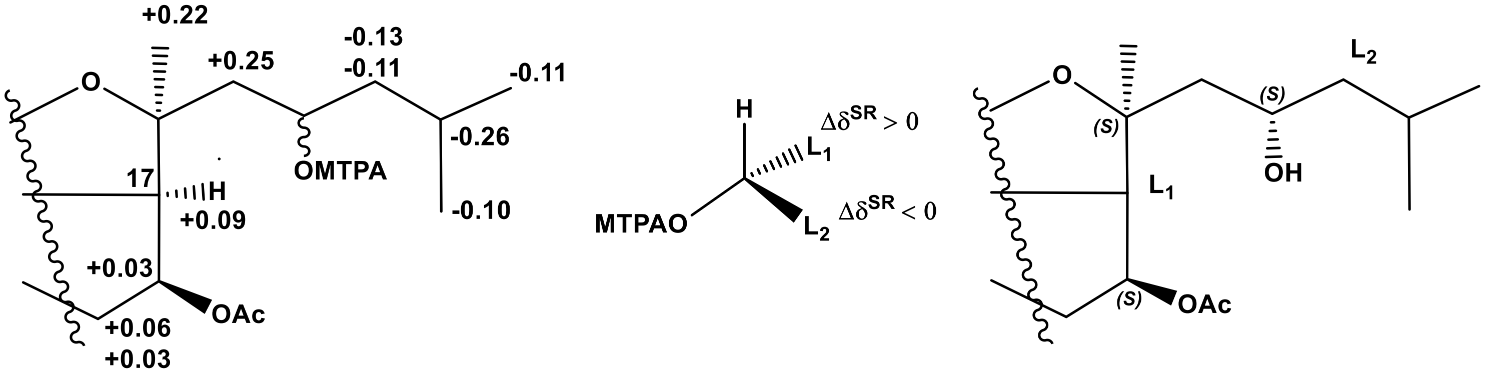

Figure S9. Δ^{SR} sign distribution in 23-O-MTPA esters of **1** and absolute configuration of C-23 stereocenter.

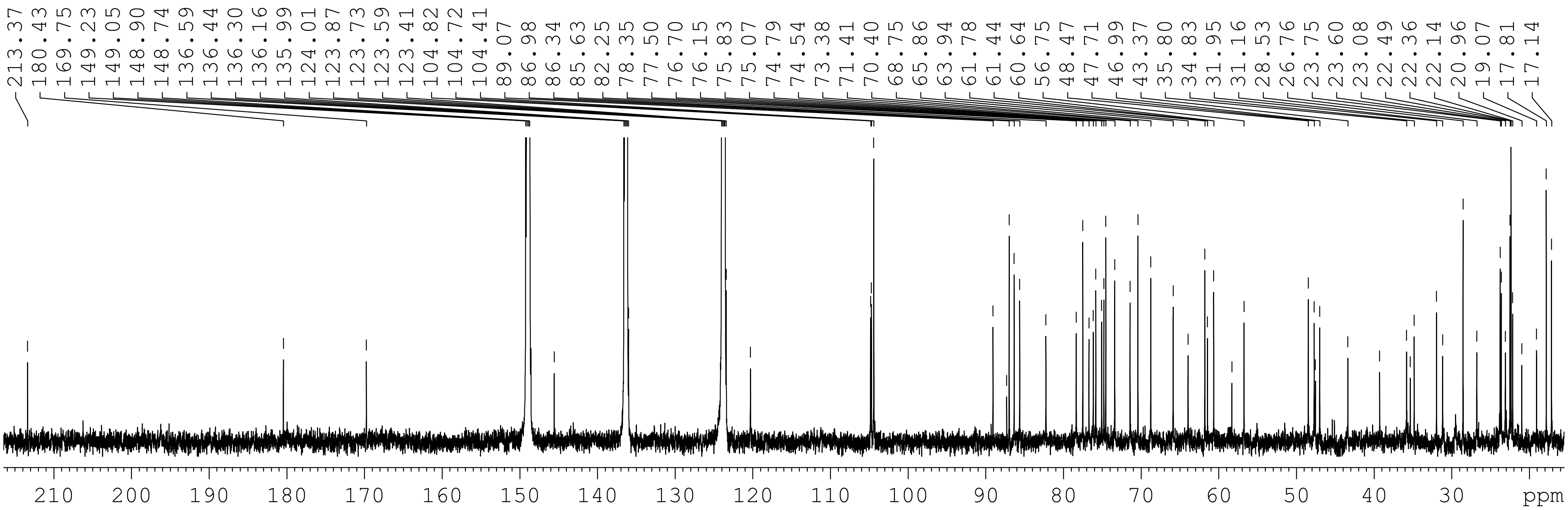

Figure S10. The ^{13}C NMR (125.67 MHz) spectrum of djakonovioside A$_1$ (**2**) in C_5D_5N/D_2O (4/1)

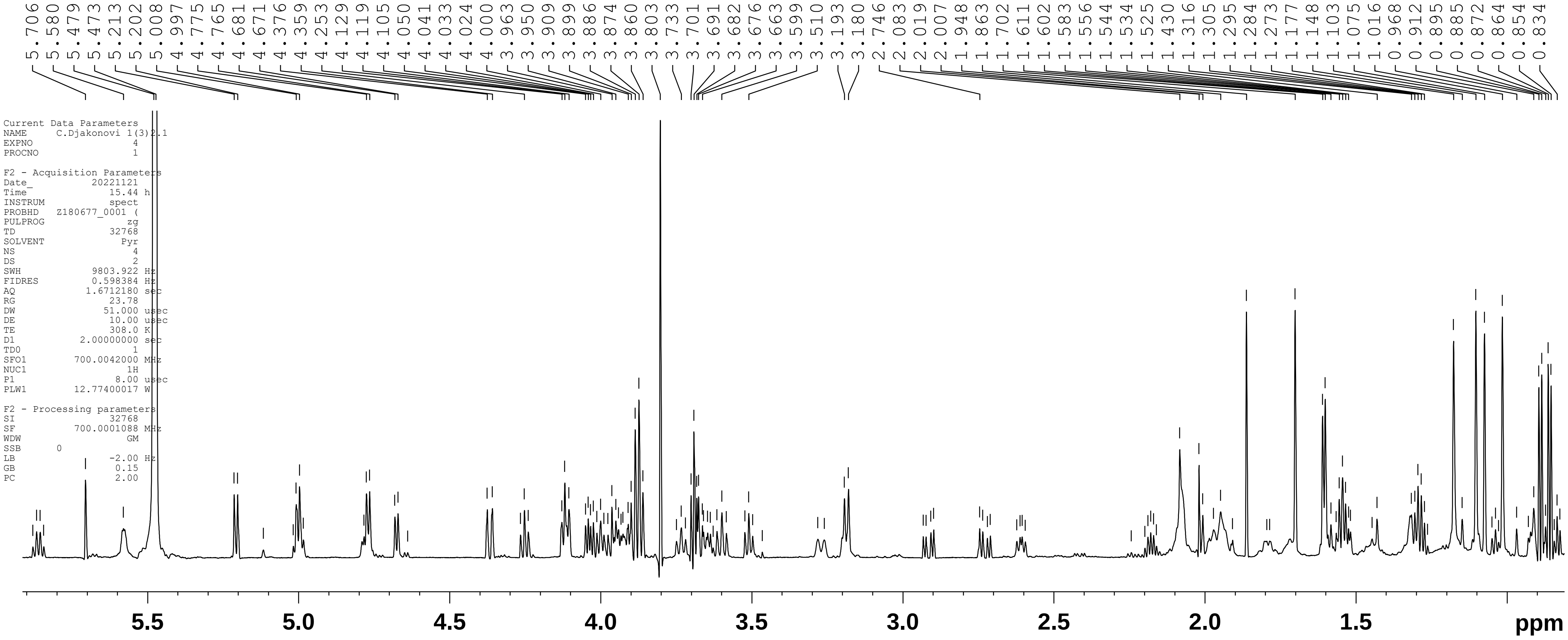

Figure S11. The ^{1}H NMR (500.12 MHz) spectrum of djakonovioside A1 (**2**) in C_5D_5N/D_2O (4/1)

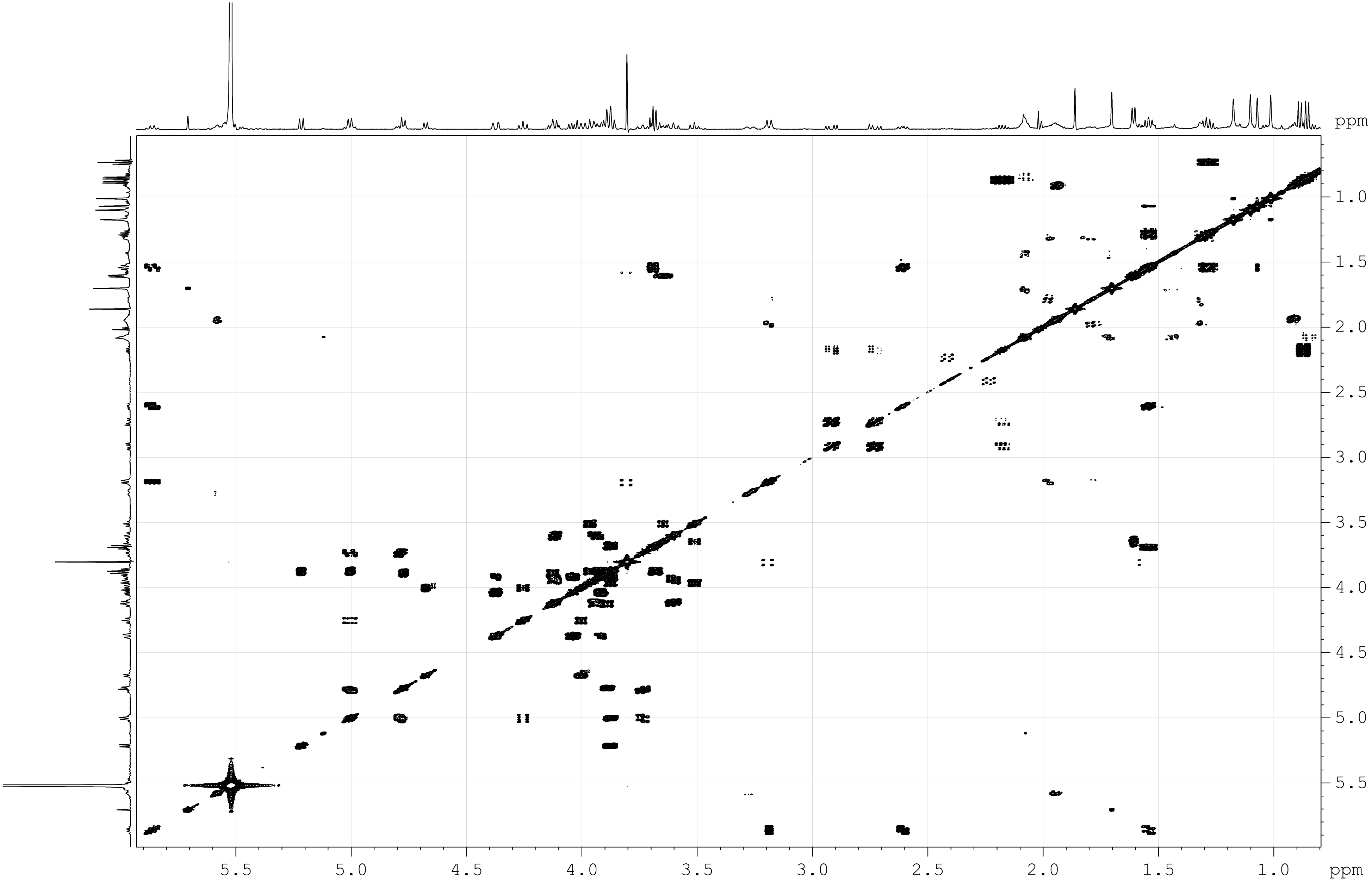

Figure S12. The COSY (500.12 MHz) spectrum of djakonovioside A$_1$ (**2**) in C_5D_5N/D_2O (4/1)

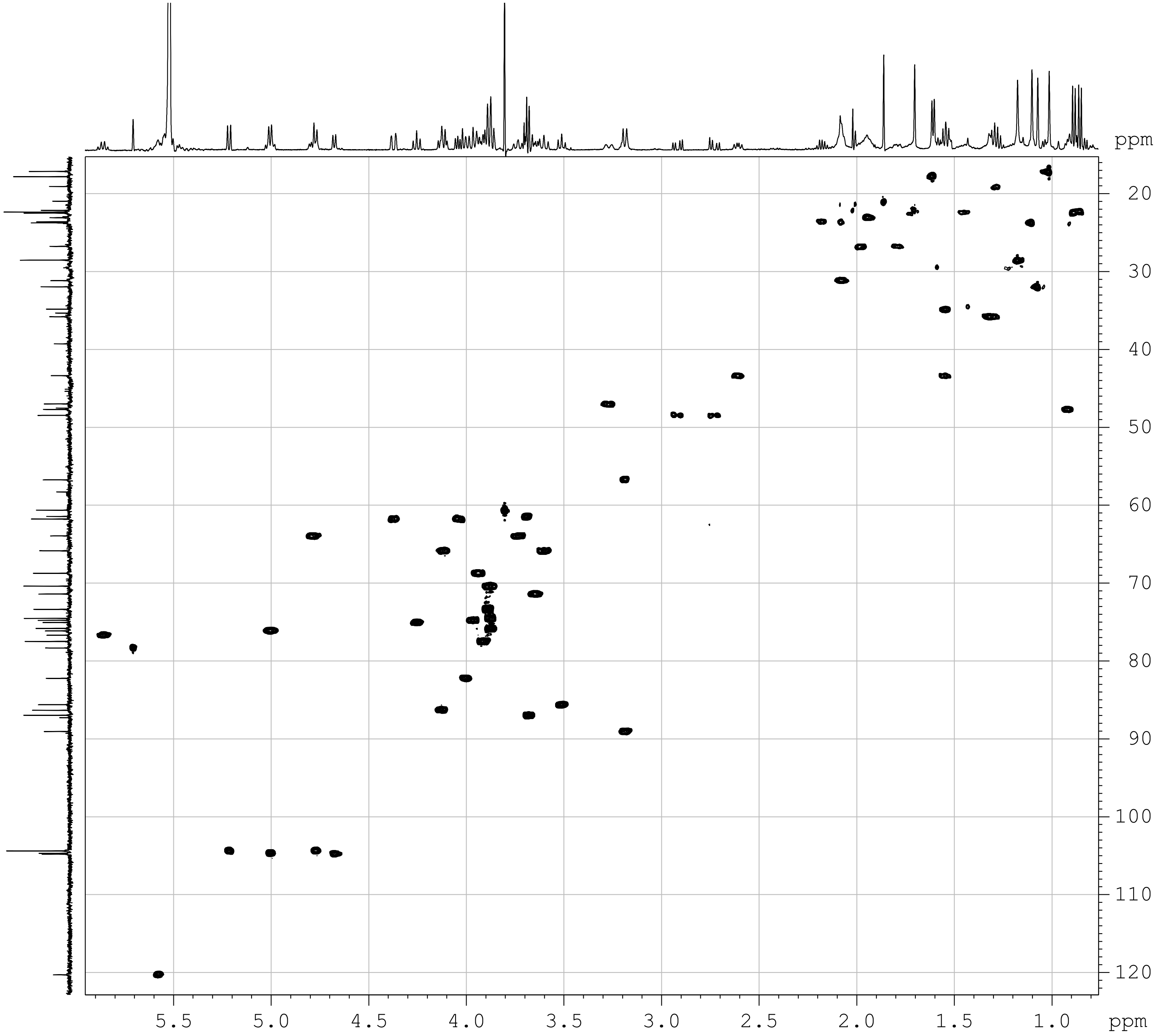

Figure S13. The HSQC (500.12 MHz) spectrum of djakonovioside A1 (**2**) in C_5D_5N/D_2O (4/1)

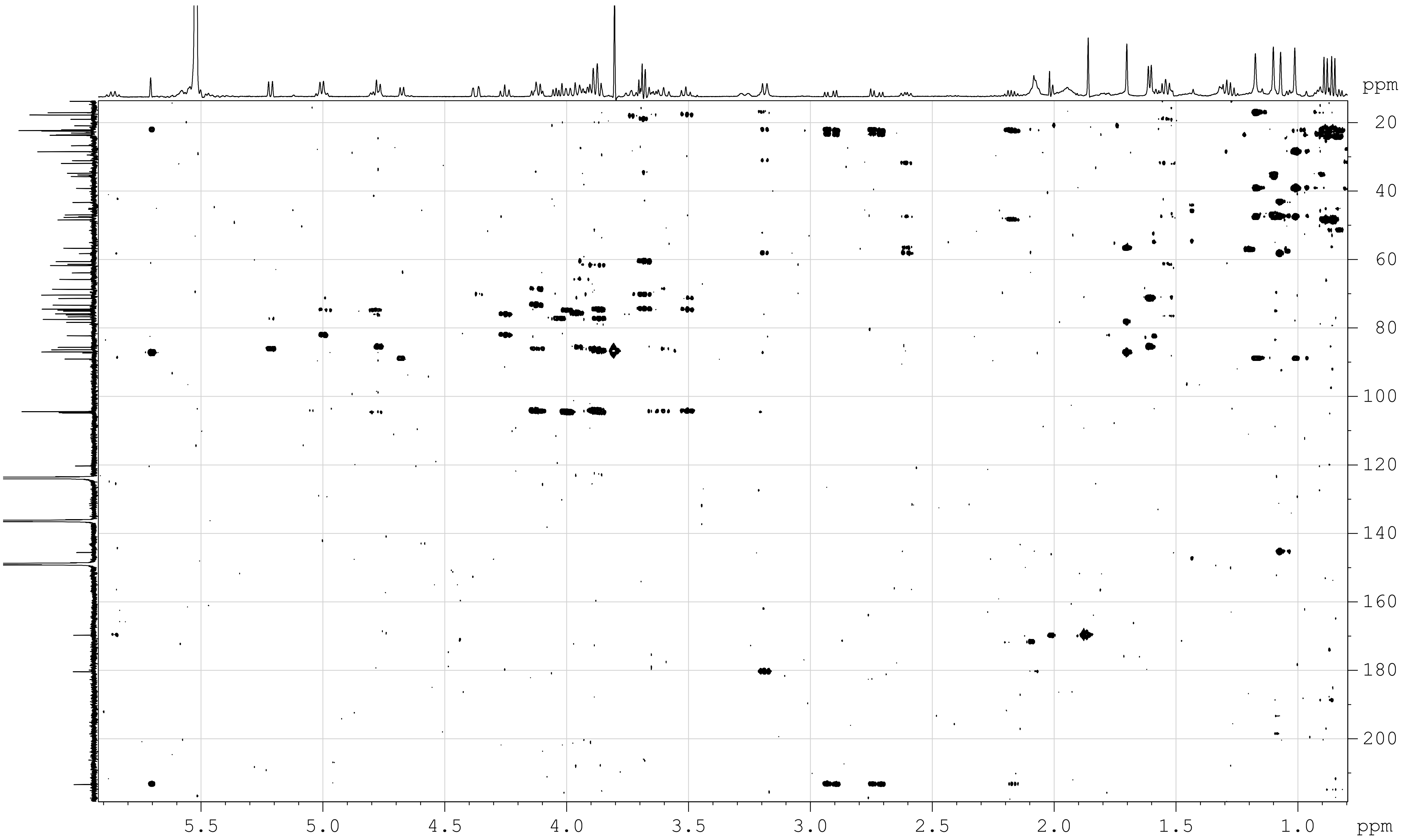

Figure S14. The HMBC (500.12 MHz) spectrum of djakonovioside A$_1$ (**2**) in C_5D_5N/D_2O (4/1)

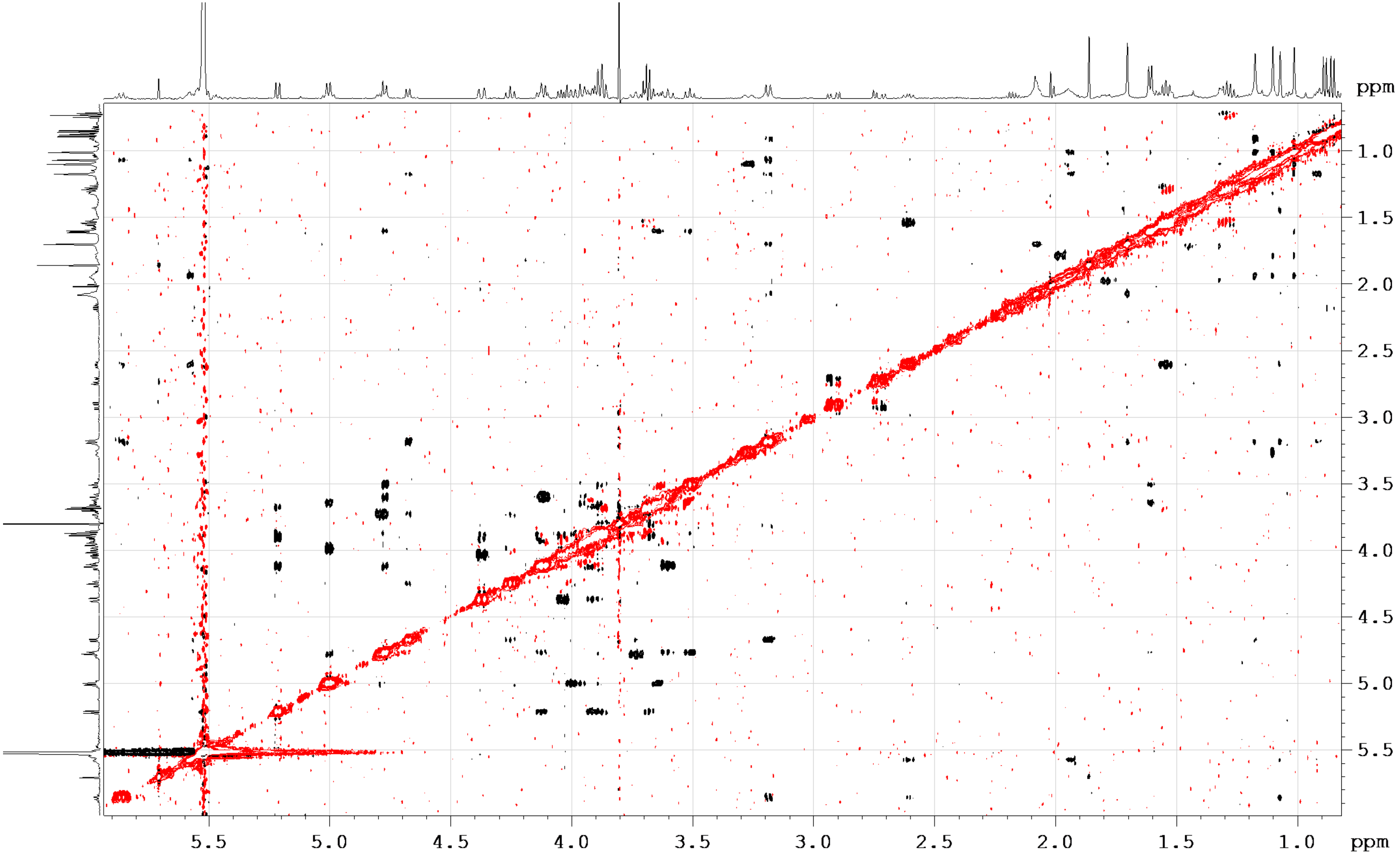

Figure S15. The ROESY (500.12 MHz) spectrum of djakonovioside A1 (**2**) in C_5D_5N/D_2O (4/1)

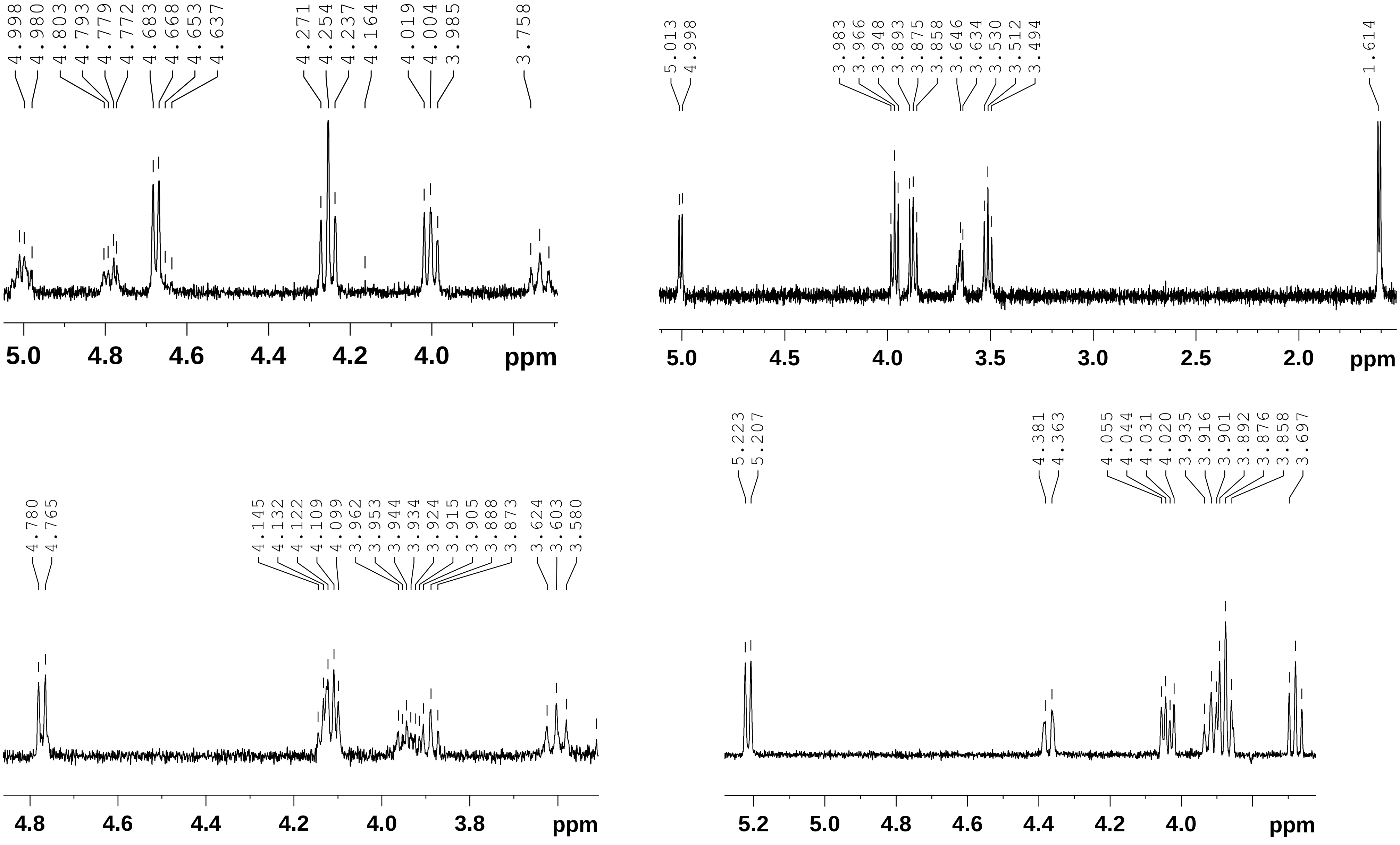

Figure S16. 1D TOCSY (500.12 MHz) spectra of Xyl1, Qui2, Xyl3, MeGlc4 of djakonovioside A1 (**2**) in C_5D_5N/D_2O (4/1)

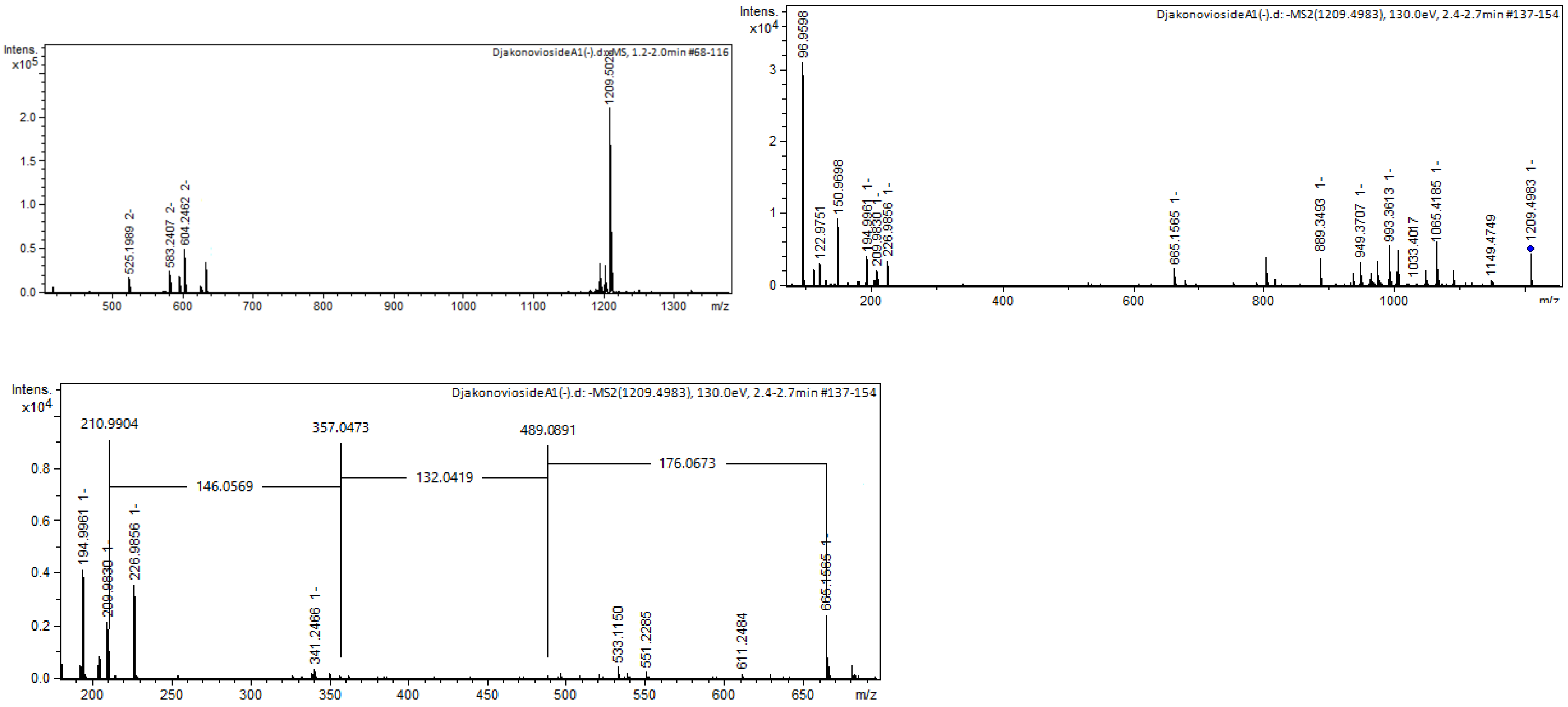

Figure S17. HR-ESI-MS and ESI-MS/MS spectra of djakonovioside A1 (**2**)

Table S1. ^{13}C and ^{1}H NMR chemical shifts and HMBC and ROESY correlations of carbohydrate moiety of djakonovioside A_1 (**2**). [a] Recorded at 176.04 MHz in C_5D_5N. [b] Recorded at 500.13 MHz in C_5D_5N/D_2O. Multiplicity by 1D TOCSY. [c] Bold = interglycosidic positions, [d] Italic – sulfate positions

Atom	δ_C mult.[a,c,d]	δ_H mult. (*J* in Hz)[b]	HMBC	ROESY
Xyl1 (1→C-3)				
1	104.8 CH	4.68 d (7.3)	C: 3	H-3; H-3, 5 Xyl1
2	**82.2** CH	4.00 t (9.1)	C: 1 Qui2; C: 1, 3 Xyl1	H-1 Qui2
3	75.1 CH	4.25 t (9.1)	C: 2, 4 Xyl1	H-1, 5 Xyl1
4	*76.2* CH	5.00 m	C: 3 Xyl1	
5	63.9 CH_2	4.79 dd (5.0; 12.3)	C: 3, 4 Xyl1	
		3.74 t (10.8)		
Qui2 (1→2Xyl1)				
1	104.7 CH	5.01 d (7.7)	C: 2 Xyl1	H-2 Xyl1; H-3, 5 Qui2
2	75.8 CH	3.88 t (9.1)	C: 1, 3 Qui2	H-4 Qui2
3	74.8 CH	3.97 t (9.1)	C: 2, 4 Qui2	
4	**85.6** CH	3.51 t (9.1)	C: 1 Xyl3; C: 3, 5 Qui2	H-1 Xyl3
5	71.4 CH	3.65 dd (5.6; 9.1)		H-1 Qui2
6	17.8 CH_3	1.61 d (5.6)	C: 4, 5 Qui2	
Xyl3 (1→4Qui2)				
1	104.4 CH	4.77 d (7.7)	C: 4 Qui2	H-4 Qui2; H-3, 5 Xyl3
2	73.4 CH	3.89 t (8.8)	C: 3 Xyl3	
3	**86.3** CH	4.13 t (8.8)	C: 1 MeGlc4; C: 2, 4 Xyl3	H-1 MeGlc4; H-1 Xyl3
4	68.7 CH	3.94 m	C: 5 Xyl3	
5	65.9 CH_2	4.12 dd (5.5; 11.8)	C: 3 Xyl3	
		3.60 t (11.3)	C: 1, 3, 4 Xyl3	H-1 Xyl3
MeGlc4 (1→3Xyl3)				
1	104.4 CH	5.21 d (7.3)	C: 3 Xyl3	H-3 Xyl3; H-3, 5 MeGlc4
2	74.5 CH	3.88 t (8.4)	C: 1, 3 MeGlc4	
3	87.0 CH	3.68 t (8.4)	C: 2, 4 MeGlc4; OMe	H-1, 5 MeGlc4; OMe
4	70.4 CH	3.89 t (8.4)	C: 3, 5 MeGlc4	
5	77.5 CH	3.92 t (9.0)	C: 4, 6 MeGlc4	H-1 MeGlc4
6	61.8 CH_2	4.37 dd (2.2; 11.2)	C: 4 MeGlc4	
		4.04 dd (5.6; 11.2)	C: 5 MeGlc4	
OMe	60.6 CH_3	3.80 s	C: 3 MeGlc4	

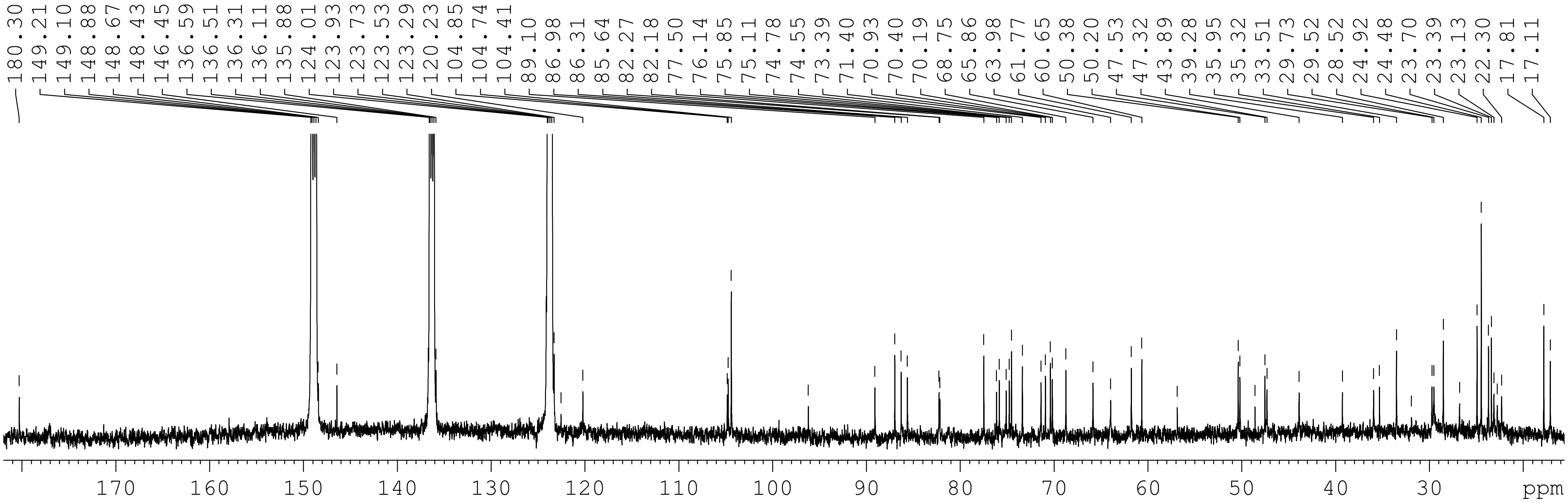

Figure S18. The ^{13}C NMR (125.67 MHz) spectrum of djakonovioside A_2 (**3**) in C_5D_5N/D_2O (4/1)

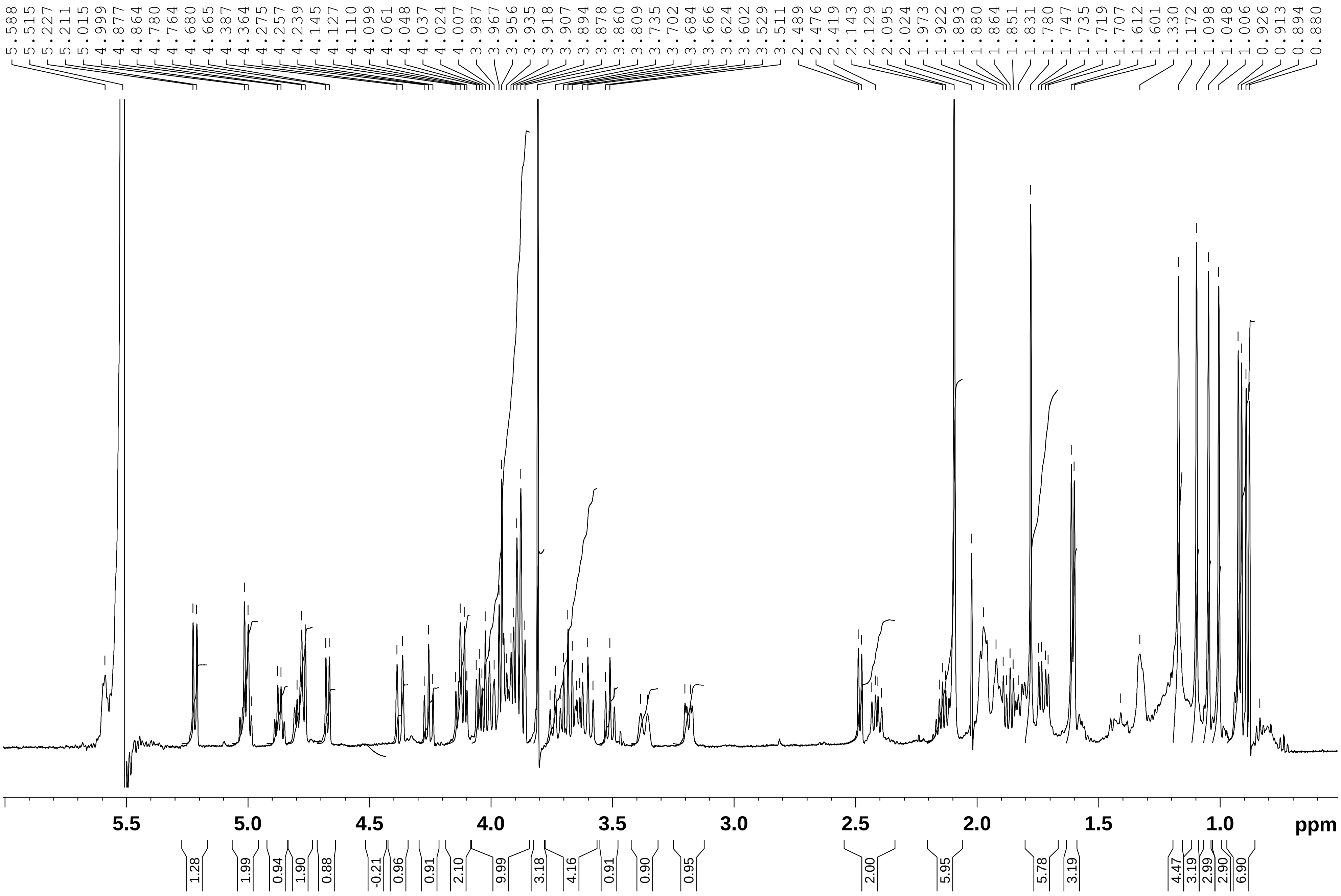

Figure S19. The ^{1}H NMR (500.12 MHz) spectrum of djakonovioside A2 (**3**) in C_5D_5N/D_2O (4/1)

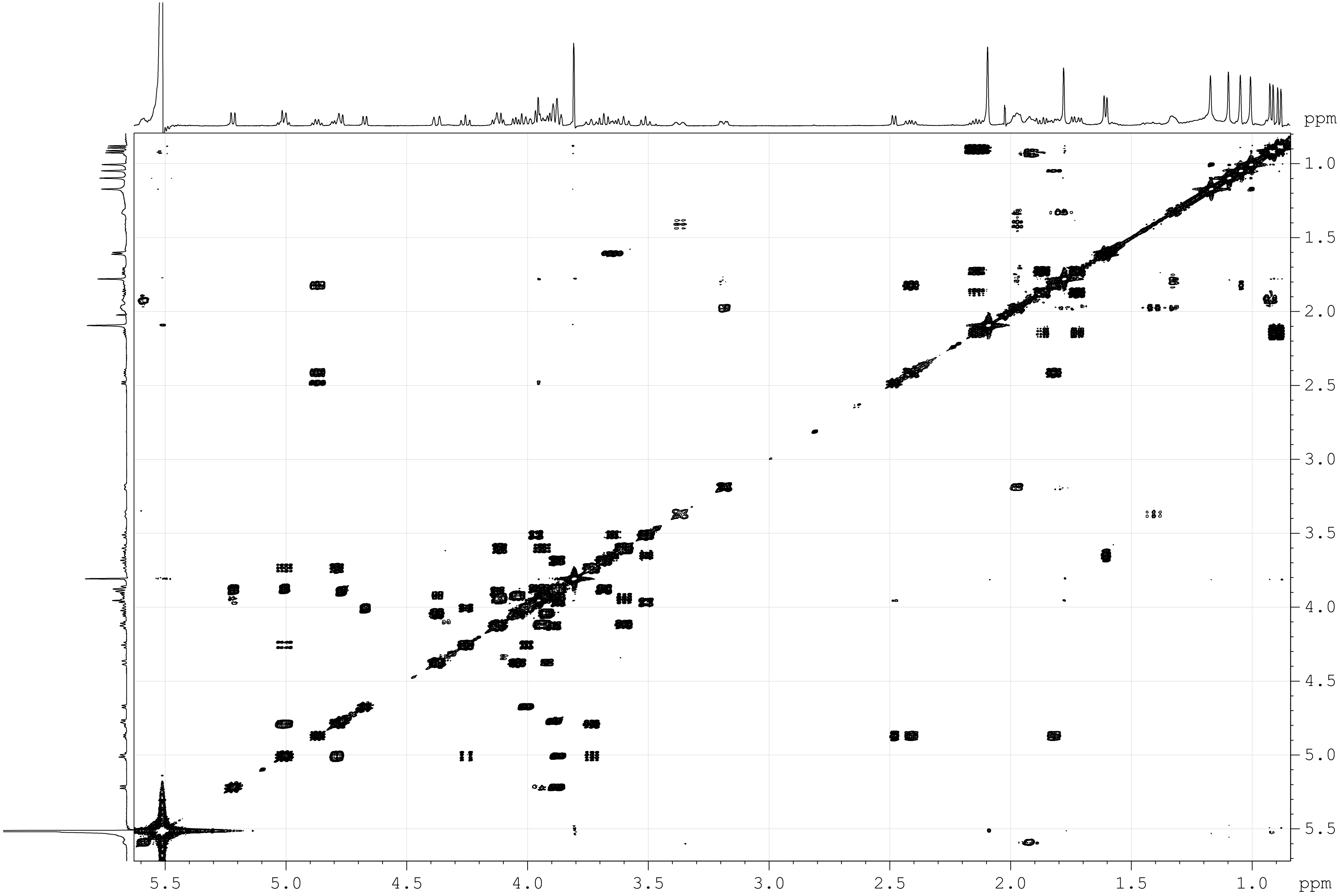

Figure S20. The COSY (500.12 MHz) spectrum of djakonovioside A_2 (**3**) in C_5D_5N/D_2O (4/1)

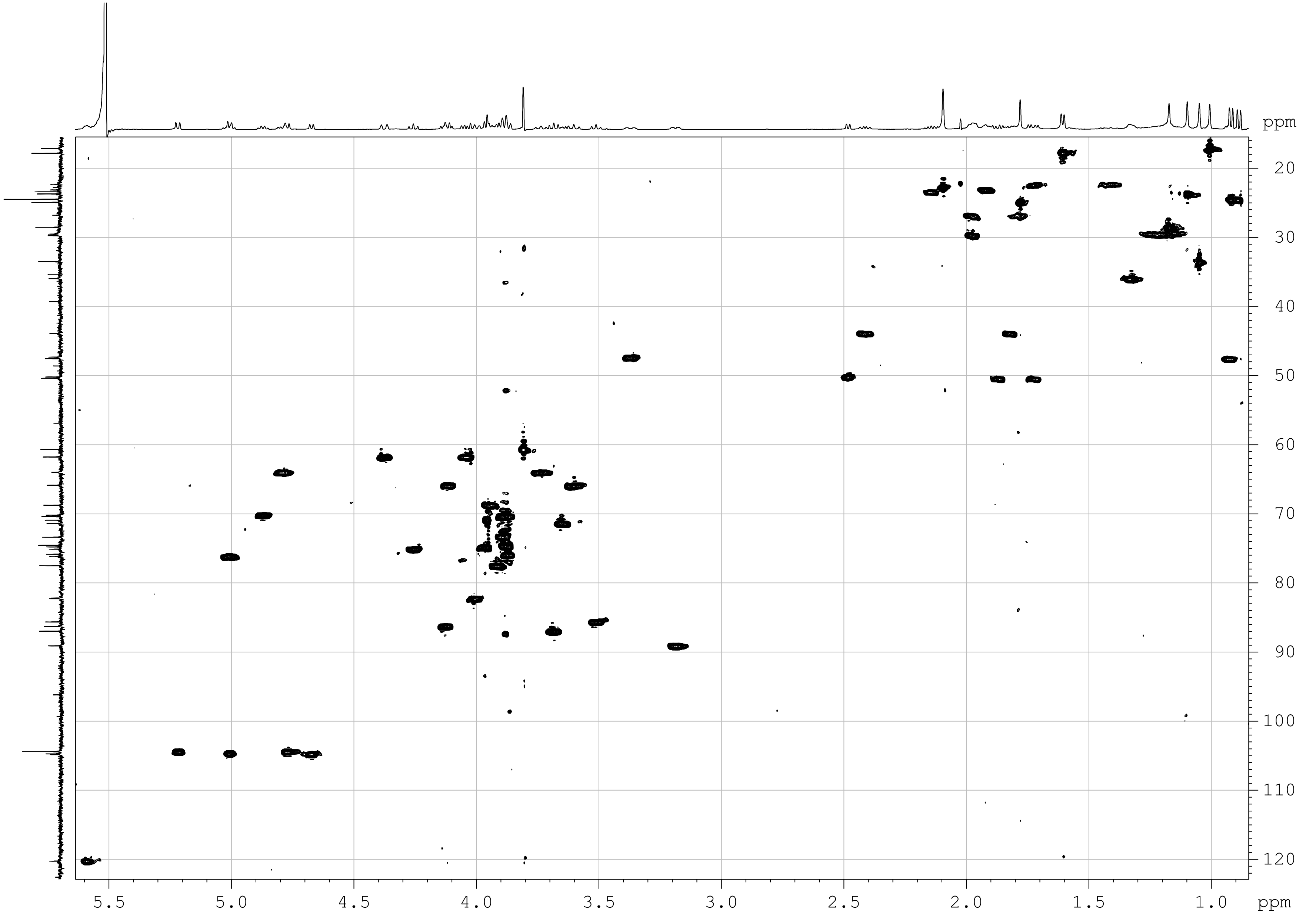

Figure S21. The HSQC (500.12 MHz) spectrum of djakonovioside A_2 (**3**) in C_5D_5N/D_2O (4/1)

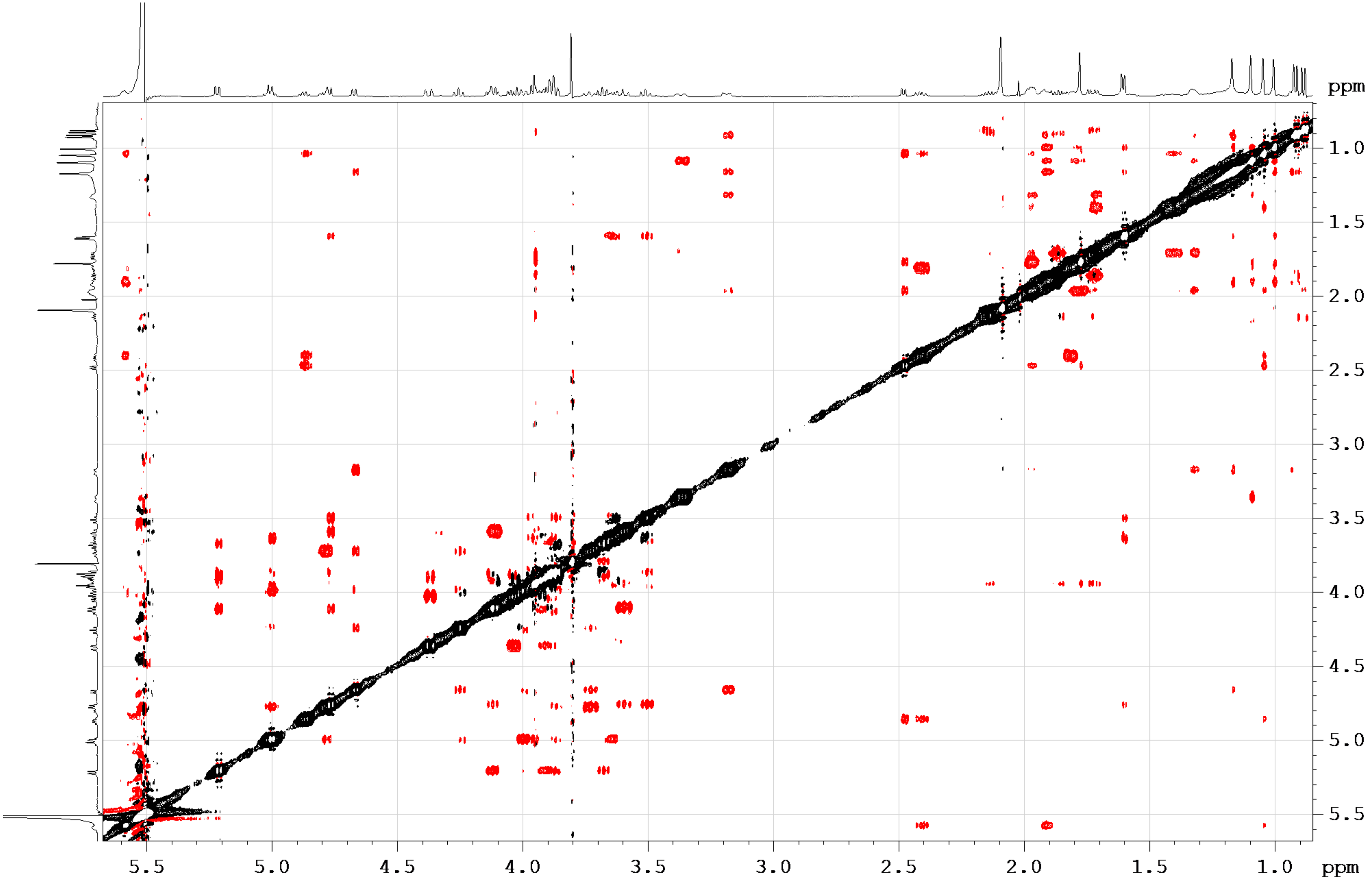

Figure S22. The ROESY (500.12 MHz) spectrum of djakonovioside A2 (**3**) in C_5D_5N/D_2O (4/1)

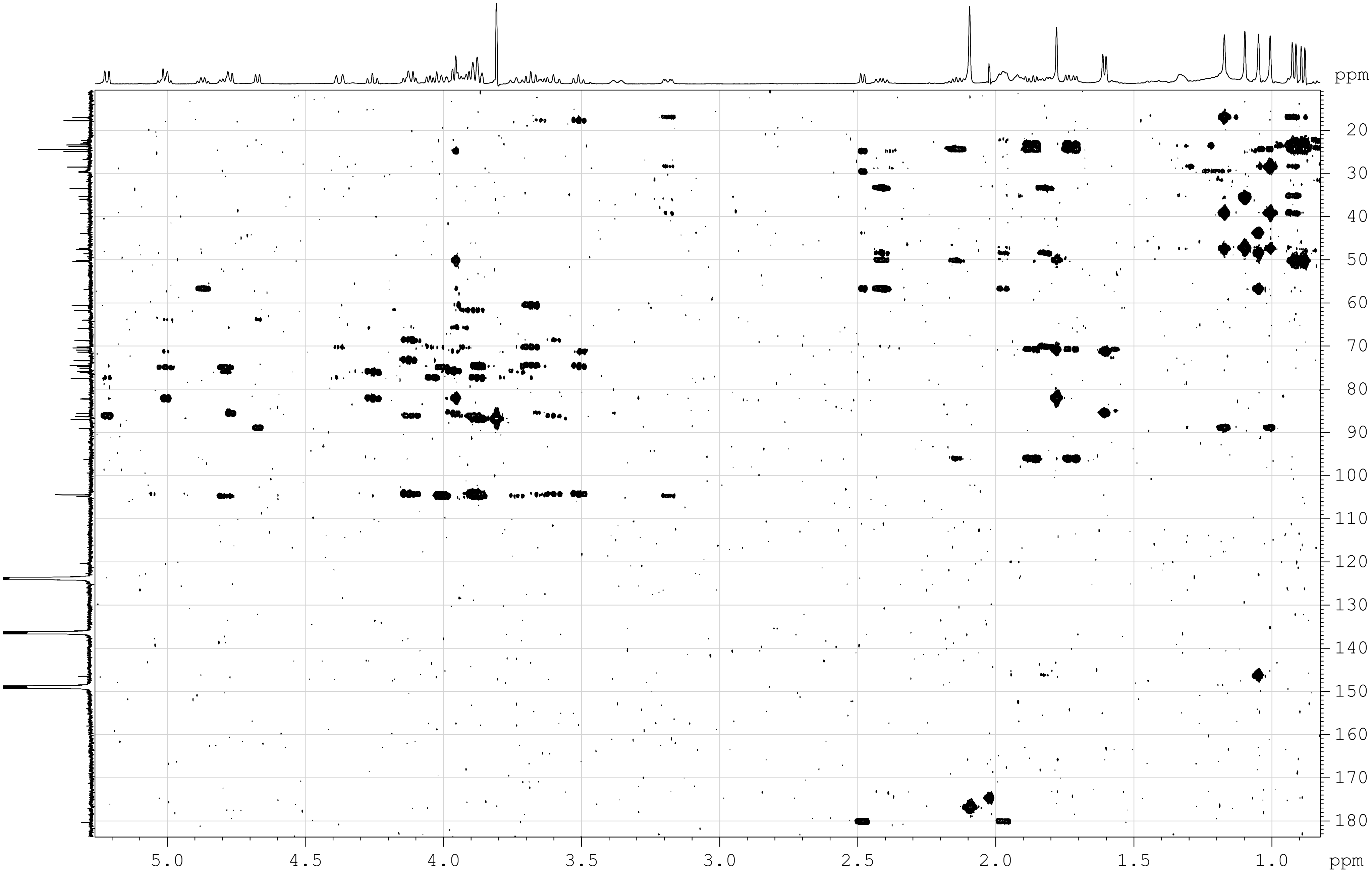

Figure S23. The HMBC (500.12 MHz) spectrum of djakonovioside A_2 (**3**) in C_5D_5N/D_2O (4/1)

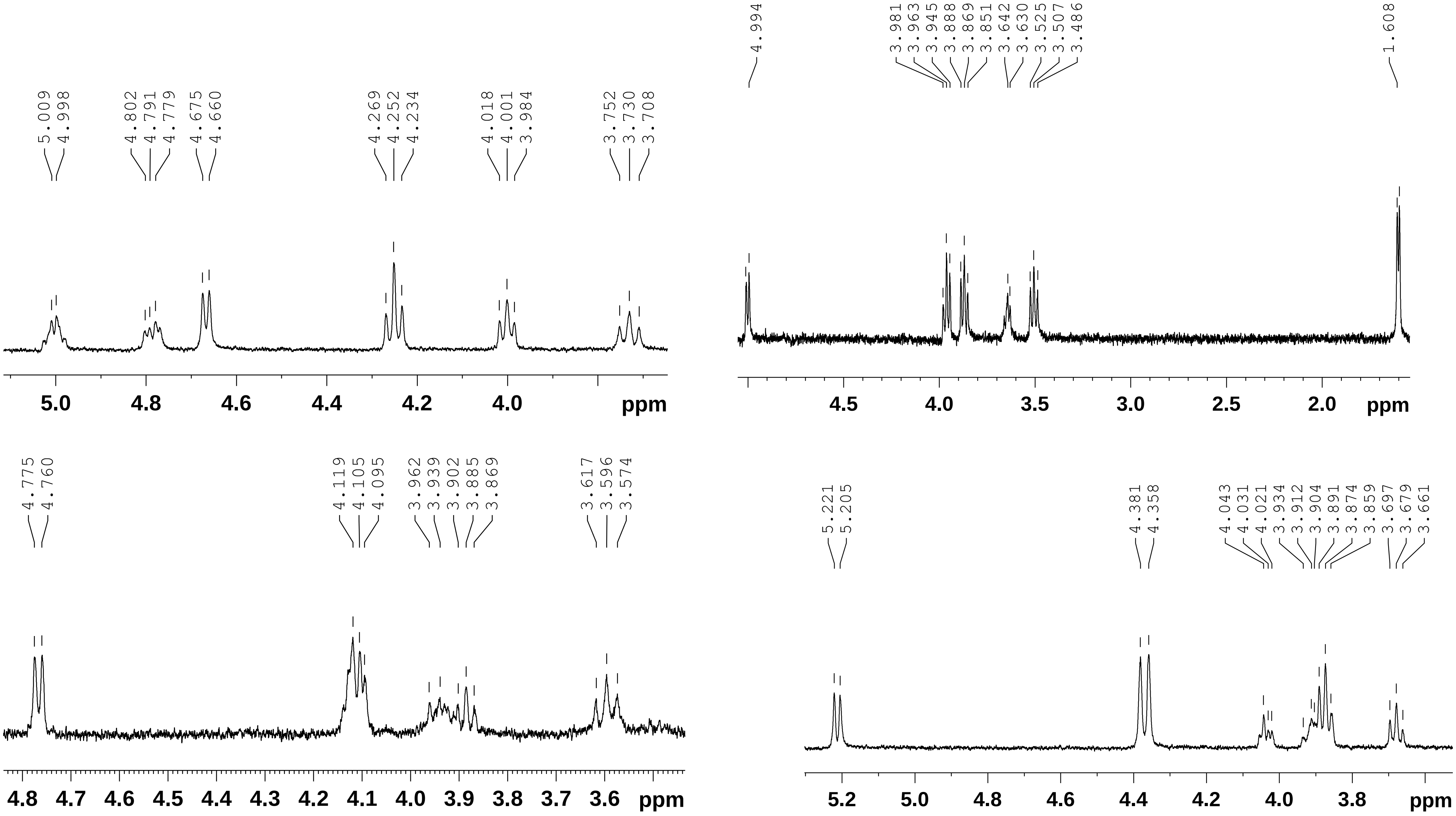

Figure S24. 1D TOCSY (500.12 MHz) spectra of Xyl1, Qui2, Xyl3, MeGlc4 of djakonovioside A$_2$ (**3**) in C_5D_5N/D_2O (4/1)

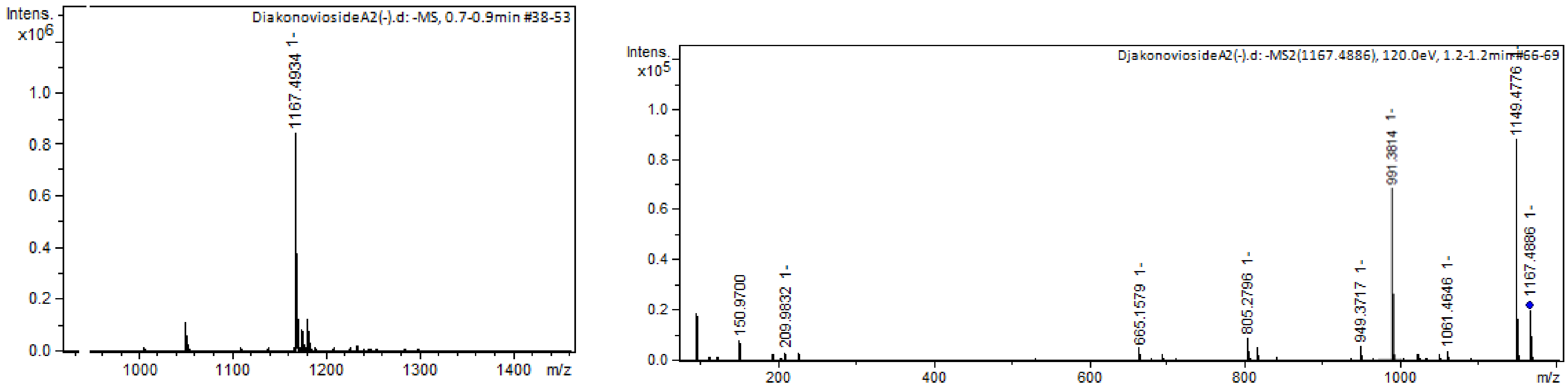

Figure S25. HR-ESI-MS and ESI-MS/MS spectra of djakonovioside A_2 (**3**)

Table S2. ^{13}C and ^{1}H NMR chemical shifts and HMBC and ROESY correlations of carbohydrate moiety of **djakonovioside A$_2$**. [a] Recorded at 176.04 MHz in C_5D_5N. [b] Recorded at 500.13 MHz in C_5D_5N/D_2O. Multiplicity by 1D TOCSY. [c] Bold = interglycosidic positions, [d] Italic – sulfate positions

Atom	δC mult.[a,c,d]	δH mult. (*J* in Hz)[b]	HMBC	ROESY
Xyl1 (1→C-3)				
1	104.8 CH	4.68 d (7.3)	C: 3; C: 3, 5 Xyl1	H-3; H-3, 5 Xyl1
2	**82.2** CH	4.00 t (9.1)	C: 1 Qui2; C: 1, 3 Xyl1	H-1 Qui2
3	75.1 CH	4.25 t (9.1)	C: 2, 4 Xyl1	H-1, 5 Xyl1
4	*76.1* CH	5.00 m	C: 3 Xyl1	
5	64.0 CH$_2$	4.79 dd (5.0; 12.3)	C: 3, 4 Xyl1	
		3.73 t (10.8)		H-1 Xyl1
Qui2 (1→2Xyl1)				
1	104.7 CH	5.00 d (7.7)	C: 2 Xyl1	H-2 Xyl1; H-3, 5 Qui2
2	75.8 CH	3.87 t (9.1)	C: 1, 3 Qui2	H-4 Qui2
3	74.8 CH	3.96 t (9.1)	C: 2, 4 Qui2	
4	**85.6** CH	3.51 t (9.1)	C: 1 Xyl3; C: 3, 5 Qui2	H-1 Xyl3
5	71.4 CH	3.64 dd (5.6; 9.1)		H-1 Qui2
6	17.8 CH$_3$	1.61 d (5.6)	C: 4, 5 Qui2	
Xyl3 (1→4Qui2)				
1	104.4 CH	4.77 d (7.7)	C: 4 Qui2	H-4 Qui2; H-3, 5 Xyl3
2	73.4 CH	3.88 t (8.8)	C: 3 Xyl3	
3	**86.3** CH	4.12 t (8.8)	C: 1 MeGlc4; C: 2, 4 Xyl3	H-1 MeGlc4; H-1 Xyl3
4	68.7 CH	3.94 m	C: 5 Xyl3	
5	65.9 CH$_2$	4.11 dd (5.5; 11.8)	C: 3 Xyl3	
		3.60 t (11.3)	C: 1, 3, 4 Xyl3	H-1 Xyl3
MeGlc4 (1→3Xyl3)				
1	104.4 CH	5.21 d (7.3)	C: 3 Xyl3	H-3 Xyl3; H-3, 5
2	74.5 CH	3.87 t (8.4)	C: 1, 3 MeGlc4	
3	87.0 CH	3.68 t (8.4)	C: 2, 4 MeGlc4; OMe	H-1, 5 MeGlc4; OMe
4	70.4 CH	3.87 t (8.4)	C: 3, 5 MeGlc4	
5	77.5 CH	3.91 m	C: 4, 6 MeGlc4	H-1 MeGlc4
6	61.8 CH$_2$	4.37 dd (2.2; 11.2)	C: 4 MeGlc4	
		4.04 dd (5.6; 11.2)	C: 5 MeGlc4	
OMe	60.6 CH$_3$	3.80 s	C: 3 MeGlc4	

.

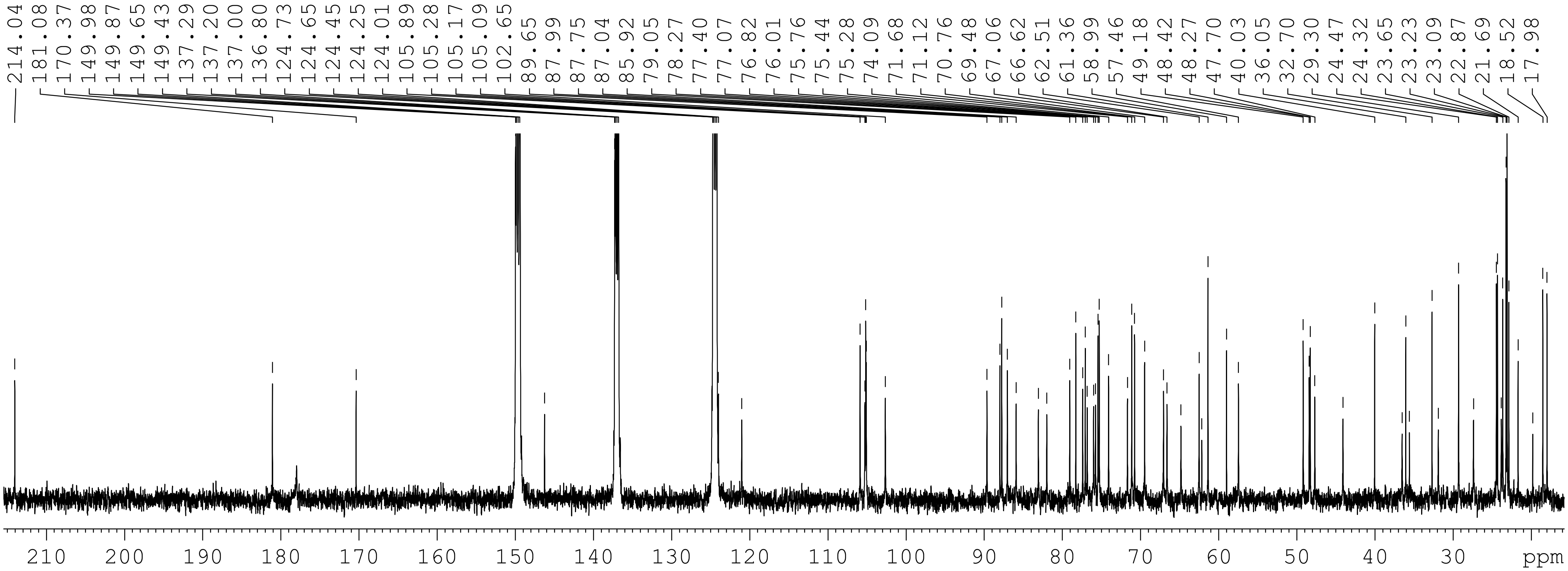

Figure S26. The ^{13}C NMR (125.67 MHz) spectrum of djakonovioside B_1 (**4**) in C_5D_5N/D_2O (4/1)

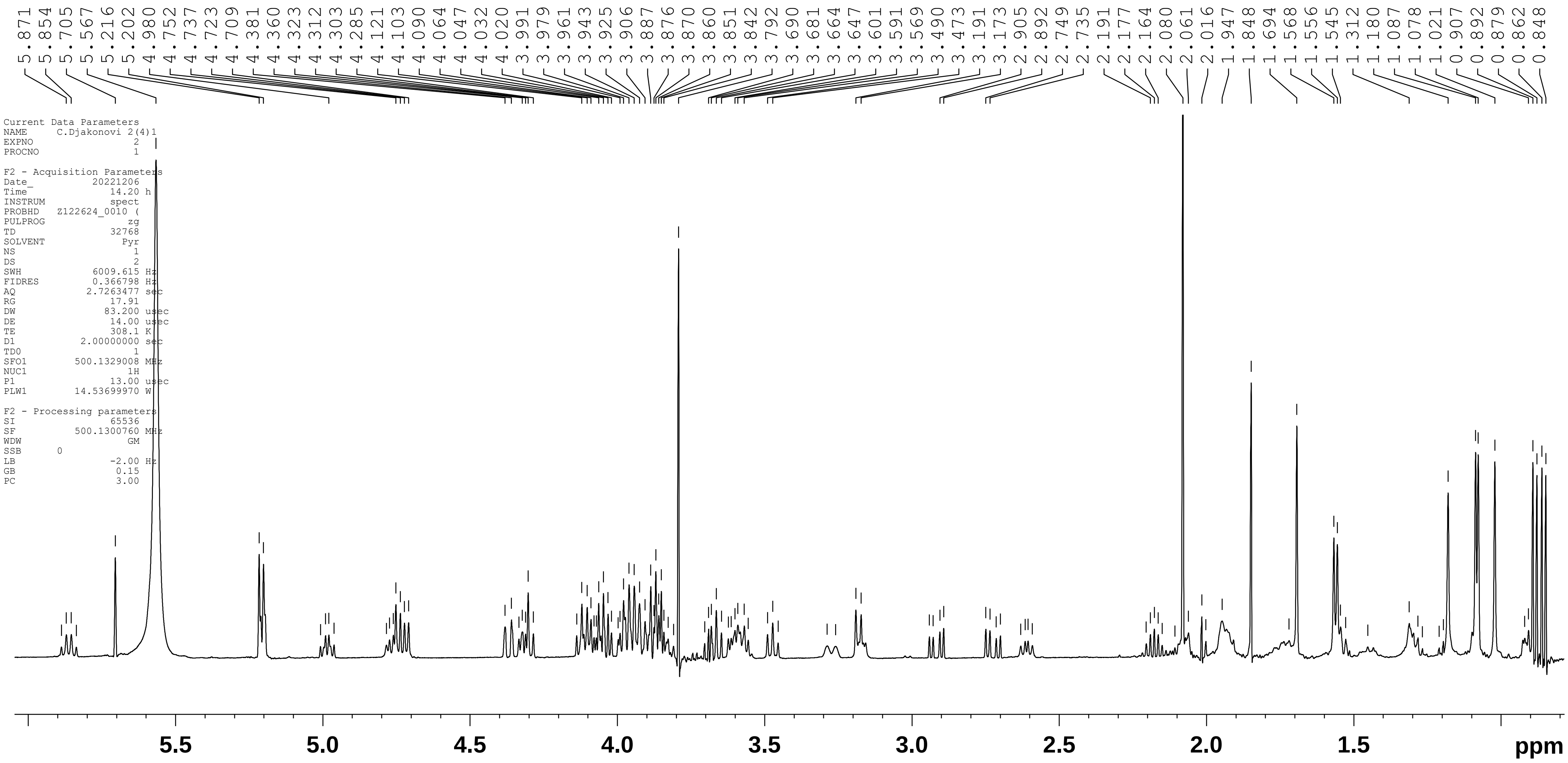

Figure S27. The ^{1}H NMR (500.12 MHz) spectrum of djakonovioside B_1 (**4**) in C_5D_5N/D_2O (4/1)

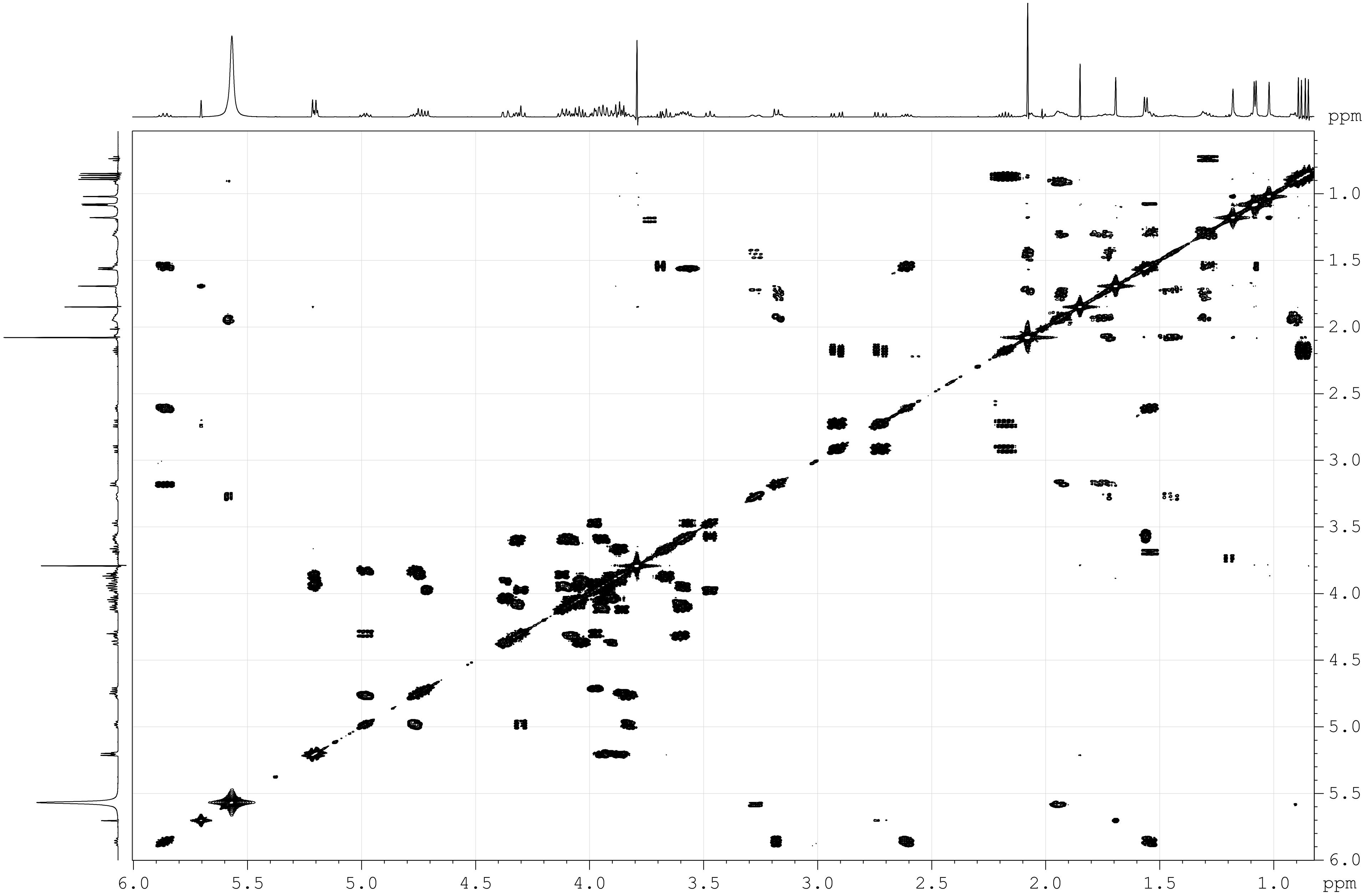

Figure S28. The COSY (500.12 MHz) spectrum of djakonovioside B_1 (**4**) in C_5D_5N/D_2O (4/1)

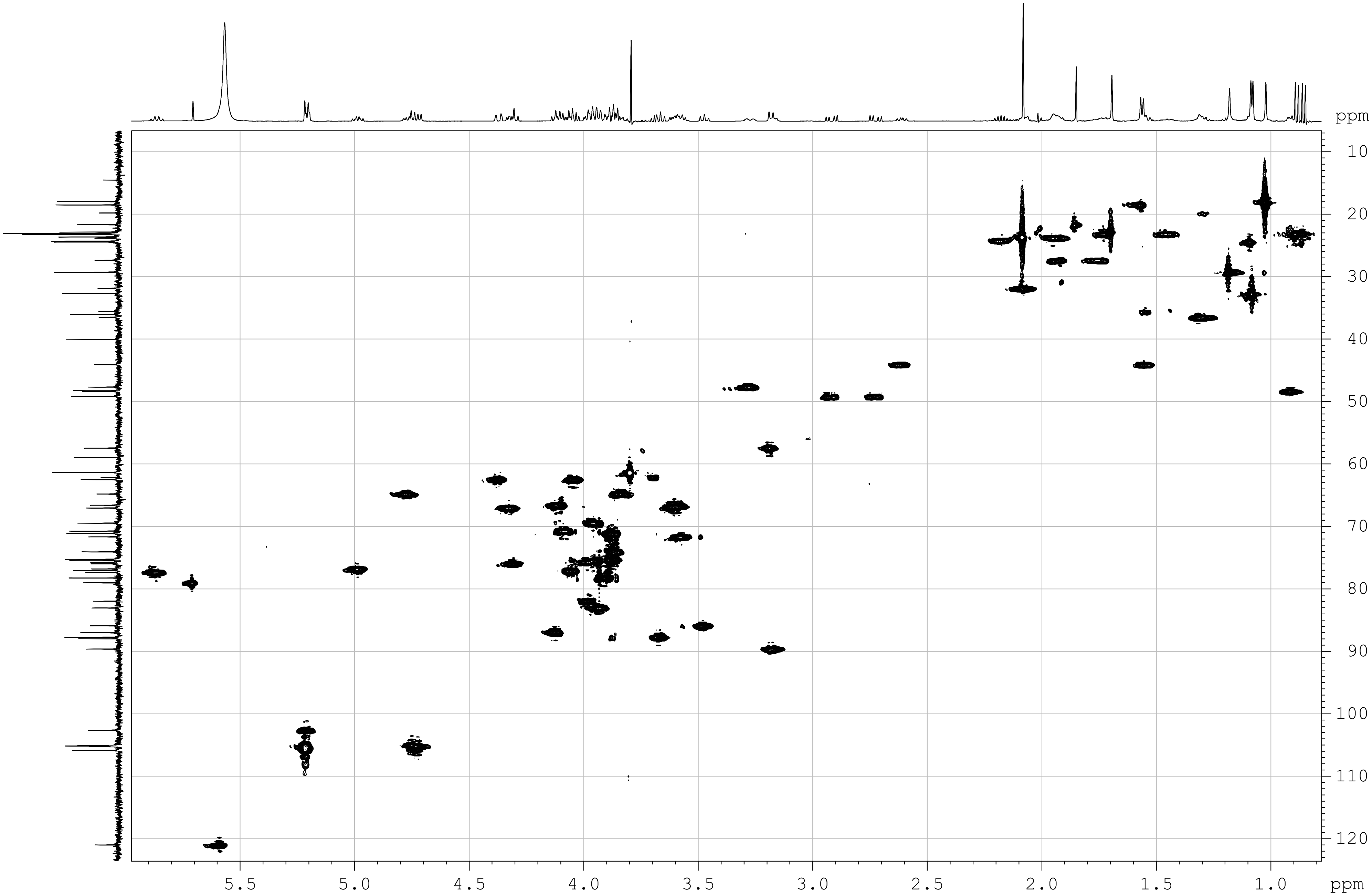

Figure S29. The HSQC (500.12 MHz) spectrum of djakonovioside B_1 (**4**) in C_5D_5N/D_2O (4/1)

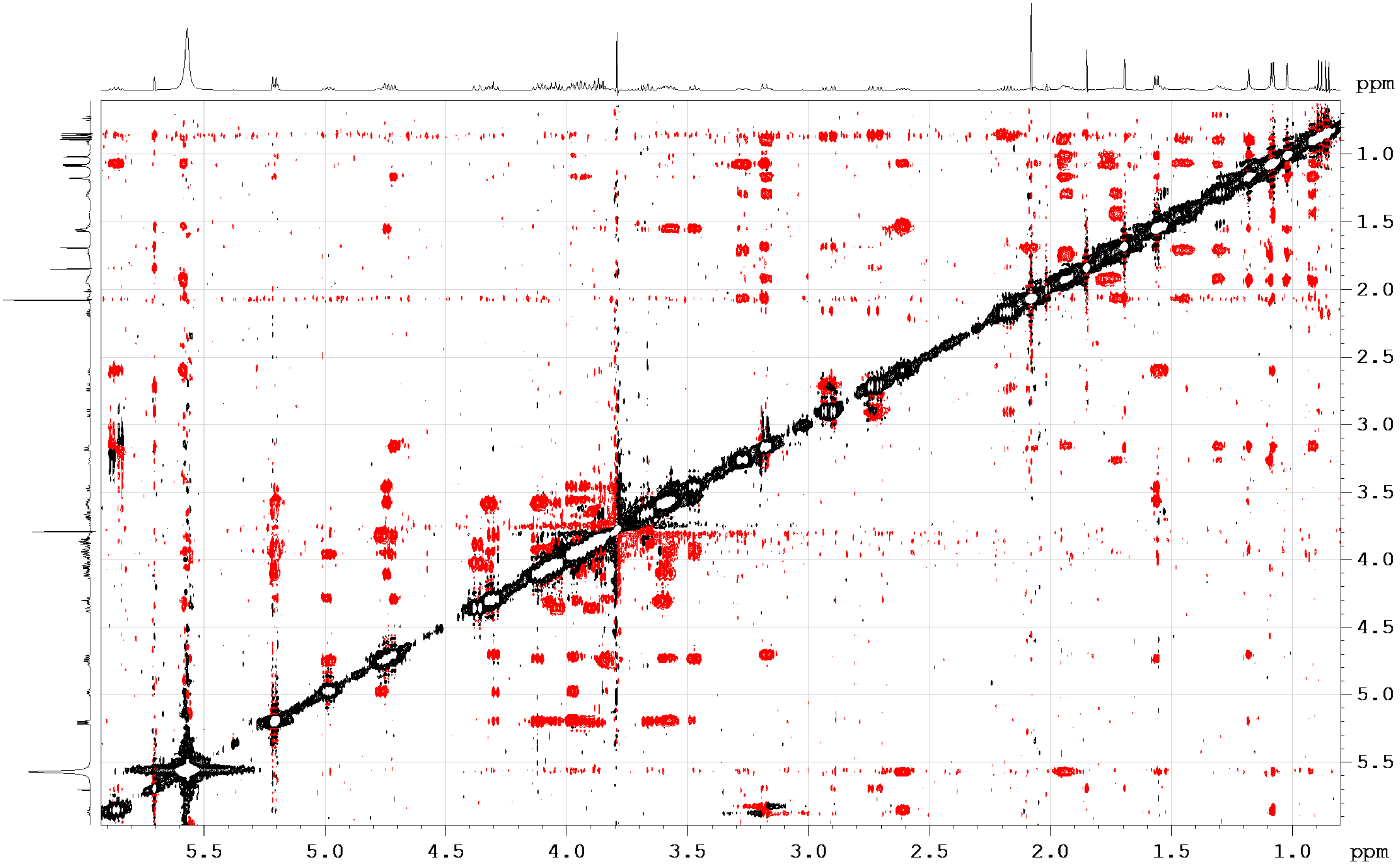

Figure S30. The ROESY (500.12 MHz) spectrum of djakonovioside B1 (**4**) in C_5D_5N/D_2O (4/1)

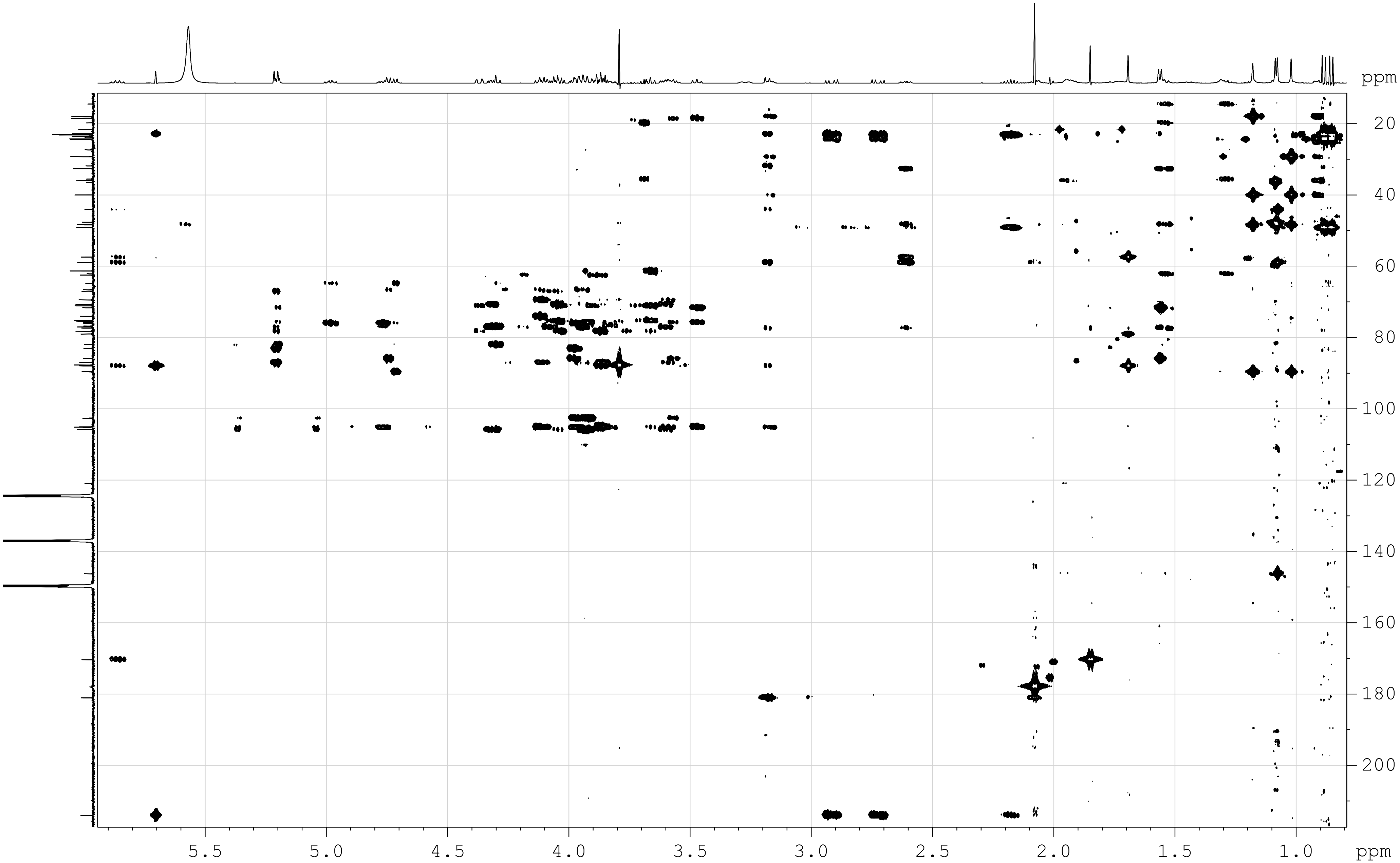

Figure S31. The HMBC (500.12 MHz) spectrum of djakonovioside B1 (**4**) in C_5D_5N/D_2O (4/1)

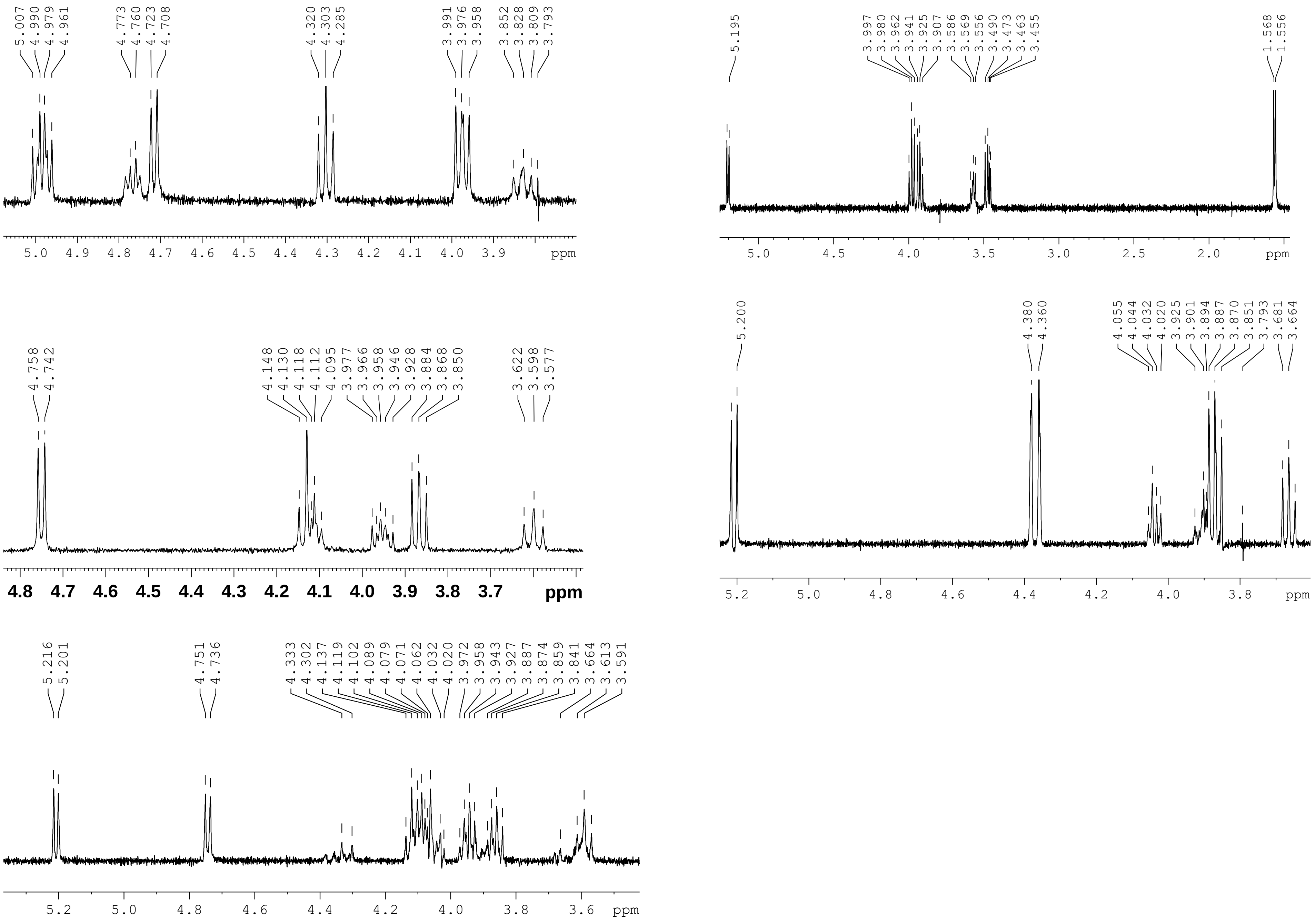

Figure S32. 1D TOCSY (500.12 MHz) spectra of Xyl1, Qui2, Xyl3, MeGlc4, Xyl5 of djakonovioside B$_1$ (**4**) in C_5D_5N/D_2O (4/1)

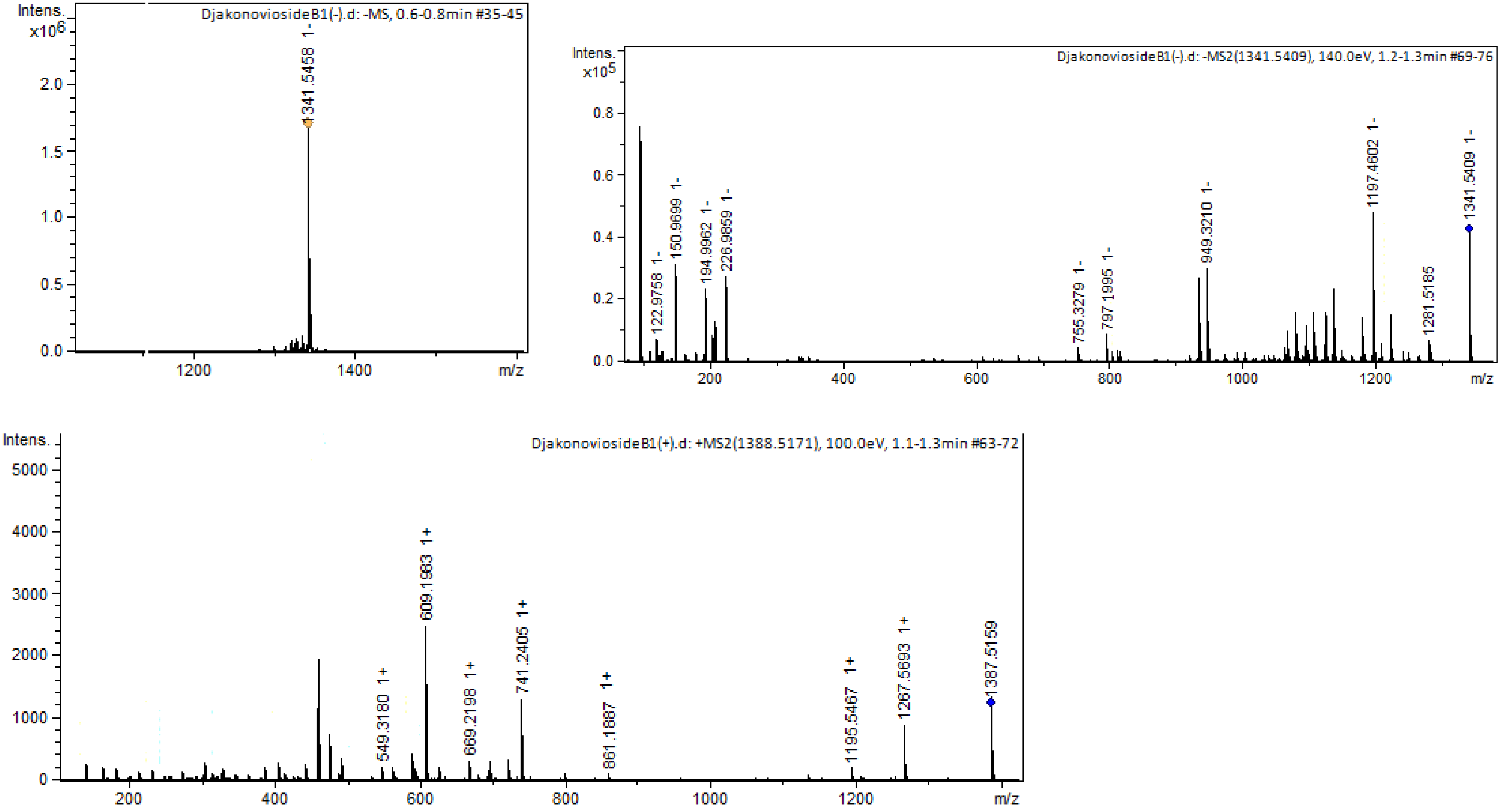

Figure S33. HR-ESI-MS and ESI-MS/MS spectra of djakonovioside B1 (**4**)

Table S3. ^{13}C and ^{1}H NMR chemical shifts, HMBC and ROESY correlations of the aglycone part of djakonovioside B_1 (**4**). [a] Recorded at 176.04 MHz in C_5D_5N. [b] Recorded at 500.13 MHz in C_5D_5N/D_2O. Multiplicity by 1D TOCSY

Position	δ_C mult.[a]	δ_H mult. (*J* in Hz)[b]	HMBC	ROESY
1	36.5 CH_2	1.29 m		H-3, H-19
2	27.4 CH_2	1.92 m		H-19, H-30
		1.73 m		
3	89.6 CH	3.17 dd (4.1; 11.4)	C: 4, 30, 31, C: 1 Xyl1	H-1, H-5, H-31, H1-Xyl1
4	40.0 C			
5	48.4 CH	0.92 dd (4.2; 7.8)	C: 4, 10, 19, 30, 31	H-3, H-31
6	23.8 CH_2	1.94 m		H-31
7	121.0 CH	5.60 m	C: 9	H-15, H-32
8	146.3 C			
9	47.7 CH	3.27 brd (14.9)		H-19
10	36.0 C			
11	23.2 CH_2	1.73 m		
		1.46 m		
12	31.9 CH_2	2.08 m	C: 13, 18	H-21
13	59.0 C			
14	48.3 C			
15	44.1 CH_2	2.61 dd (7.3; 12.2)	C: 13, 14, 16, 17, 32	H-7, H-32
		1.55 d (8.4)	C: 14, 16, 32	
16	77.4 CH	5.86 q (8.6)	C: 13, 15, 17, 20, OAc	H-32
17	57.5 CH	3.18 d (8.9)	C: 12, 13, 15, 16, 18,	H-12, H-21, H-32
18	181.1 C			
19	24.5 CH_3	1.09 s	C: 1, 9, 10	H-1, H-2, H-9, H-11
20	88.0 C			
21	22.9 CH_3	1.69 s	C: 17, 20, 22	H-12, H-17, H-22
22	79.0 CH	5.70 s	C: 17, 20, 21, 23	H-17, H-21, H-24, H-26, OAc
23	214.0 C			
24	49.2 CH_2	2.92 dd (6.5; 17.8)	C: 23, 25, 26, 27	
		2.72 dd (7.0; 17.8)	C: 23, 25, 26, 27	H-22
25	24.3 CH	2.18 quintet (6.5)	C: 23, 24, 26, 27	
26	23.1 CH_3	0.85 d (6.5)	C: 24, 27	H-25
27	23.2 CH_3	0.89 d (6.5)	C: 24, 26	H-25
30	18.0 CH_3	1.02 s	C: 3, 4, 5, 31	H-2, H-6, H-31
31	29.3 CH_3	1.18 s	C: 3, 4, 5, 30	H-3, H-5, H-6, H-30, H-1 Xyl1
32	32.7 CH_3	1.08 s	C: 8, 13, 14, 15	H-7, H-12, H-15, H-16, H-17
O<u>C</u>OCH$_3$	170.4 C			
OCO<u>C</u>H$_3$	21.7 CH_3	1.85 s	C: 16, OAc	H-22, H-24, H-26

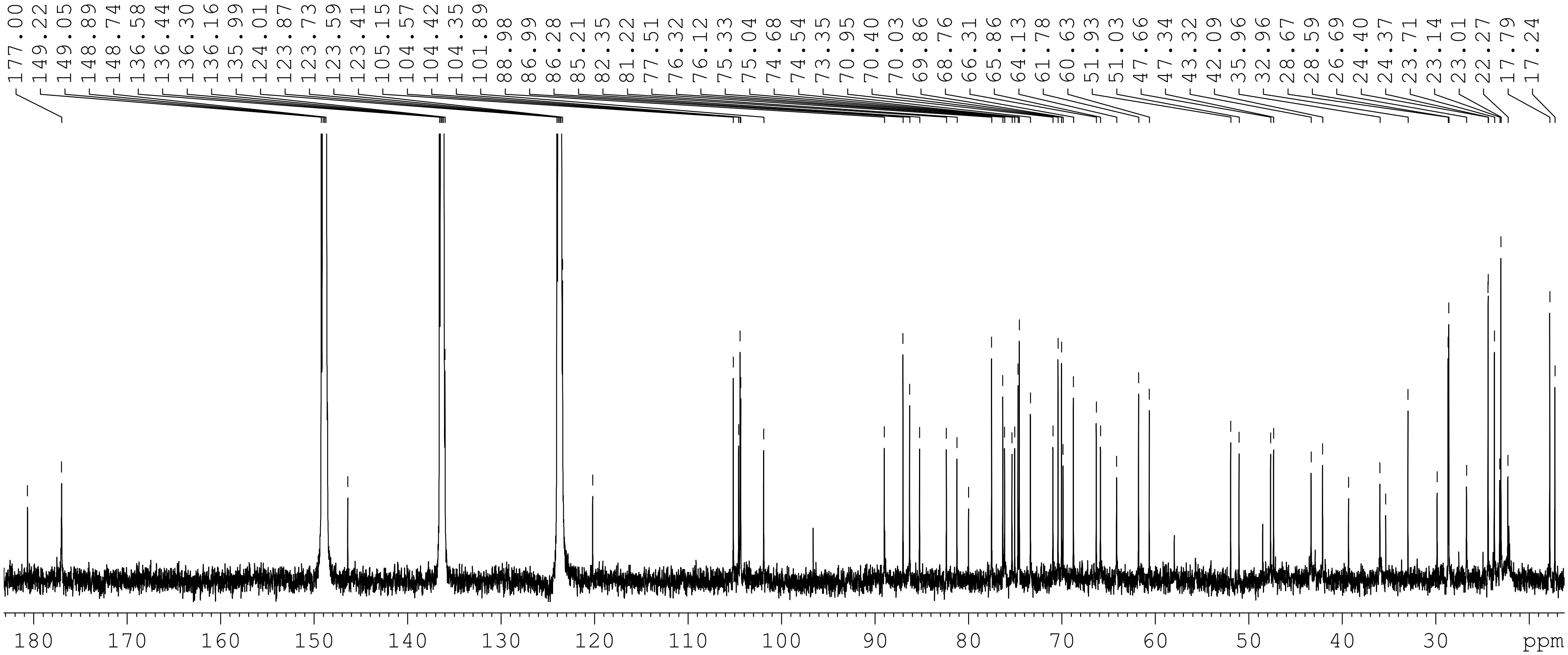

Figure S34. The ^{13}C NMR (125.67 MHz) spectrum of djakonovioside B$_2$ (**5**) in C_5D_5N/D_2O (4/1)

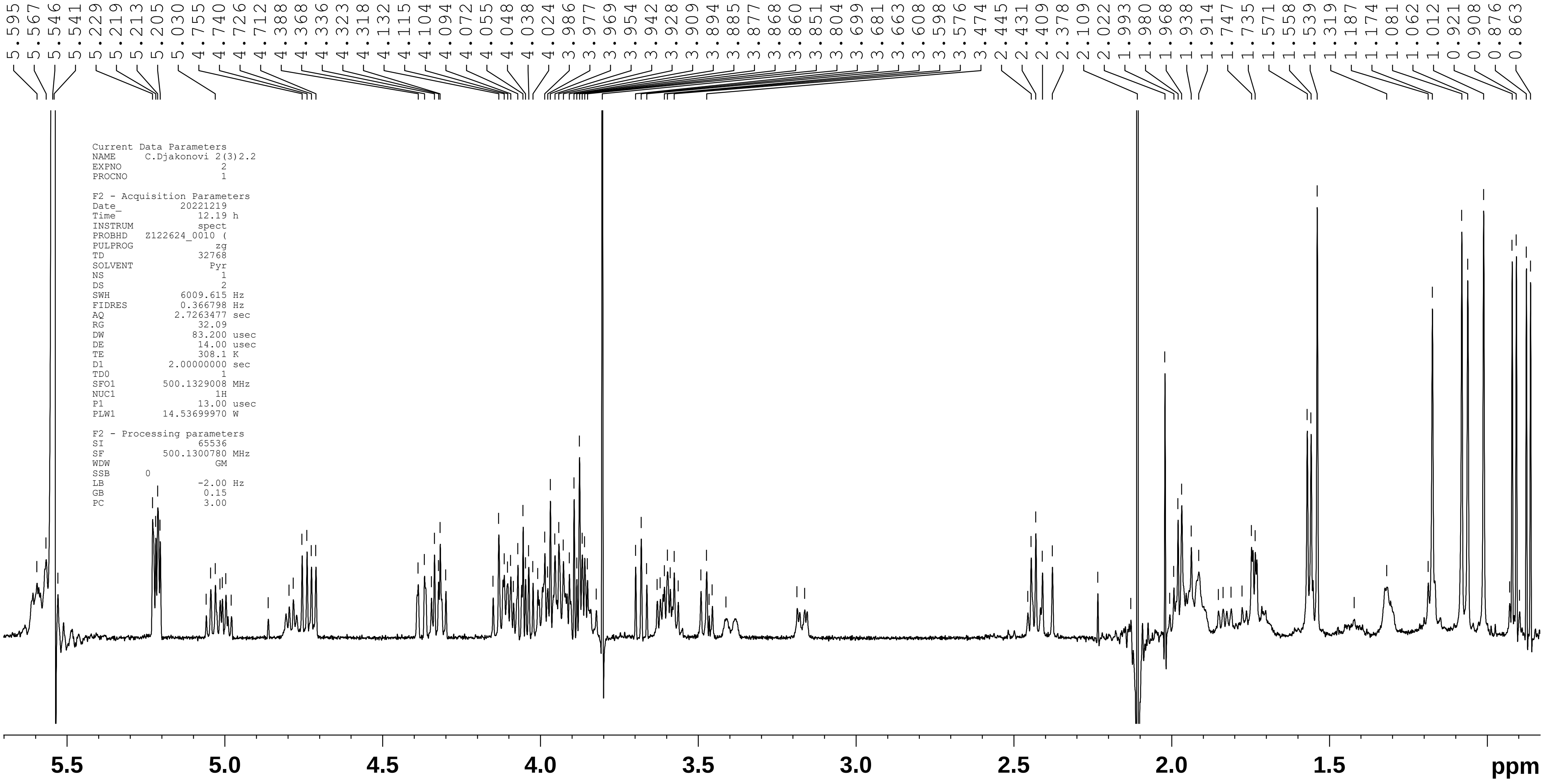

Figure S35. The ¹H NMR (500.12 MHz) spectrum of djakonovioside B₂ (**5**) in C₅D₅N/D₂O (4/1)

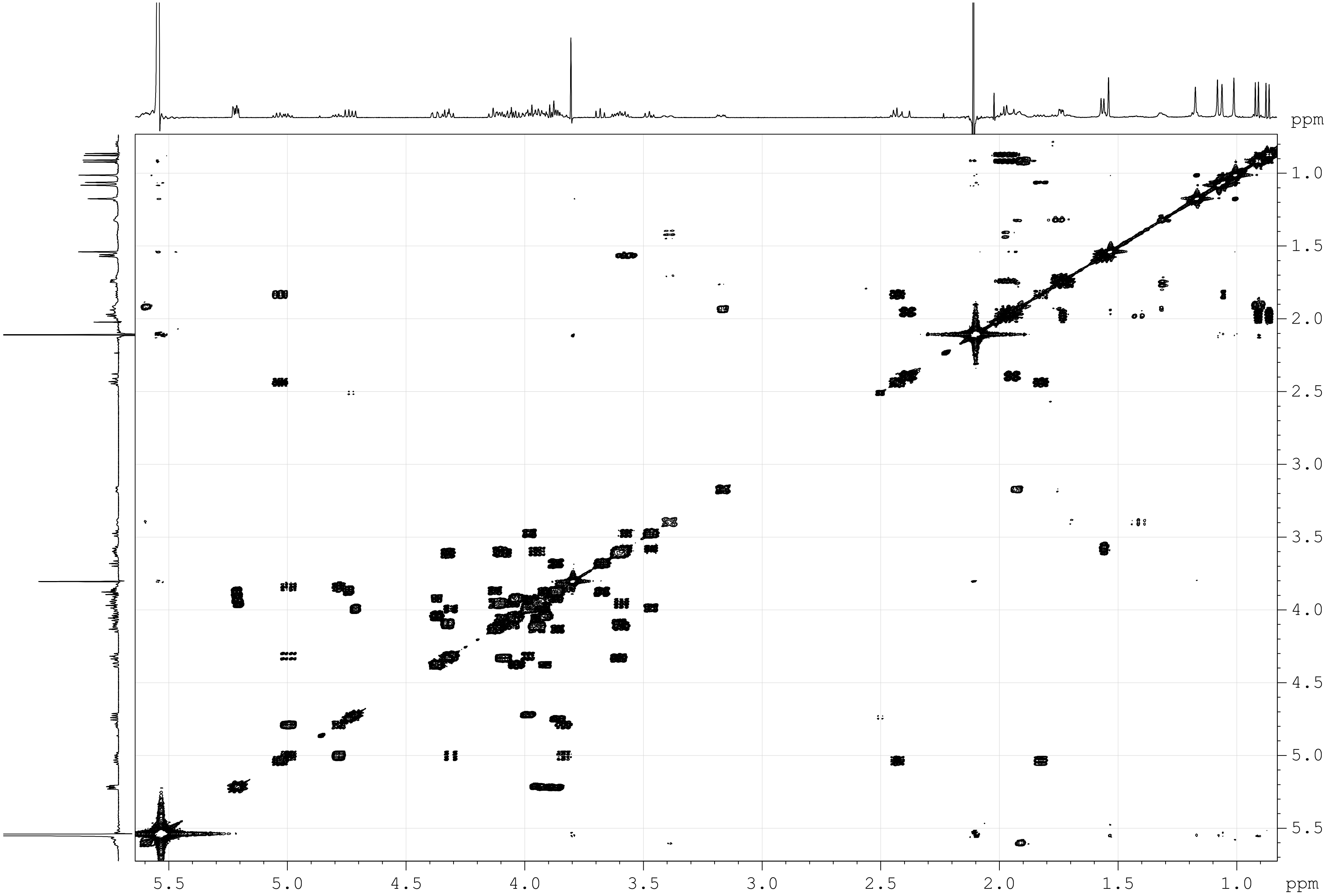

Figure S36. The COSY (500.12 MHz) spectrum of djakonovioside B_2 (**5**) in C_5D_5N/D_2O (4/1)

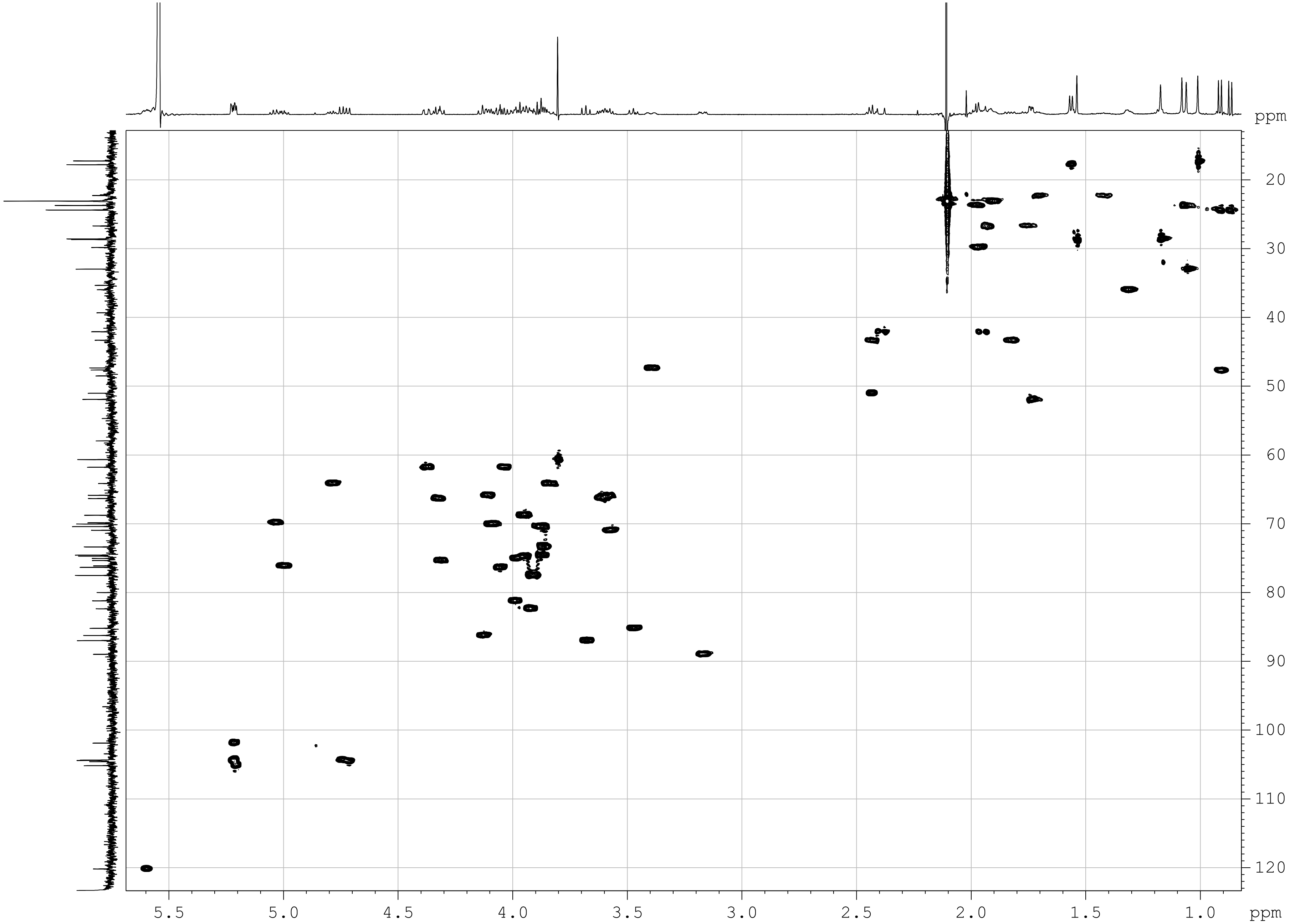

Figure S37. The HSQC (500.12 MHz) spectrum of djakonovioside B_2 (**5**) in C_5D_5N/D_2O (4/1)

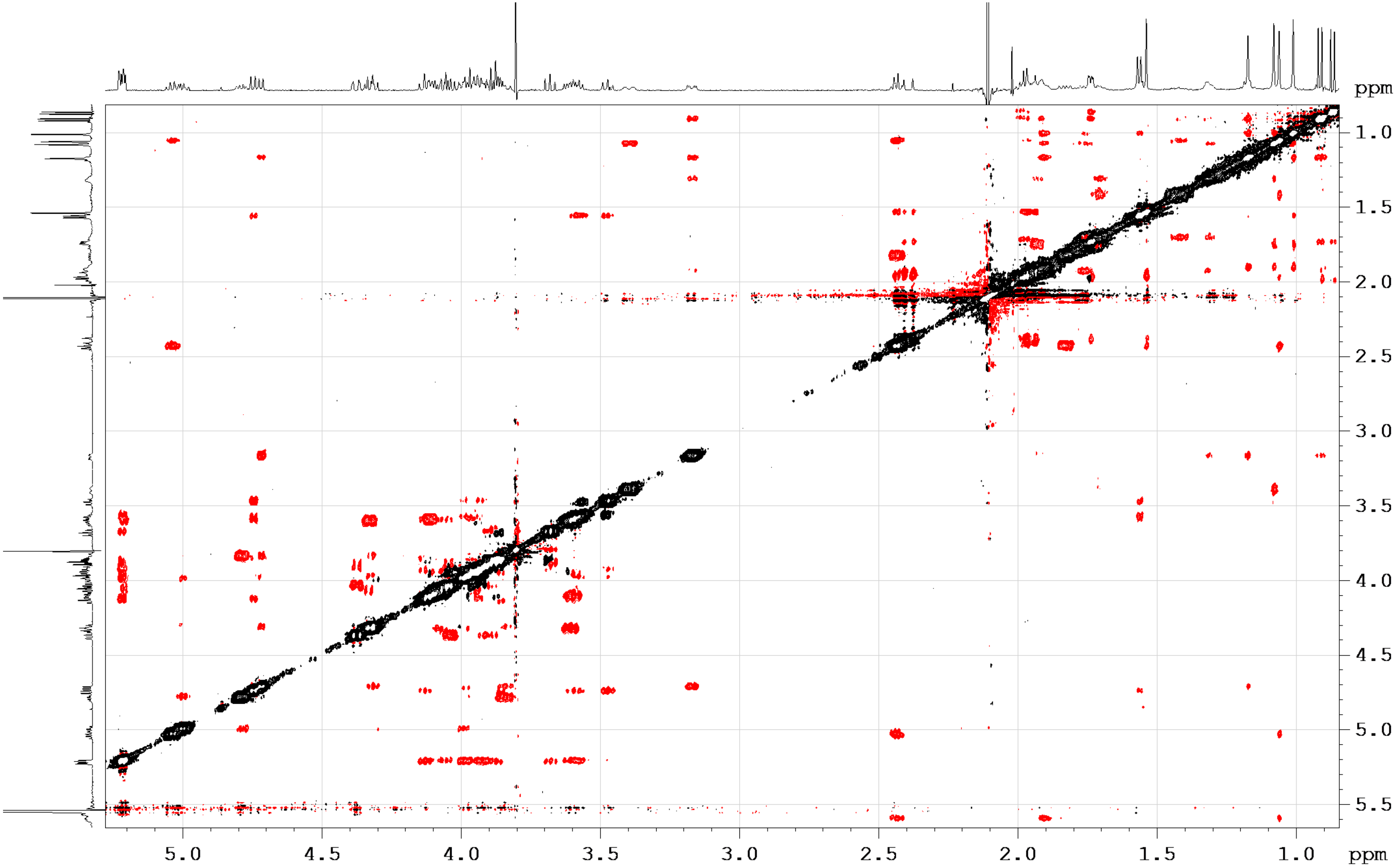

Figure S38. The ROESY (500.12 MHz) spectrum of djakonovioside B2 (**5**) in C_5D_5N/D_2O (4/1)

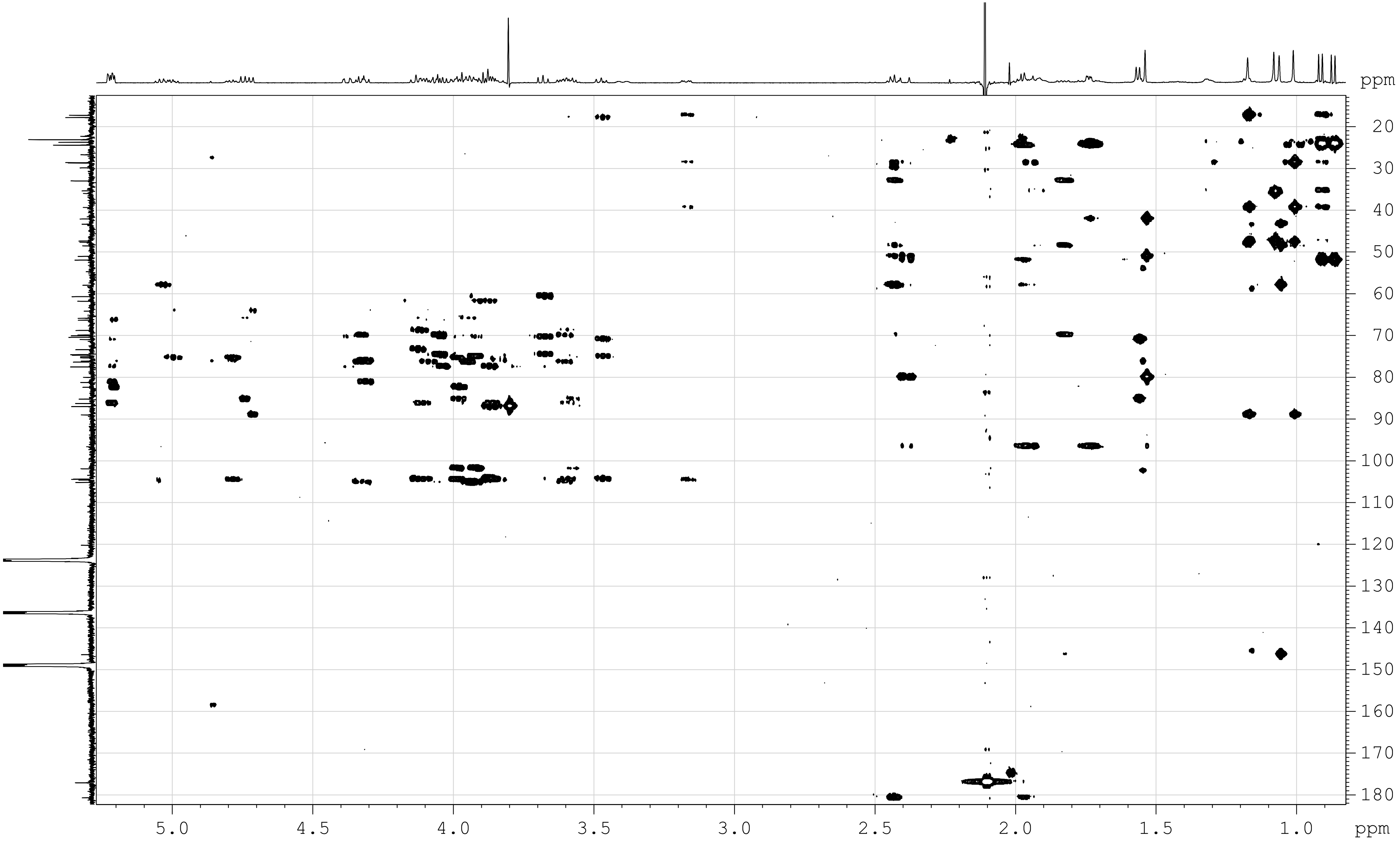

Figure S39. The HMBC (500.12 MHz) spectrum of djakonovioside B_2 (**5**) in C_5D_5N/D_2O (4/1)

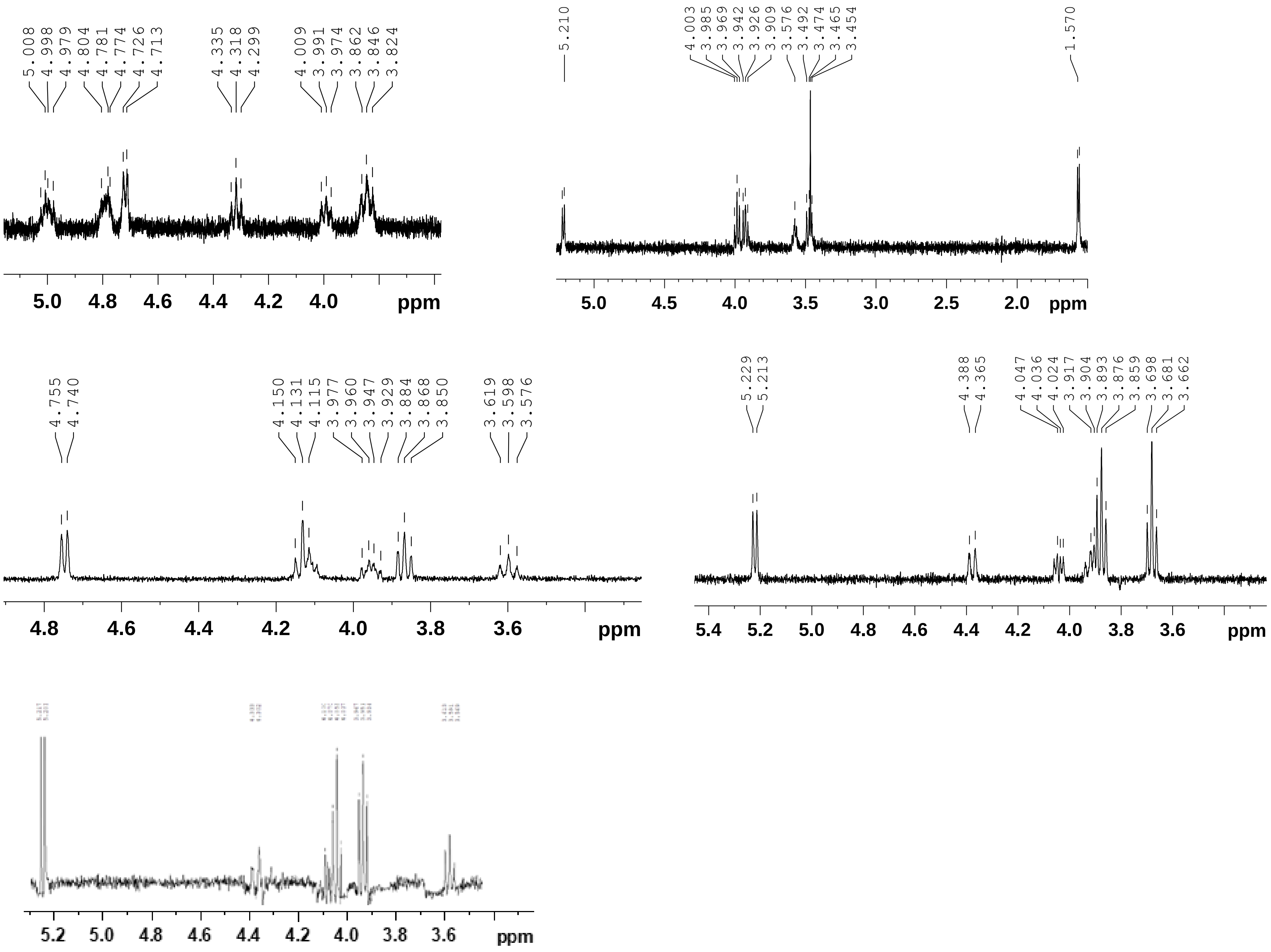

Figure S40. 1D TOCSY (500.12 MHz) spectra of Xyl1, Qui2, Xyl3, MeGlc4, Xyl5 of djakonovioside B$_2$ (**5**) in C_5D_5N/D_2O (4/1)

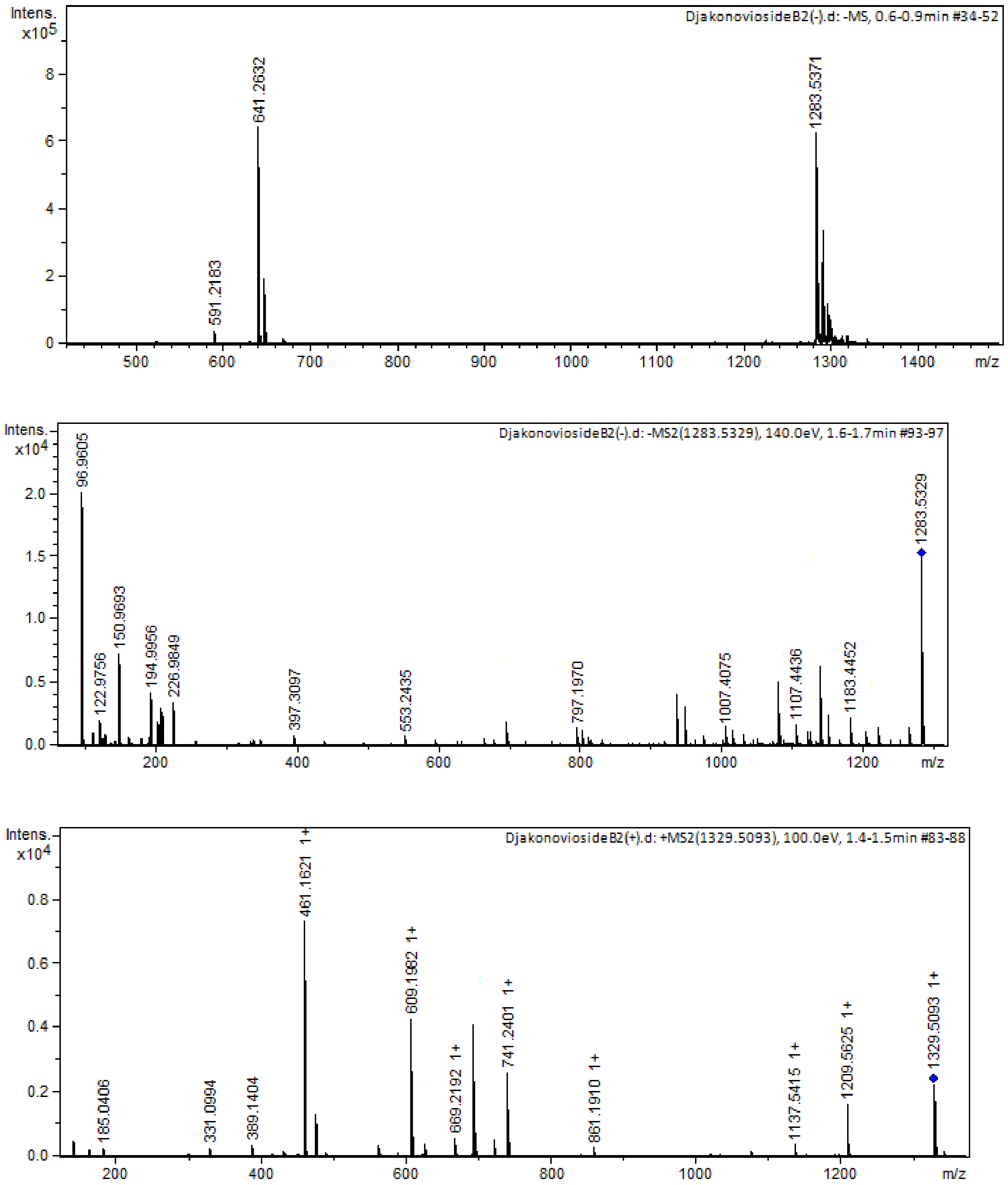

Figure S41. HR-ESI-MS and ESI-MS/MS spectra of djakonovioside B_2 (**5**)

Table S4. ^{13}C and ^{1}H NMR chemical shifts and HMBC and ROESY correlations of carbohydrate moiety of **djakonovioside B$_2$**. [a] Recorded at 176.04 MHz in C_5D_5N. [b] Recorded at 500.13 MHz in C_5D_5N/D_2O. Multiplicity by 1D TOCSY. [c] Bold = interglycosidic positions, [d] Italic – sulfate positions

Atom	δC mult.[a,c,d]	δH mult. (*J* in Hz)[b]	HMBC	ROESY
Xyl1 (1→C-3)				
1	104.6 CH	4.72 d (6.2)	C: 3; C: 5 Xyl1	H-3; H-3, 5 Xyl1
2	**81.2** CH	3.99 t (8.8)	C: 1 Qui2; C: 1, 3 Xyl1	H-1 Qui2; H-4 Xyl1
3	75.3 CH	4.32 t (8.8)	C: 2, 4 Xyl1	H-1, 5 Xyl1
4	*76.1* CH	5.00 m	C: 3 Xyl1	H-2 Xyl1
5	64.1 CH_2	4.78 m	C: 1, 3 Xyl1	
		3.84 m		H-1, 3 Xyl1
Qui2 (1→2Xyl1)				
1	104.4 CH	5.22 d (7.2)	C: 2 Xyl1	H-2 Xyl1; H-3, 5 Qui2
2	**82.4** CH	3.93 t (8.0)	C: 3 Qui2; C: 1 Xyl5	H-4 Qui2
3	75.1 CH	3.98 t (8.0)	C: 2, 4 Qui2	H-1, 5 Qui2
4	**85.2** CH	3.47 t (8.0)	C: 3, 5, 6 Qui2; C: 1 Xyl3	H-1 Xyl3; H-2 Qui2
5	71.0 CH	3.57 dd (6.3; 8.0)	C: 4 Qui2	H-1 Qui2
6	17.8 CH_3	1.56 d (6.3)	C: 4, 5 Qui2	
Xyl3 (1→4Qui2)				
1	104.4 CH	4.75 d (7.5)	C: 4 Qui2	H-4 Qui2; H-3, 5 Xyl3
2	73.4 CH	3.87 t (8.6)	C: 1 Xyl3	
3	**86.3** CH	4.13 t (8.6)	C: 2, 4 Xyl3	H-1 MeGlc4; H-1 Xyl3
4	68.8 CH	3.96 m		
5	65.9 CH_2	4.11 dd (5.4; 10.2)	C: 1, 3 Xyl3	
		3.60 t (10.2)	C: 1, 4 Xyl3	H-1, 3 Xyl3
MeGlc4 (1→3Xyl3)				
1	105.2 CH	5.22 d (7.2)	C: 3 Xyl3	H-3 Xyl3; H-3, 5 MeGlc4
2	74.5 CH	3.87 t (8.7)		
3	87.0 CH	3.68 t (8.7)	C: 2, 4 MeGlc4; OMe	H-1, 5 MeGlc4
4	70.4 CH	3.87 t (8.7)	C: 3, 5, 6 MeGlc4	
5	77.5 CH	3.92 m	C: 6 MeGlc4	H-1 MeGlc4
6	61.8 CH_2	4.38 brd (11.6)		
		4.04 dd (5.8; 11.6)	C: 5 MeGlc4	
OMe	60.7 CH_3	3.81 s	C: 3 MeGlc4	
Xyl5 (1→2Qui2)				
1	101.9 CH	5.21 d (8.0)	C: 2 Qui2; X: 5Xyl5	H-2 Qui2
2	74.7 CH	3.95 t (8.0)	C: 3 Xyl5	
3	76.3 CH	4.05 t (8.0)	C: 2, 4 Xyl5	H-1, 5 Xyl5
4	70.0 CH	4.09 m	C: 3 Xyl5	
5	66.3 CH_2	4.33 d (4.9; 12.0)	C: 3, 4 Xyl5	
		3.61 t (10.8)	C: 3, 4 Xyl5	H-1 Xyl5

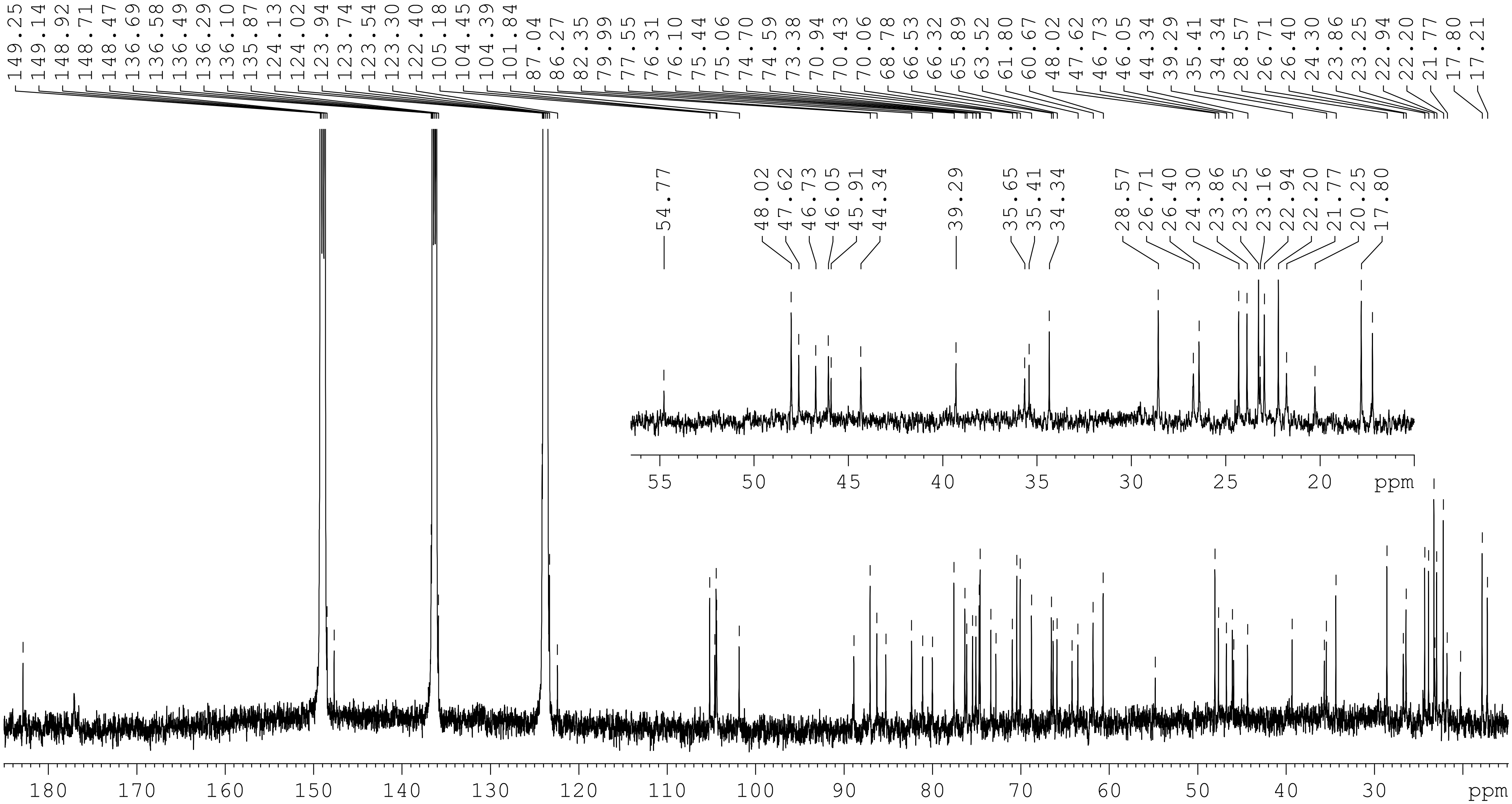

Figure S42. The ^{13}C NMR (125.67 MHz) spectrum of djakonovioside B_3 (**6**) in C_5D_5N/D_2O (4/1)

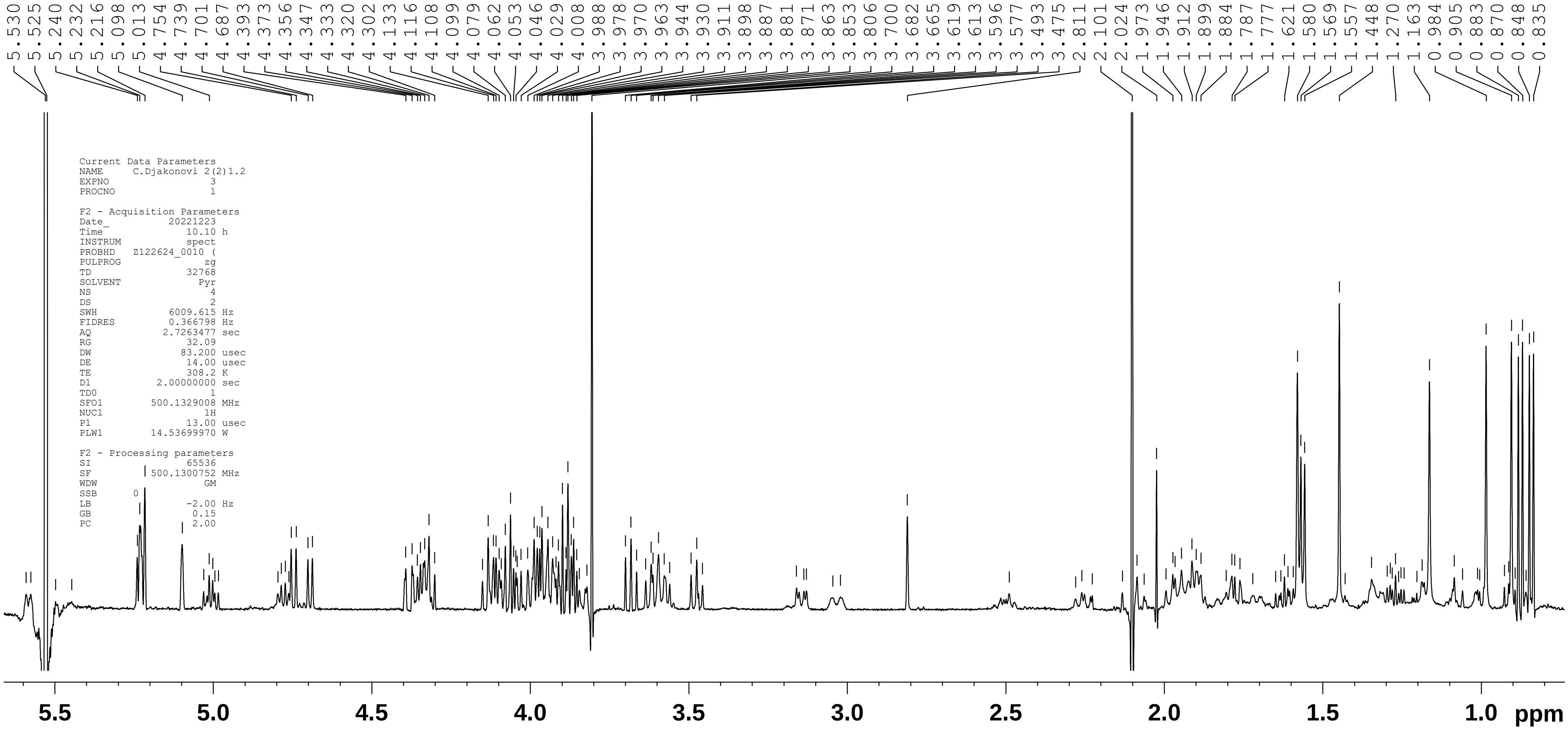

Figure S43. The ^{1}H NMR (500.12 MHz) spectrum of djakonovioside B$_3$ (**6**) in C_5D_5N/D_2O (4/1)

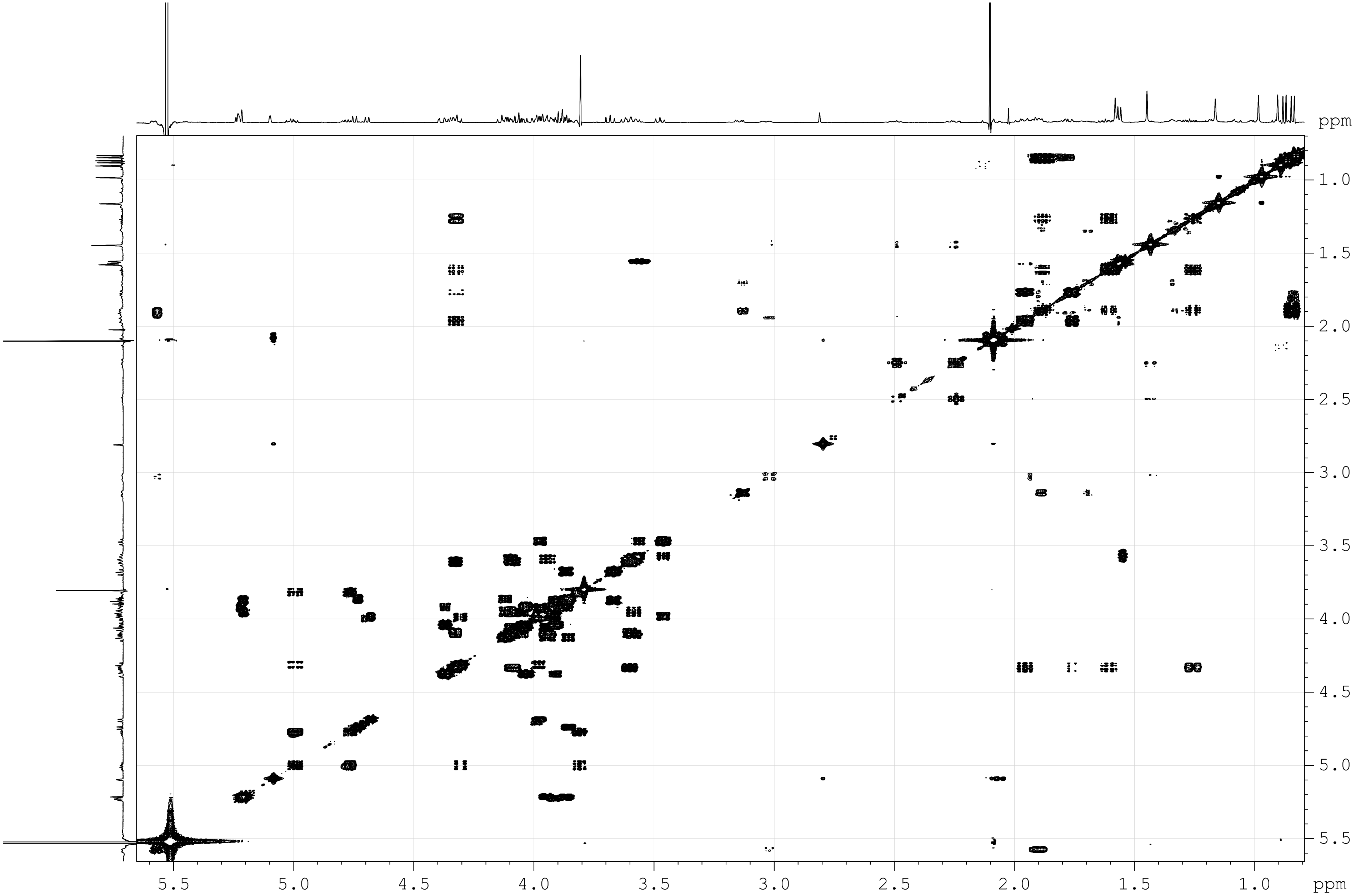

Figure S44. The COSY (500.12 MHz) spectrum of djakonovioside B_3 (**6**) in C_5D_5N/D_2O (4/1)

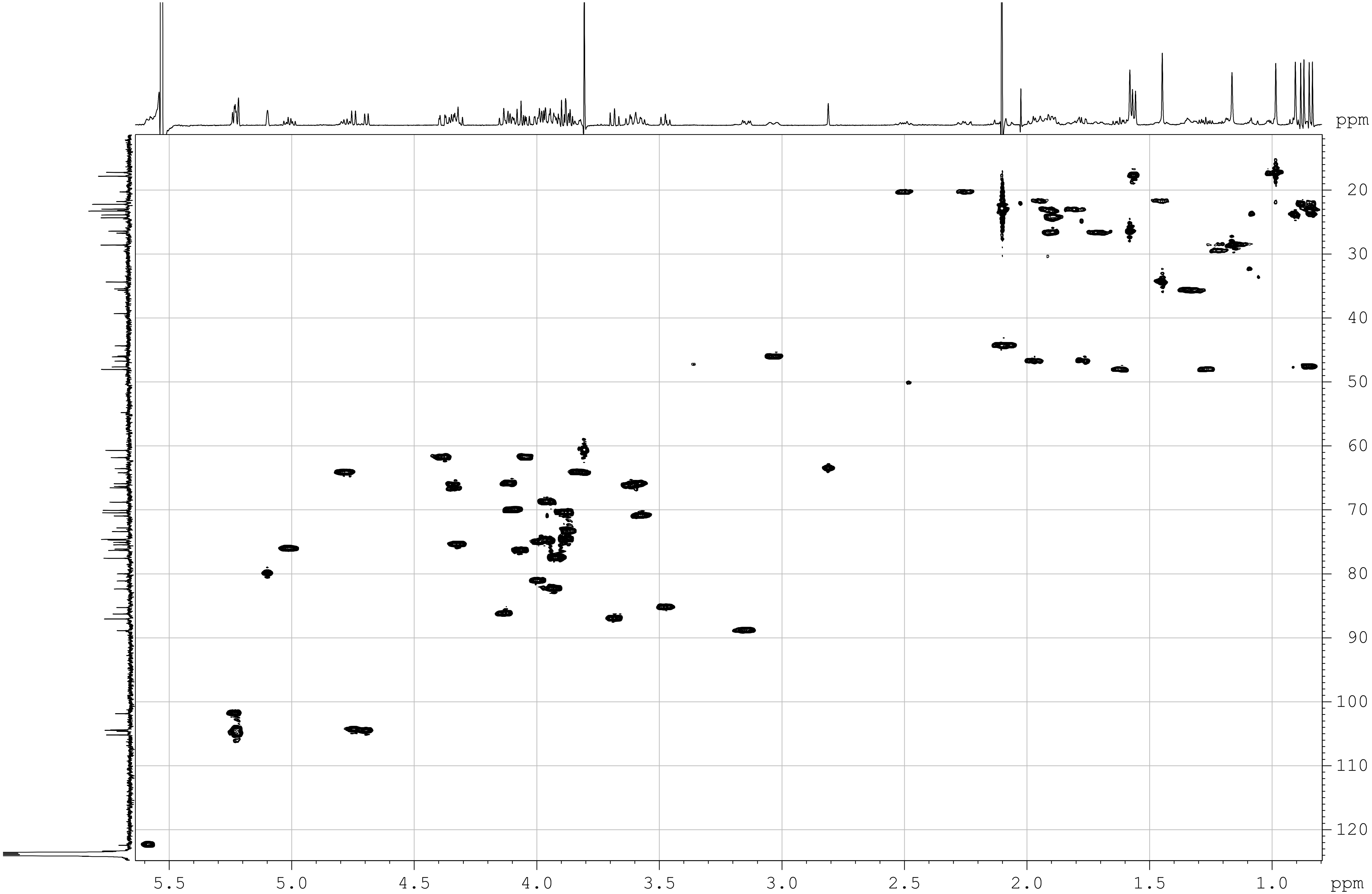

Figure S45. The HSQC (500.12 MHz) spectrum of djakonovioside B_3 (**6**) in C_5D_5N/D_2O (4/1)

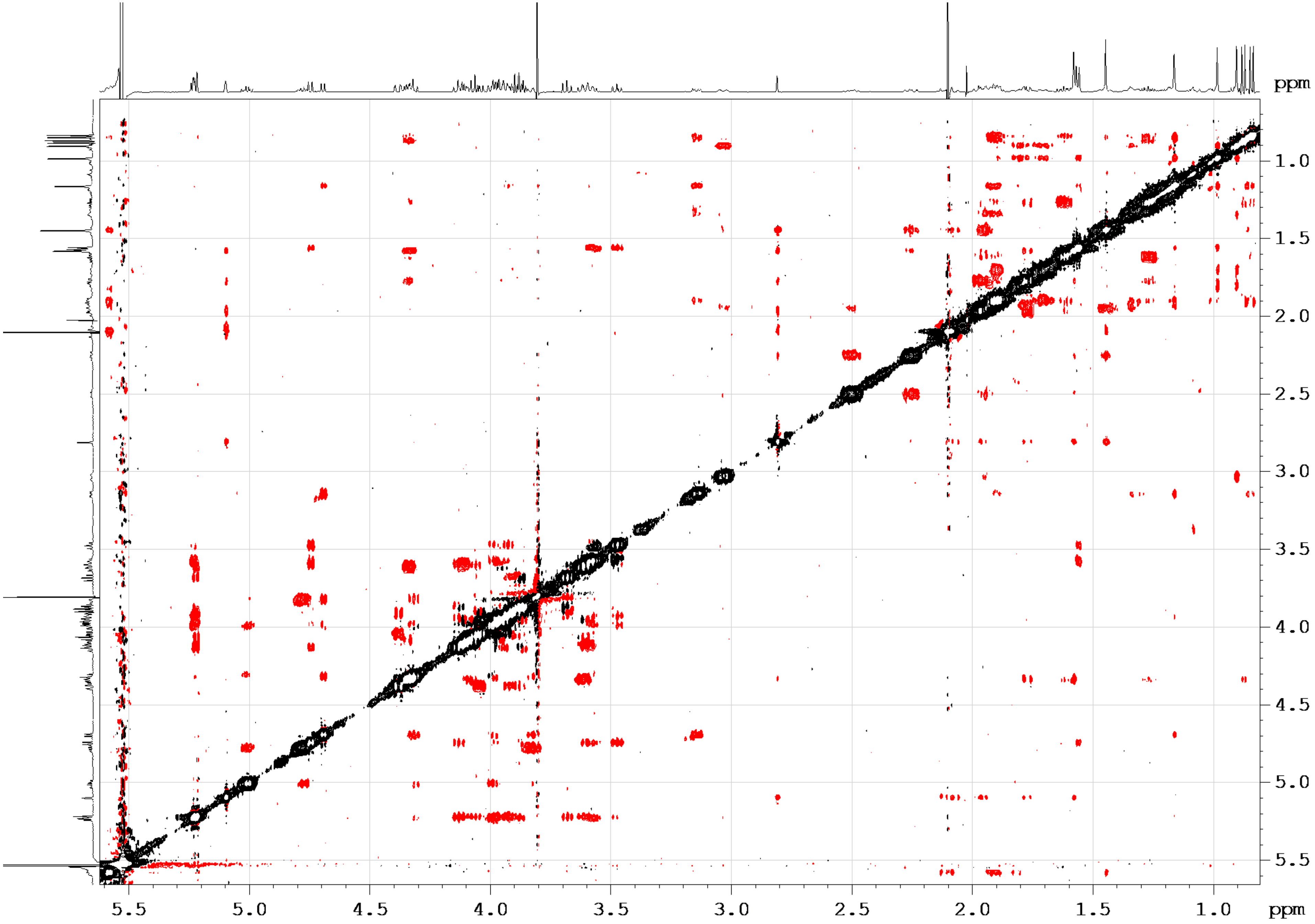

Figure S46. The ROESY (500.12 MHz) spectrum of djakonovioside B$_3$ (**6**) in C_5D_5N/D_2O (4/1)

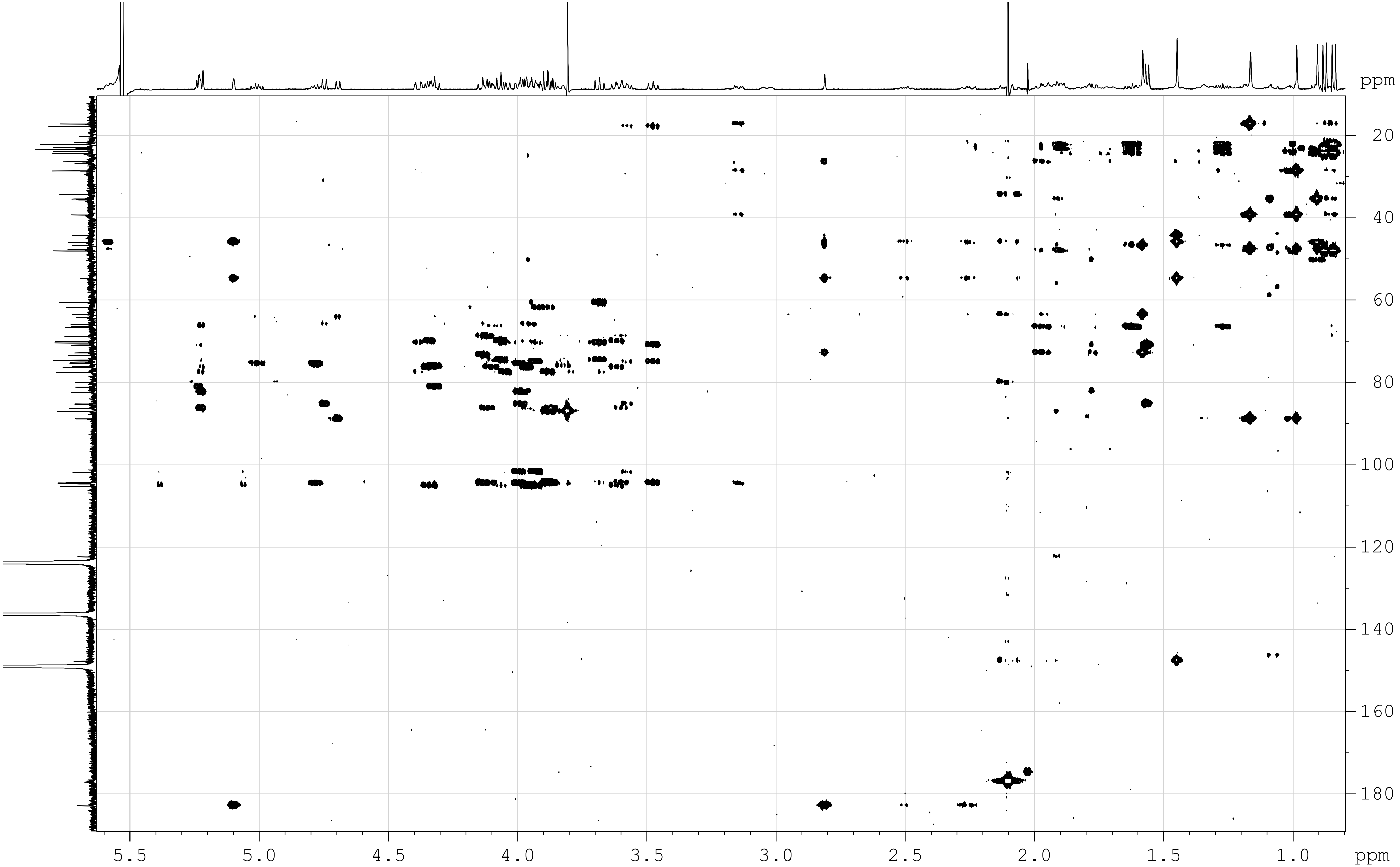

Figure S47. The HMBC (500.12 MHz) spectrum of djakonovioside B$_3$ (**6**) in C_5D_5N/D_2O (4/1)

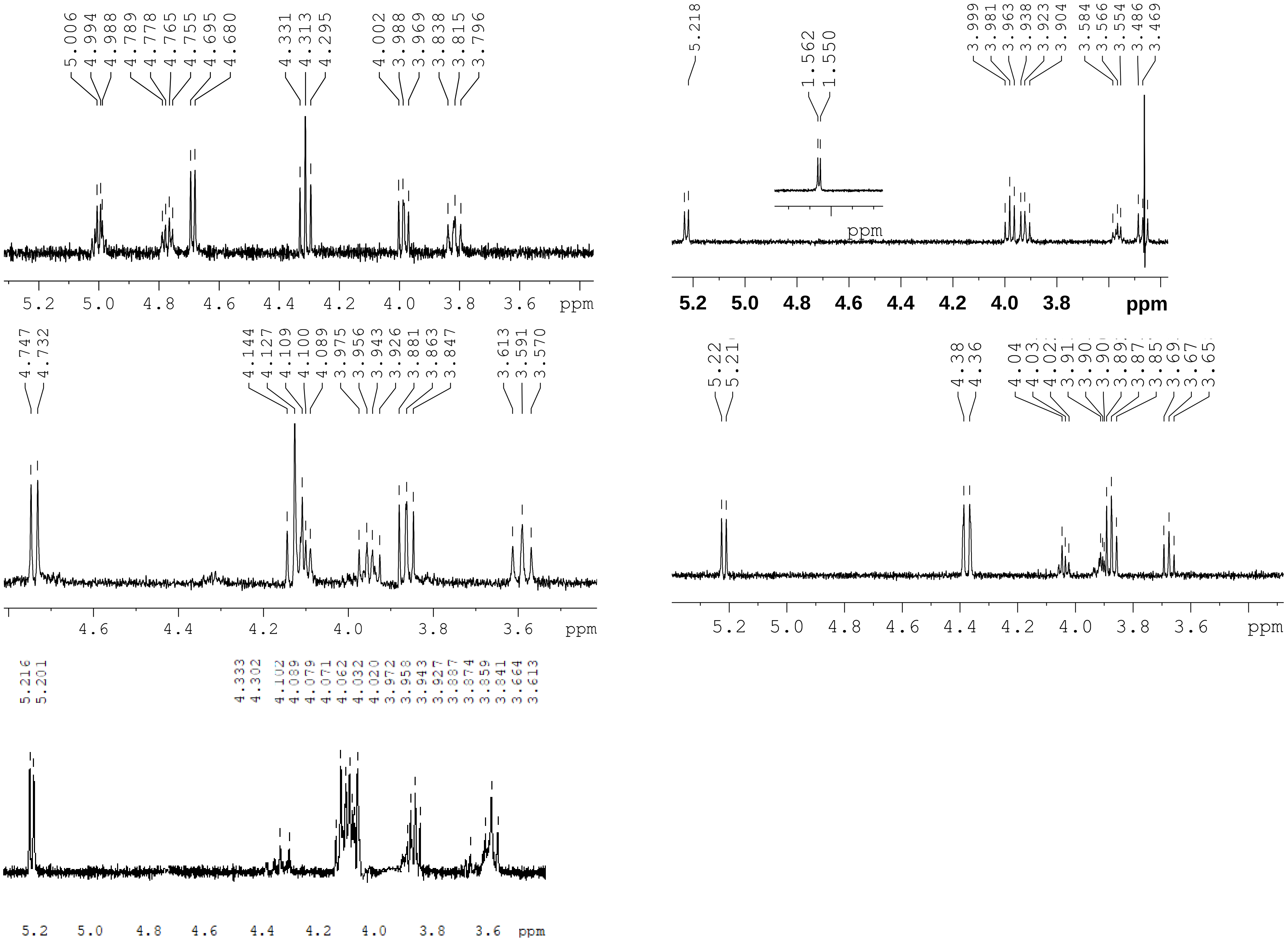

Figure S48. 1D TOCSY (500.12 MHz) spectra of Xyl1, Qui2, Xyl3, MeGlc4, Xyl5 of djakonovioside B$_3$ (**6**) in C_5D_5N/D_2O (4/1)

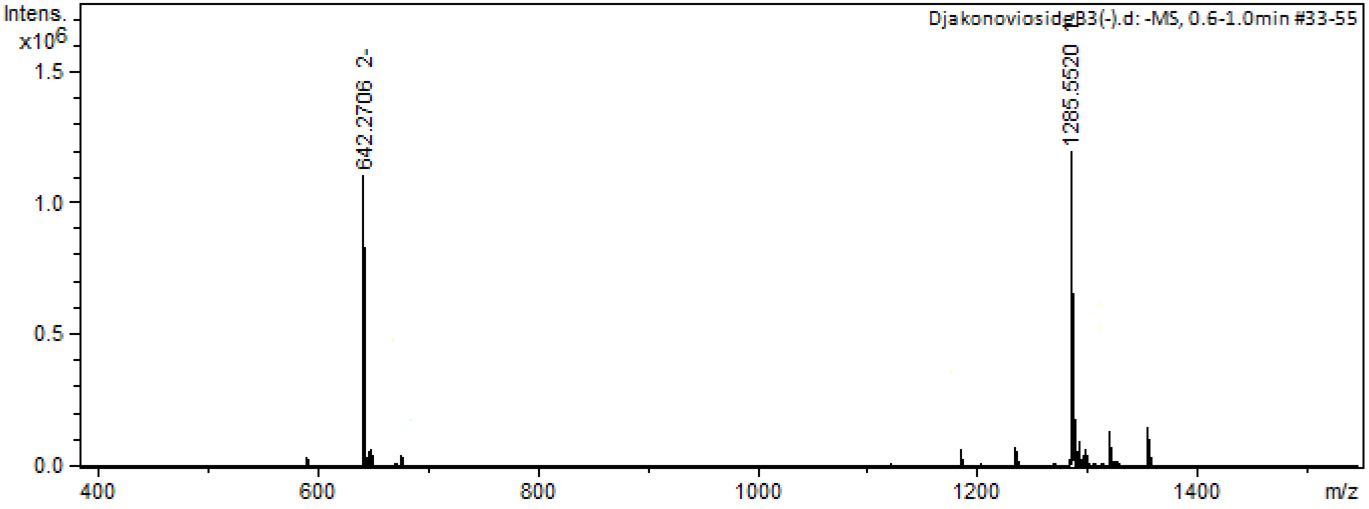

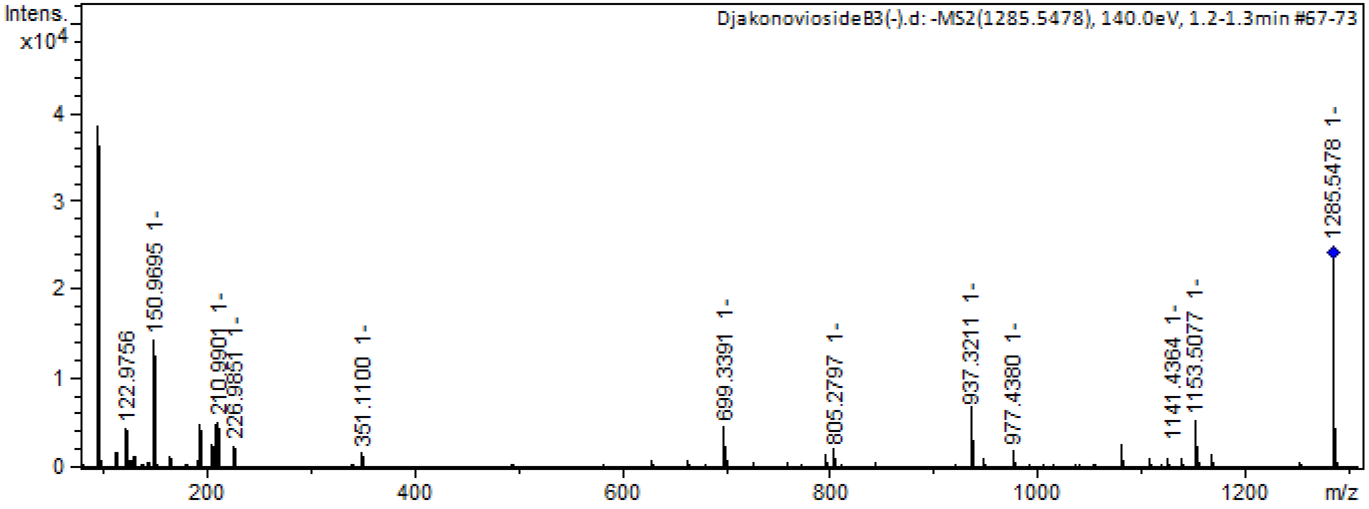

Figure S49. HR-ESI-MS and ESI-MS/MS spectra of djakonovioside B_3 (**6**)

Table S5. ^{13}C and ^{1}H NMR chemical shifts and HMBC and ROESY correlations of carbohydrate moiety of **djakonovioside B$_3$**. [a] Recorded at 176.04 MHz in C_5D_5N. [b] Recorded at 500.13 MHz in C_5D_5N/D_2O. Multiplicity by 1D TOCSY. [c] Bold = interglycosidic positions, [d] Italic – sulfate positions

Atom	δC mult.[a,c,d]	δH mult. (*J* in Hz)[b]	HMBC	ROESY
Xyl1 (1→C-3)				
1	104.6 CH	4.69 d (7.7)	C: 3; C: 5 Xyl1	H-3; H-3, 5 Xyl1
2	**81.1** CH	3.99 dd (7.7; 9.6)	C: 1 Qui2; C: 1, 3 Xyl1	H-1 Qui2
3	75.4 CH	4.31 t (9.0)	C: 2, 4 Xyl1	H-1, 5 Xyl1
4	*76.1* CH	5.00 dd (3.2; 9.0)	C: 3 Xyl1	
5	64.2 CH$_2$	4.77 dd (5.1; 11.5)	C: 1, 3 Xyl1	
		3.82 dd (9.0; 11.5)		H-1, 3 Xyl1
Qui2 (1→2Xyl1)				
1	104.4 CH	5.22 d (7.1)	C: 2 Xyl1	H-2 Xyl1; H-3, 5 Qui2
2	**82.3** CH	3.92 t (8.5)	C: 3 Qui2; C: 1 Xyl5	
3	75.1 CH	3.98 t (8.5)	C: 2, 4 Qui2	H-1, 5 Qui2
4	**85.2** CH	3.47 t (8.5)	C: 3, 5, 6 Qui2; C: 1 Xyl3	H-1 Xyl3
5	70.9 CH	3.57 dd (5.7; 9.2)	C: 4 Qui2	H-1 Qui2
6	17.8 CH$_3$	1.56 d (5.7)	C: 4, 5 Qui2	
Xyl3 (1→4Qui2)				
1	104.4 CH	4.74 d (7.7)	C: 4 Qui2	H-4 Qui2; H-3, 5 Xyl3
2	73.4 CH	3.86 t (8.2)	C: 1 Xyl3	
3	**86.3** CH	4.13 t (8.2)	C: 2, 4 Xyl3	H-1 MeGlc4; H-1 Xyl3
4	68.8 CH	3.95 m		
5	65.9 CH$_2$	4.11 dd (5.5; 11.4)	C: 1, 3 Xyl3	
		3.59 t (10.9)	C: 1, 4 Xyl3	H-1, 3 Xyl3
MeGlc4 (1→3Xyl3)				
1	105.2 CH	5.22 d (8.1)	C: 3 Xyl3	H-3 Xyl3; H-3, 5 MeGlc4
2	74.5 CH	3.87 t (8.7)		
3	87.0 CH	3.68 t (8.7)	C: 2, 4 MeGlc4; OMe	H-1, 5 MeGlc4
4	70.4 CH	3.87 t (8.7)	C: 3, 5, 6 MeGlc4	
5	77.5 CH	3.91 m	C: 6 MeGlc4	H-1 MeGlc4
6	61.8 CH$_2$	4.38 brd (12.1)		
		4.04 dd (6.0; 12.1)	C: 5 MeGlc4	
OMe	60.7 CH$_3$	3.81 s	C: 3 MeGlc4	
Xyl5 (1→2Qui2)				
1	101.9 CH	5.21 d (8.0)	C: 2 Qui2; X: 5Xyl5	H-2 Qui2
2	74.7 CH	3.96 t (8.0)	C: 3 Xyl5	
3	76.3 CH	4.06 t (8.0)	C: 2, 4 Xyl5	H-1, 5 Xyl5
4	70.1 CH	4.10 m	C: 3 Xyl5	
5	66.3 CH$_2$	4.33 d (4.9; 12.0)	C: 3, 4 Xyl5	
		3.61 t (10.8)	C: 3, 4 Xyl5	H-1 Xyl5

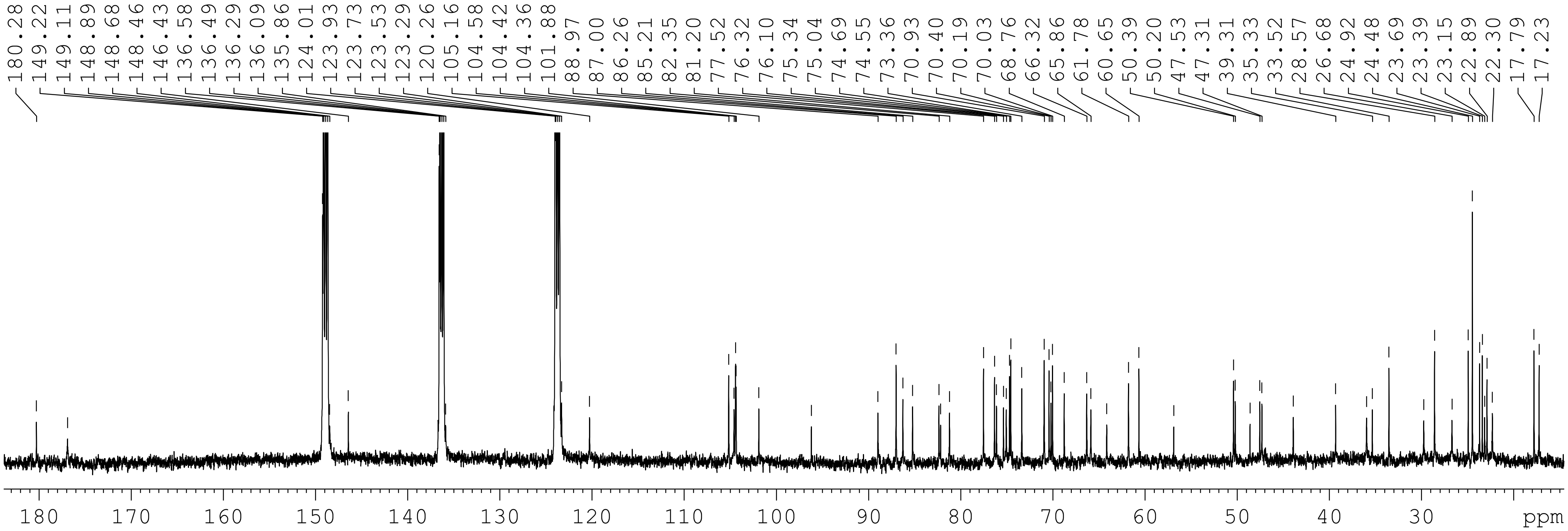

Figure S50. The ^{13}C NMR (125.67 MHz) spectrum of djakonovioside B4 (**7**) in C_5D_5N/D_2O (4/1)

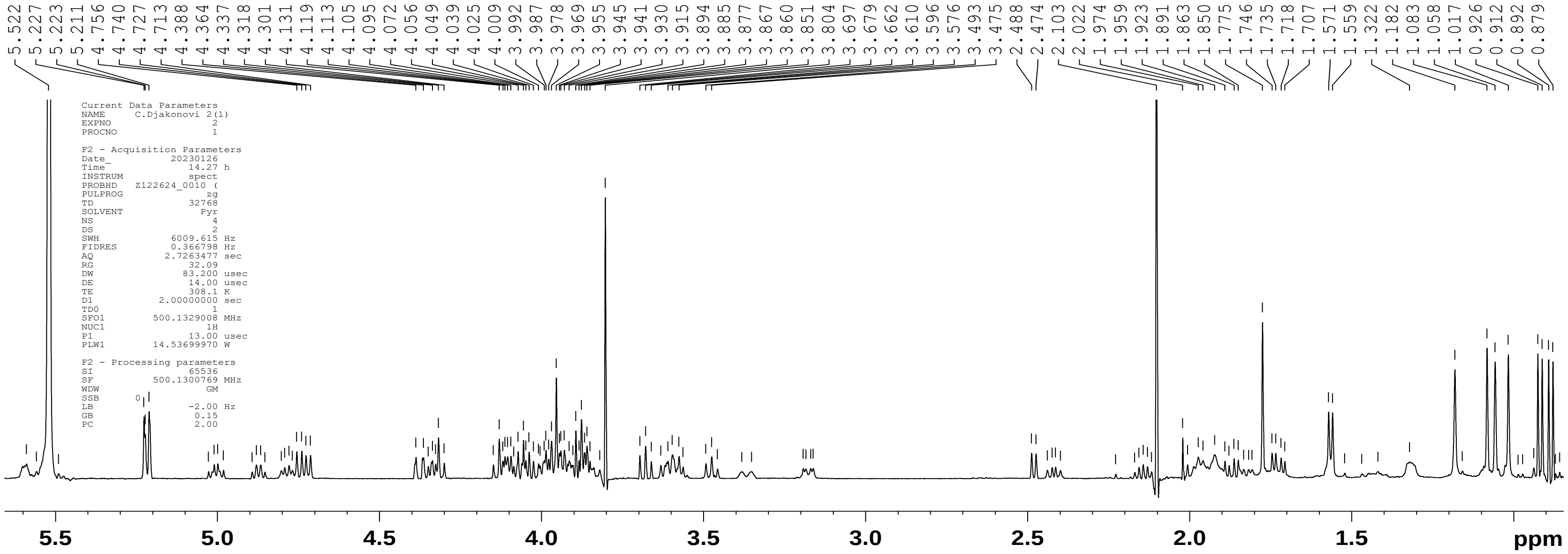

Figure S51. The ^{1}H NMR (500.12 MHz) spectrum of djakonovioside B4 (**7**) in C_5D_5N/D_2O (4/1)

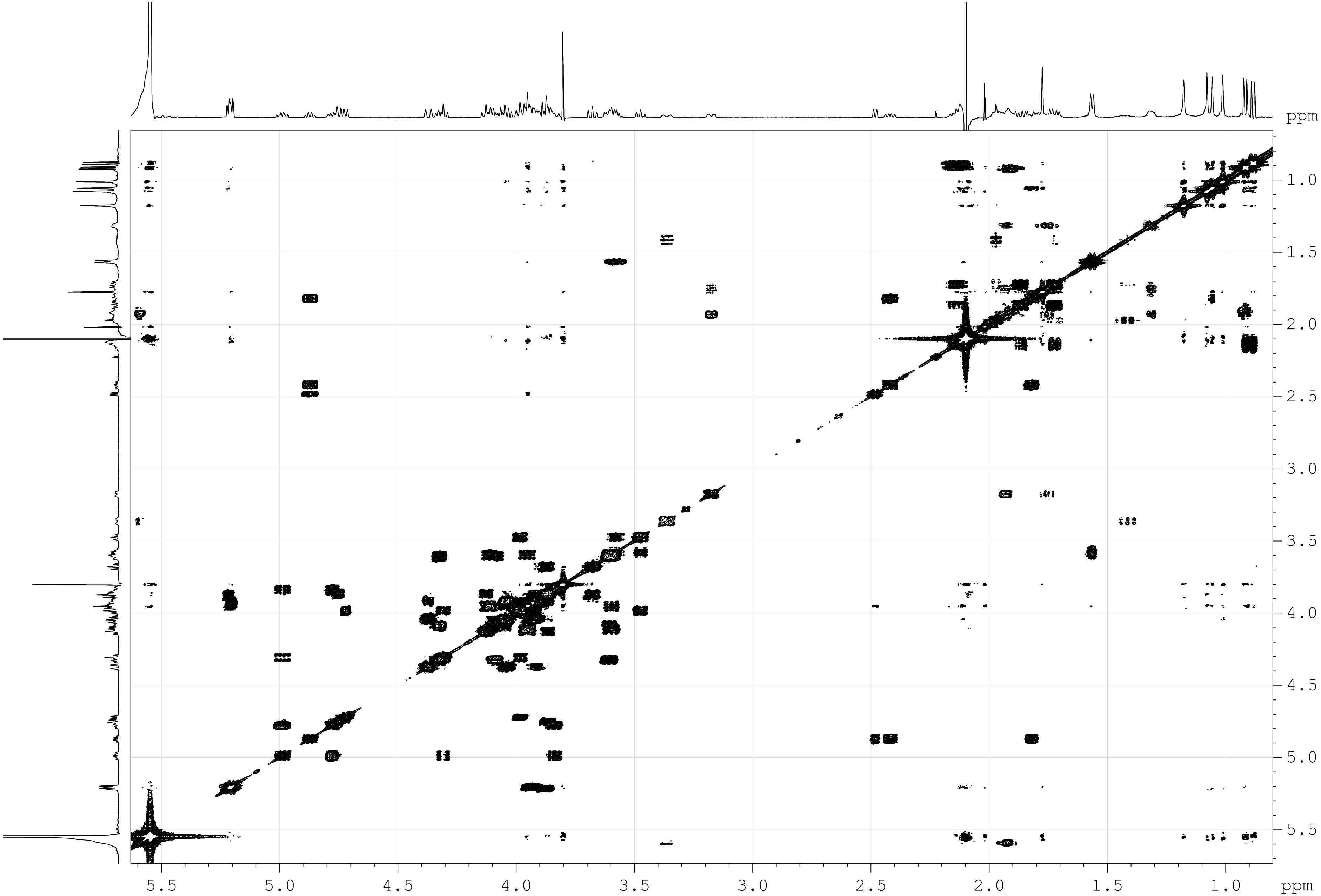

Figure S52. The COSY (500.12 MHz) spectrum of djakonovioside B_4 (**7**) in C_5D_5N/D_2O (4/1)

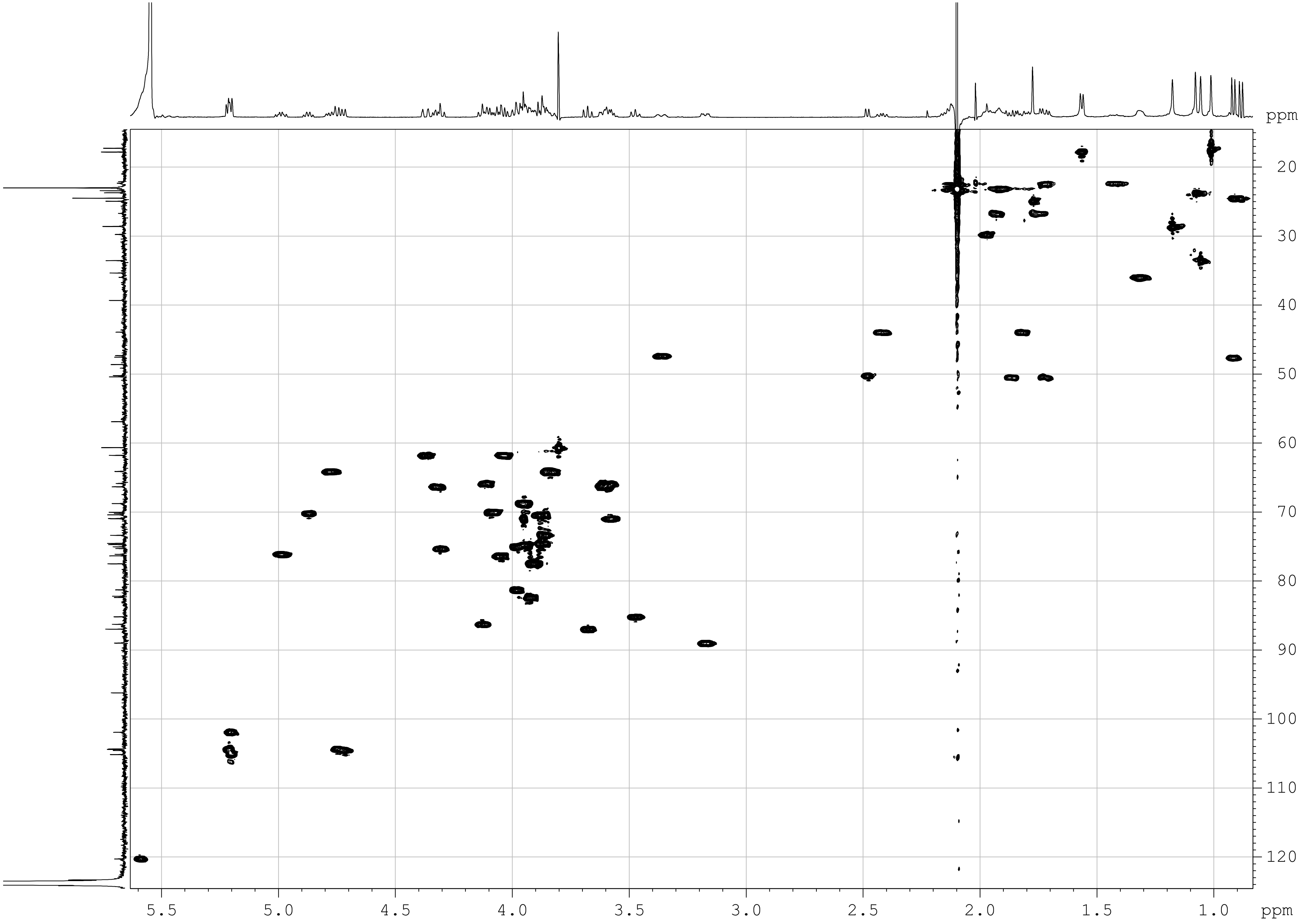

Figure S53. The HSQC (500.12 MHz) spectrum of djakonovioside B4 (**7**) in C_5D_5N/D_2O (4/1)

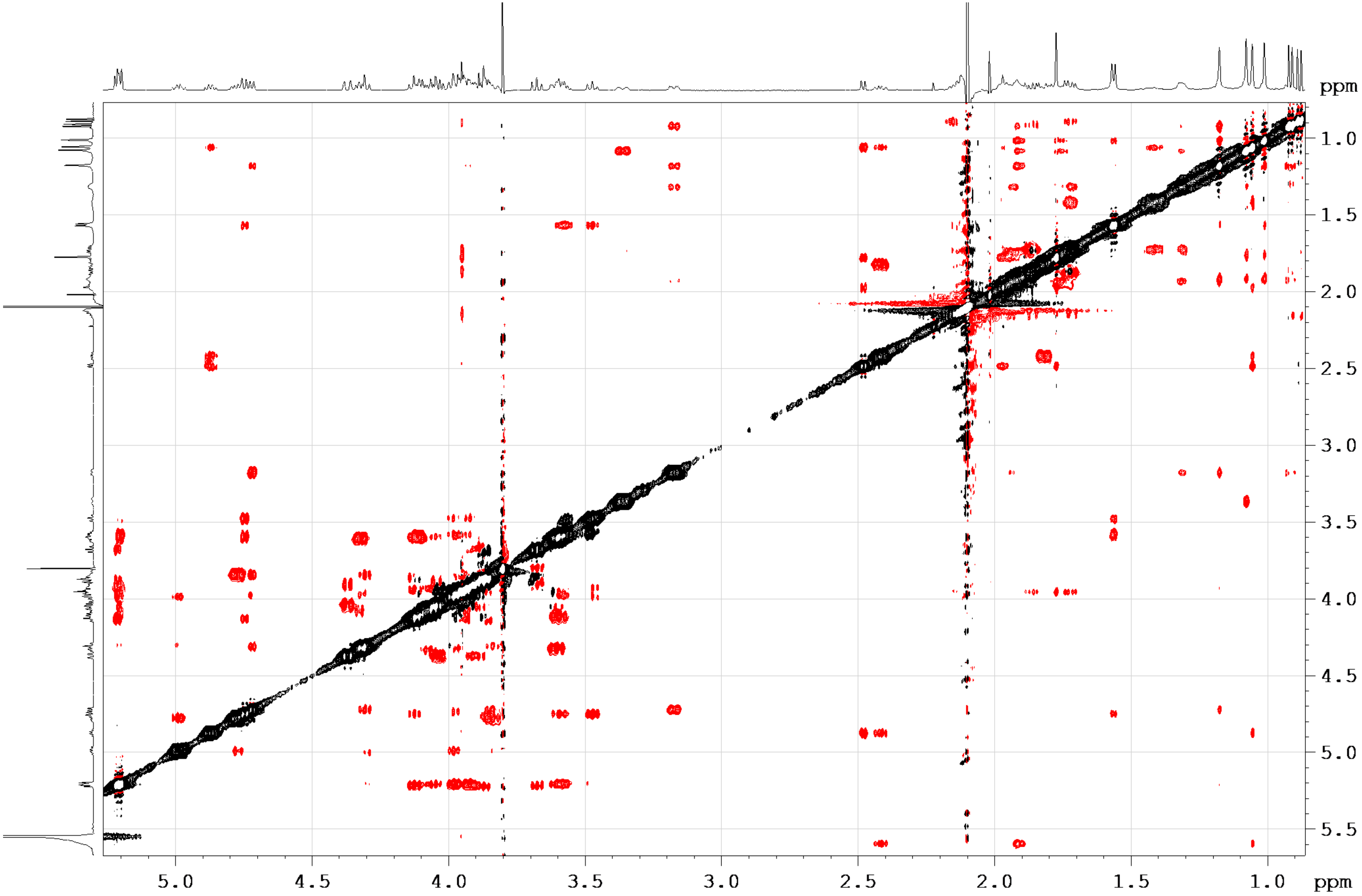

Figure S54. The ROESY (500.12 MHz) spectrum of djakonovioside B4 (7) in C_5D_5N/D_2O (4/1)

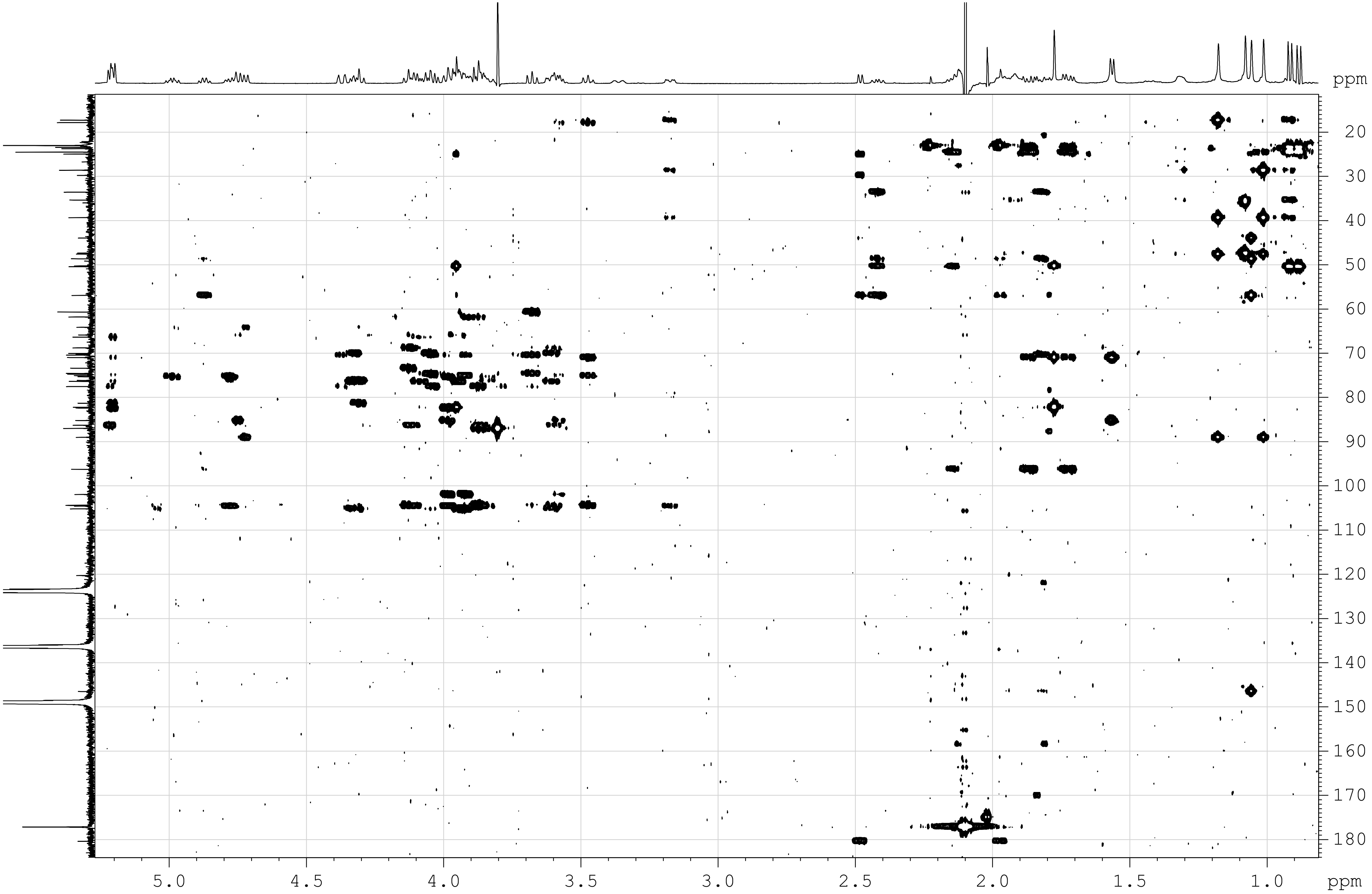

Figure S55. The HMBC (500.12 MHz) spectrum of djakonovioside B$_4$ (**7**) in C_5D_5N/D_2O (4/1)

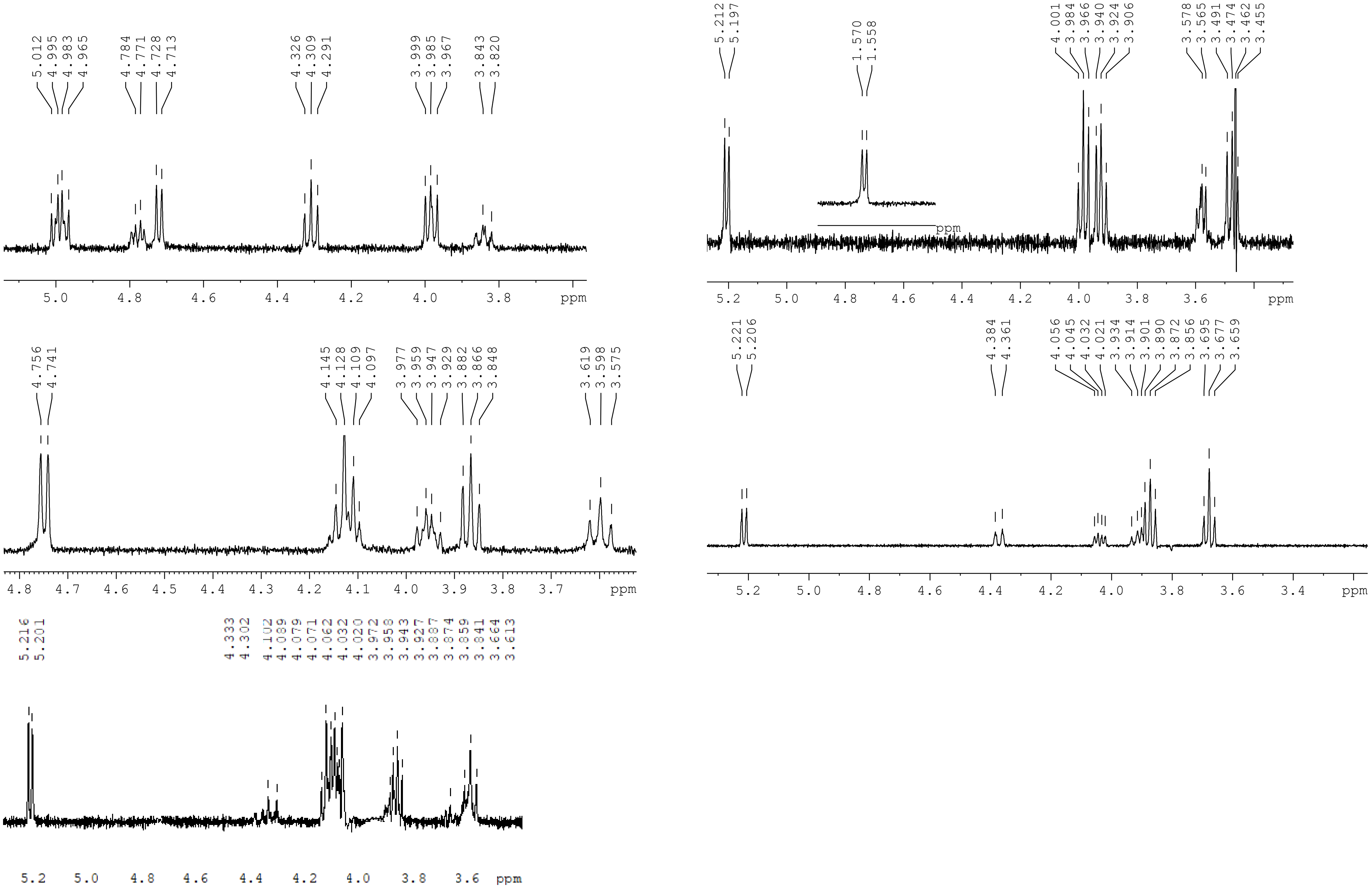

Figure S56. 1D TOCSY (500.12 MHz) spectra of Xyl1, Qui2, Xyl3, MeGlc4, Xyl5 of djakonovioside B$_4$ (**7**) in C_5D_5N/D_2O (4/1)

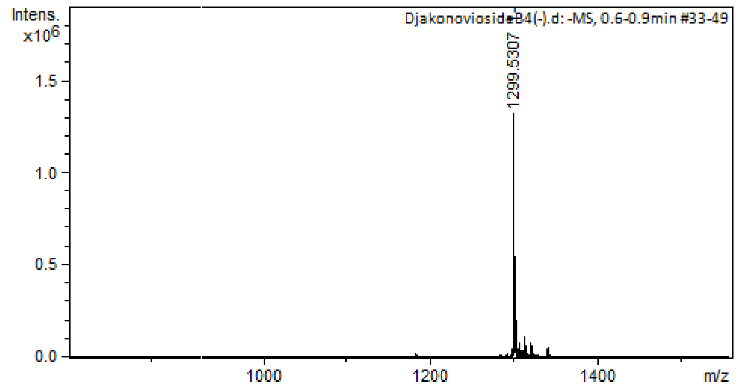

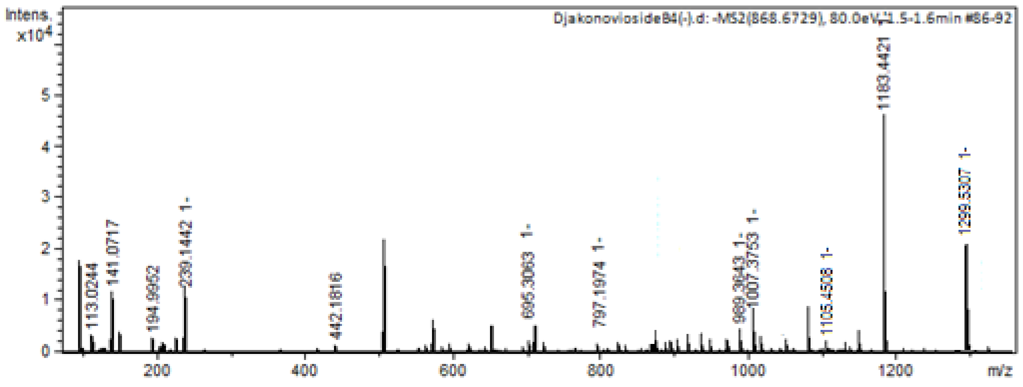

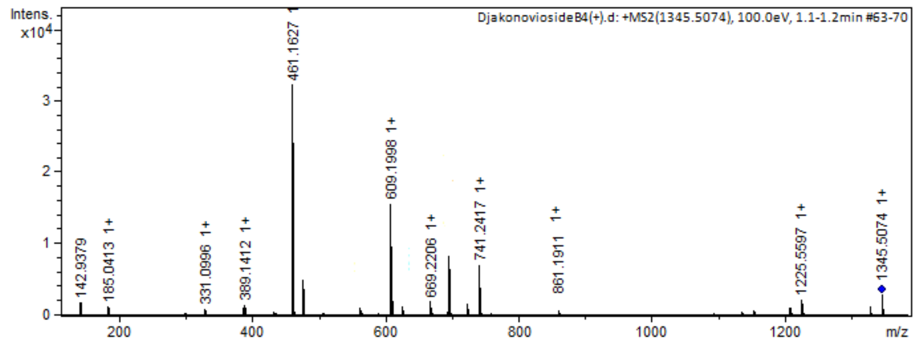

Figure S57. HR-ESI-MS and ESI-MS/MS spectra of djakonovioside B4 (**7**)

Table S6. ^{13}C and ^{1}H NMR chemical shifts and HMBC and ROESY correlations of carbohydrate moiety of **djakonovioside B$_4$**. [a] Recorded at 176.04 MHz in C_5D_5N. [b] Recorded at 500.13 MHz in C_5D_5N/D_2O. Multiplicity by 1D TOCSY.

Atom	δC mult.[a]	δH mult. (*J* in Hz)[b,c,d]	HMBC	ROESY
Xyl1 (1→C-3)				
1	104.6 CH	4.73 d (7.5)	C: 3; C: 5 Xyl1	H-3; H-3, 5 Xyl1
2	**81.3** CH	3.98 t (8.7)	C: 1 Qui2; C: 1, 3 Xyl1	H-1 Qui2
3	75.3 CH	4.31 t (8,7)	C: 2, 4 Xyl1	H-1, 5 Xyl1
4	*76.1* CH	4.99 ddd (5.0; 8.7; 14.3)	C: 3 Xyl1	
5	64.1 CH_2	4.78 dd (5.0; 11.8)	C: 1, 3 Xyl1	
		3.84 dd (8.7; 11.8)		H-1, 3 Xyl1
Qui2 (1→2Xyl1)				
1	104.3 CH	5.21 d (7.8)	C: 2 Xyl1	H-2 Xyl1; H-3, 5 Qui2
2	**82.4** CH	3.91 t (8.6)	C: 3 Qui2; C: 1 Xyl5	
3	75.0 CH	3.98 t (8.6)	C: 2, 4 Qui2	H-1, 5 Qui2
4	**85.2** CH	3.47 t (8.6)	C: 3, 5, 6 Qui2; C: 1	H-1 Xyl3
5	70.9 CH	3.58 dd (6.5; 8.6)	C: 4 Qui2	H-1 Qui2
6	17.8 CH_3	1.56 d (6.5)	C: 4, 5 Qui2	
Xyl3 (1→4Qui2)				
1	104.4 CH	4.75 d (7.5)	C: 4 Qui2	H-4 Qui2; H-3, 5 Xyl3
2	73.4 CH	3.87 t (8.6)	C: 1 Xyl3	
3	**86.3** CH	4.13 t (8.6)	C: 2, 4 Xyl3	H-1 MeGlc4; H-1 Xyl3
4	68.8 CH	3.95 m		
5	65.9 CH_2	4.12 dd (5.2; 11.8)	C: 1, 3 Xyl3	
		3.60 t (10.7)	C: 1, 4 Xyl3	H-1, 3 Xyl3
MeGlc4 (1→3Xyl3)				
1	105.2 CH	5.22 d (8.0)	C: 3 Xyl3	H-3 Xyl3; H-3, 5
2	74.5 CH	3.87 t (8.7)		
3	87.0 CH	3.68 t (8.7)	C: 2, 4 MeGlc4; OMe	H-1, 5 MeGlc4
4	70.4 CH	3.87 t (8.7)	C: 3, 5, 6 MeGlc4	
5	77.5 CH	3.91 m	C: 6 MeGlc4	H-1 MeGlc4
6	61.8 CH_2	4.38 brd (11.8)		
		4.04 dd (5.6; 11.8)	C: 5 MeGlc4	
OMe	60.7 CH_3	3.81 s	C: 3 MeGlc4	
Xyl5 (1→2Qui2)				
1	101.9 CH	5.21 d (8.0)	C: 2 Qui2; X: 5Xyl5	H-2 Qui2
2	74.7 CH	3.96 t (8.0)	C: 3 Xyl5	
3	76.3 CH	4.06 t (8.0)	C: 2, 4 Xyl5	H-1, 5 Xyl5
4	70.1 CH	4.09 m	C: 3 Xyl5	
5	66.3 CH_2	4.33 d (4.9; 12.0)	C: 3, 4 Xyl5	
		3.61 t (10.8)	C: 3, 4 Xyl5	H-1 Xyl5

[c] Bold = interglycosidic positions, [d] Italic – sulfate positions.

Table S7. ^{13}C and ^{1}H NMR chemical shifts, HMBC and ROESY correlations of aglycone moiety of djakonovioside B4 (**7**).

Position	δ_C mult.[a]	δ_H mult. (*J* in Hz) [b]	HMBC	ROESY
1	36.0 CH_2	1.32 m	C: 31	H-3, H-11, H-19
2	26.7 CH_2	1.93 m		
		1.75 m		H-19, H-30
3	89.0 CH	3.17 dd (3.6; 11.6)	C: 4, 30, 31, C: 1 Xyl1	H-1, H-5, H-31, H1-Xyl1
4	39.3 C			
5	47.5 CH	0.91 m	C: 1, 4, 30	H-3, H-31
6	23.1 CH_2	1.91 m		H-19, H-30, H-31
7	120.3 CH	5.59 m		H-15, H-32
8	146.5 C			
9	47.3 CH	3.36 brd (14.6)		H-19
10	35.3 C			
11	22.3 CH_2	1.72 m		
		1.41 m		H-32
12	29.8 CH_2	1.97 m	C: 11, 13, 14, 18	H-17
13	56.9 C			
14	48.6 C			
15	43.9 CH_2	2.42 dd (6.9; 12.6)	C: 13, 14, 17, 32	H-7, H-32
		1.82 m	C: 14, 16, 32	
16	70.2 CH	4.87 dd (6.6; 13.3)	C: 13, 14, 23	H-32
17	50.2 CH	2.48 d (6.9)	C: 12, 13, 18, 21	H-12, H-21, H-32
18	180.3 C			
19	23.7 CH_3	1.08 s	C: 1, 9, 10	H-1, H-2, H-9
20	82.2 C			
21	24.9 CH_3	1.78 s	C: 17, 20, 22	H-17, H-22
22	70.9 CH	3.95 s	C: 17, 20, 21	H-21, H-24, H-27
23	96.2 C			
24	50.4 CH_2	1.86 m	C: 22, 23, 25, 26, 27	H-22
		1.72 dd (5.3; 13.6)	C: 22, 23, 25, 26, 27	H-22, H-27
25	23.4 CH	2.12 m	C: 23, 24, 26, 27	
26	24.5 CH_3	0.91 d (6.5)	C: 24, 25, 27	H-25
27	24.5 CH_3	0.87 d (6.5)	C: 24, 25, 26	H-25
30	17.3 CH_3	1.01 s	C: 3, 4, 5, 31	H-2, H-6, H-31
31	28.6 CH_3	1.18 s	C: 3, 4, 5, 30	H-3, H-5, H-6, H-30, H-1 Xyl1
32	33.5 CH_3	1.06 s	C: 8, 13, 14, 15	H-7, H-11, H-12, H-15, H-16, H-

[a] Recorded at 125.67 MHz in C_5D_5N/D_2O (4/1). [b] Recorded at 500.12 MHz in C_5D_5N/D_2O (4/1).

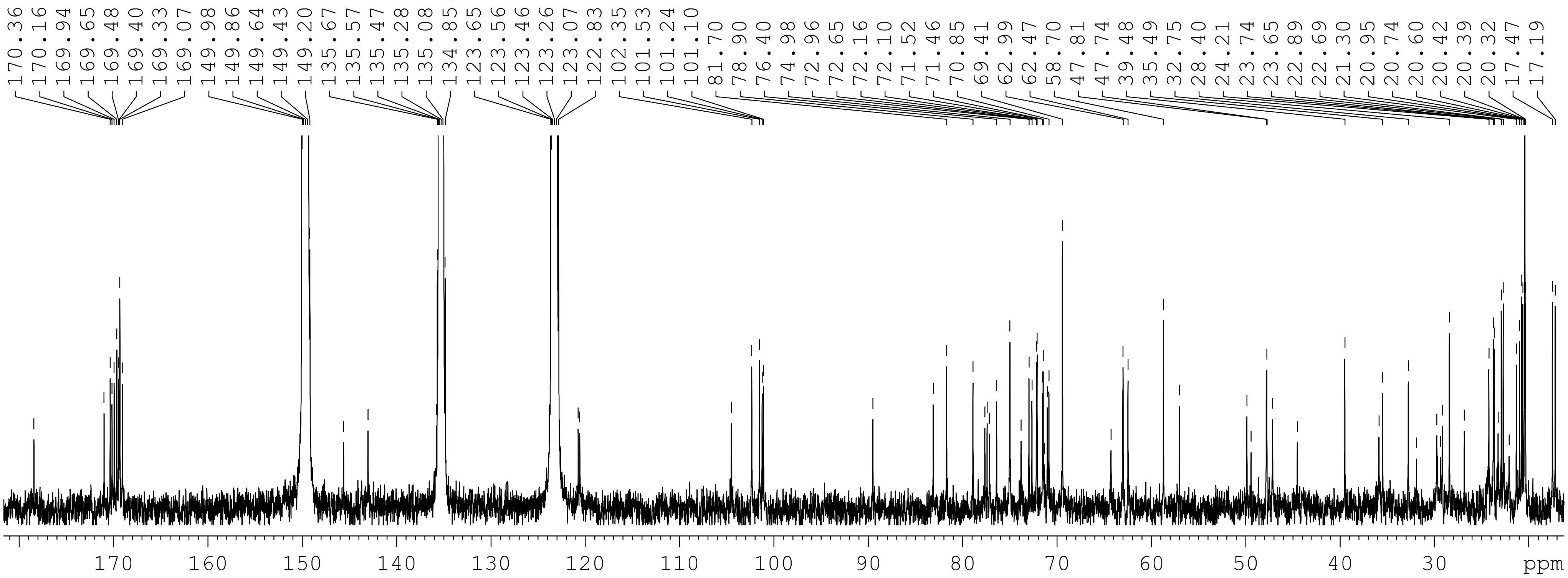

Figure S58. The ^{13}C NMR (125.67 MHz) spectrum of acetylated derivative **7a** in C_5D_5N

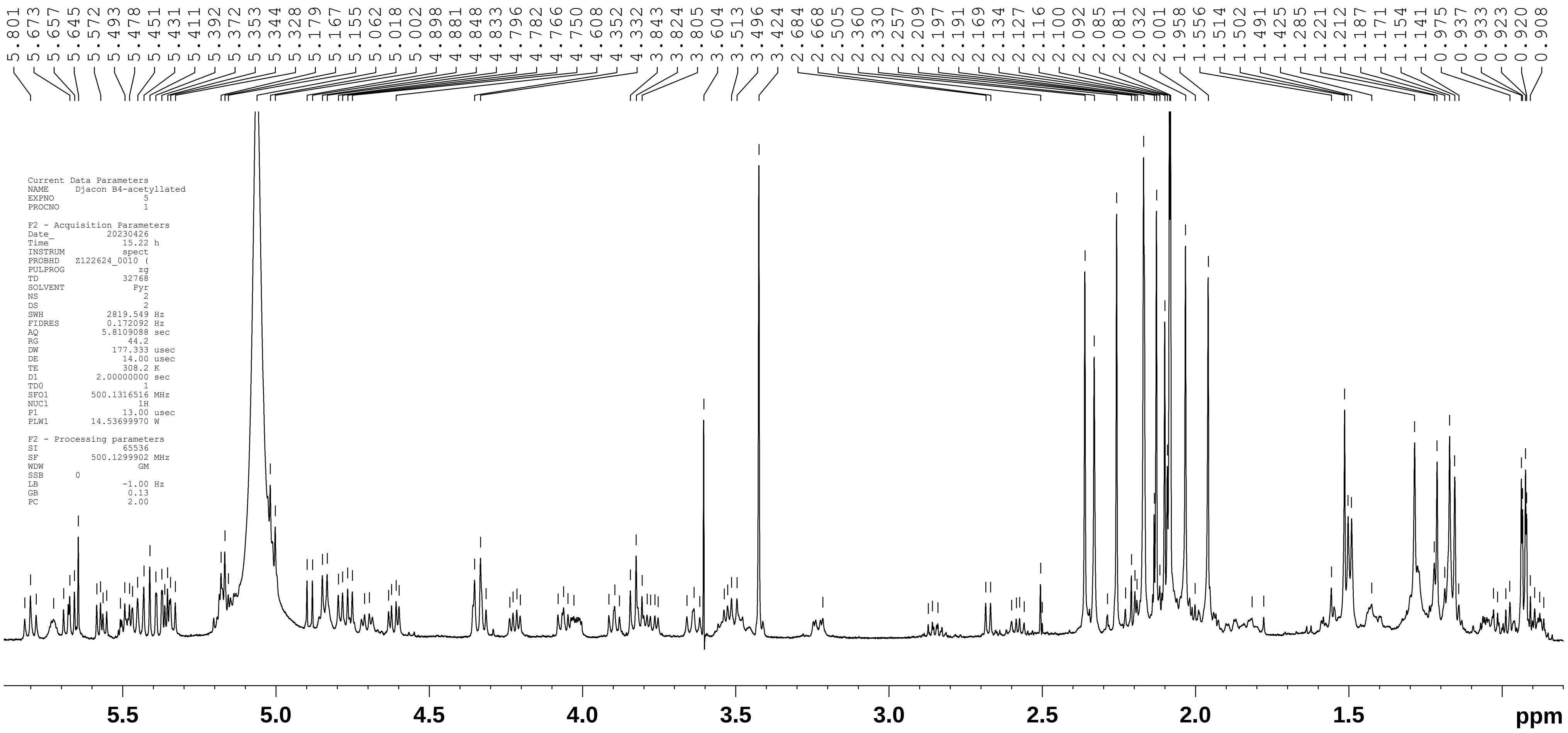

Figure S59. The ^{1}H NMR (500.12 MHz) spectrum of acetylated derivative **7a** in C_5D_5N

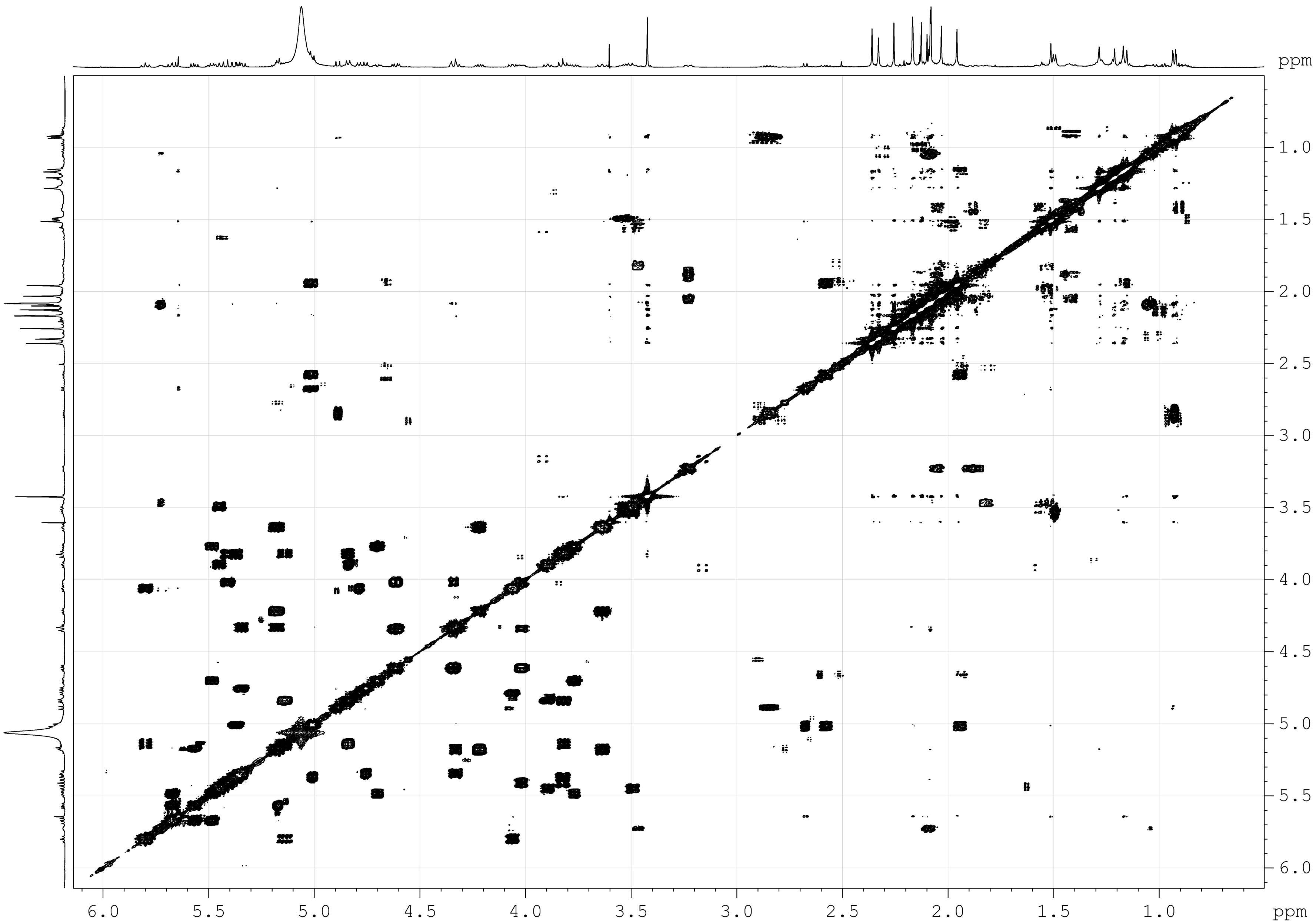

Figure S60. The COSY (500.12 MHz) spectrum of acetylated derivative **7a** in C_5D_5N

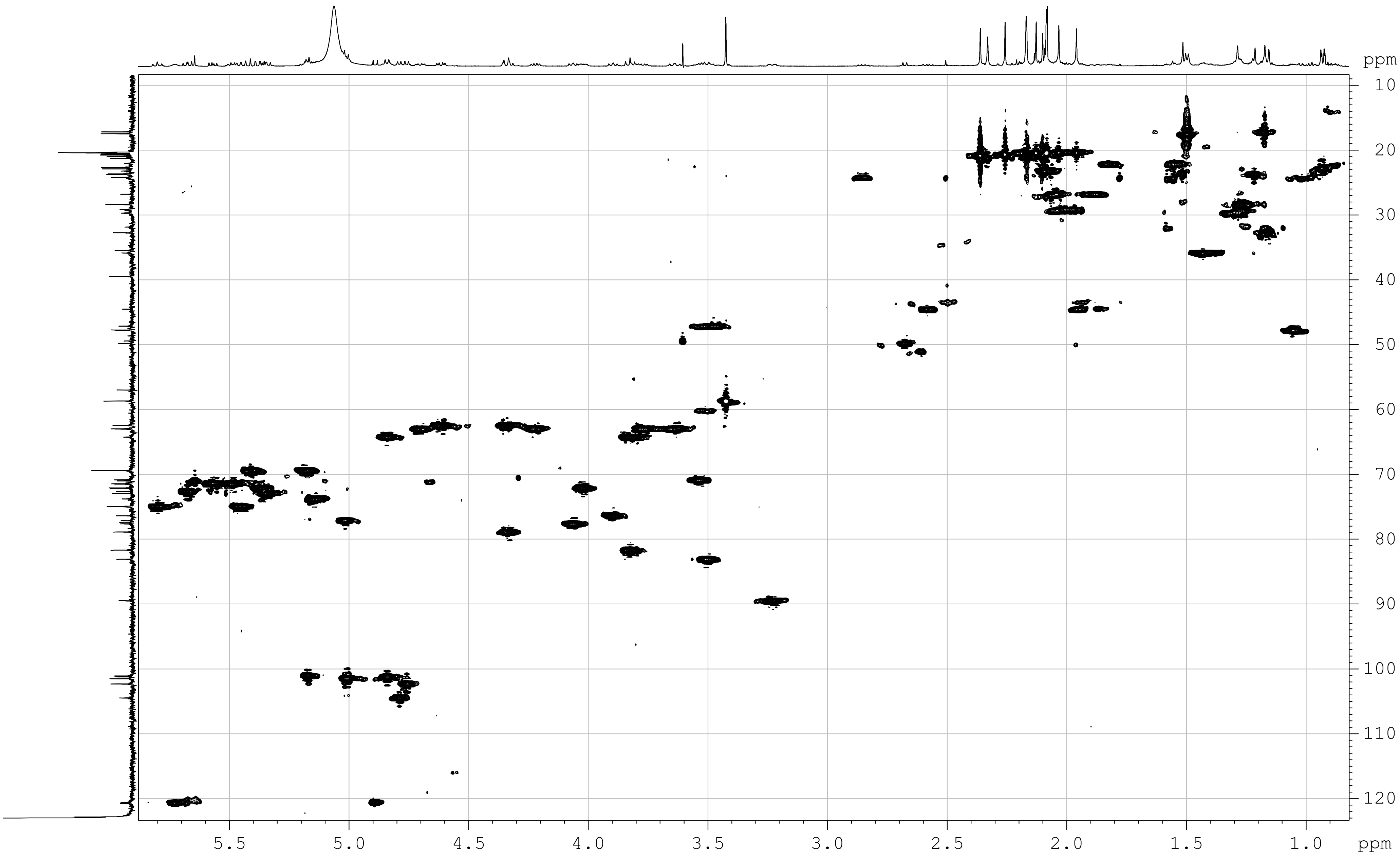

Figure S61. The HSQC (500.12 MHz) spectrum of acetylated derivative **7a** in C_5D_5N

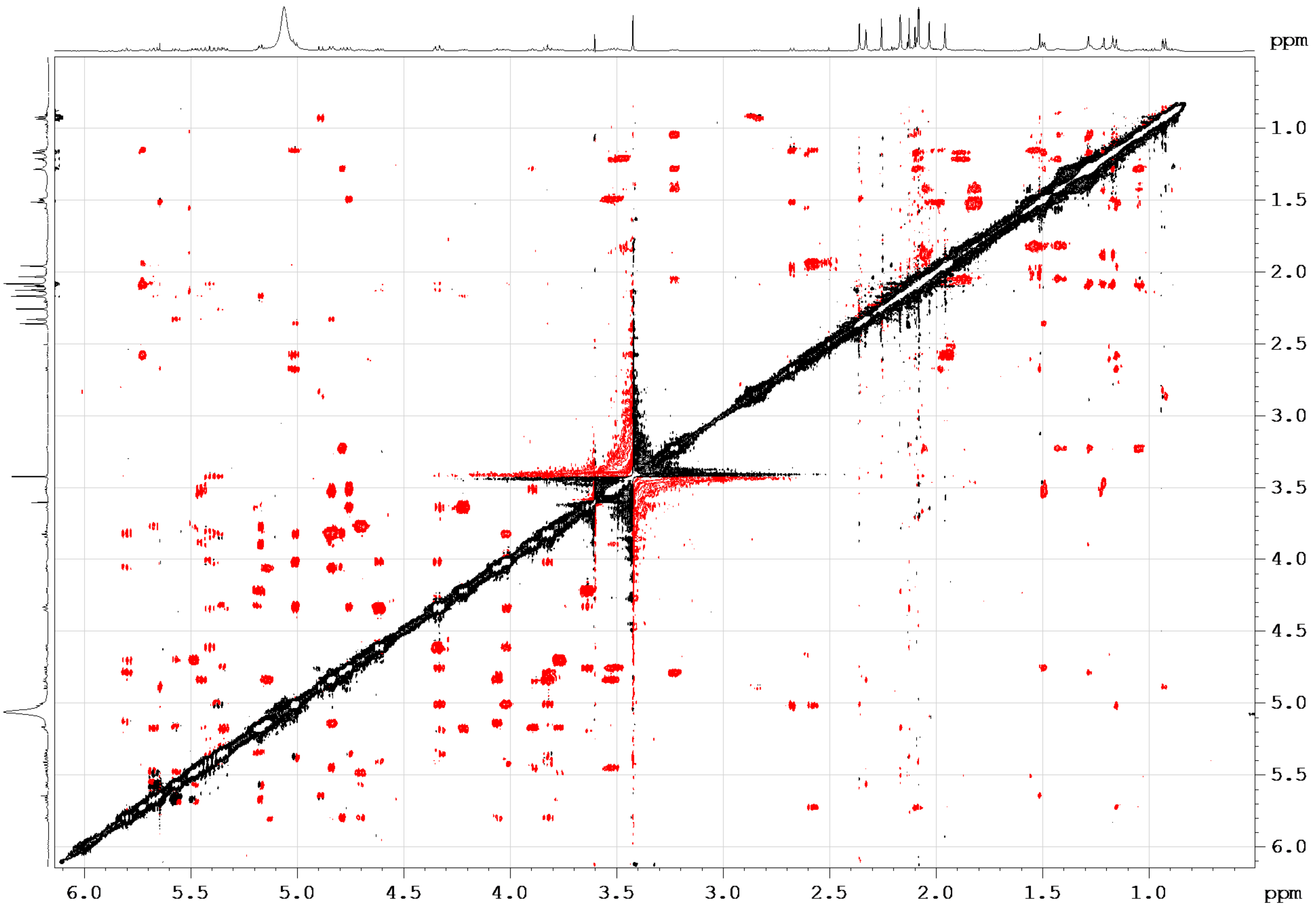

Figure S62. The ROESY (500.12 MHz) spectrum of acetylated derivative **7a** in C_5D_5N

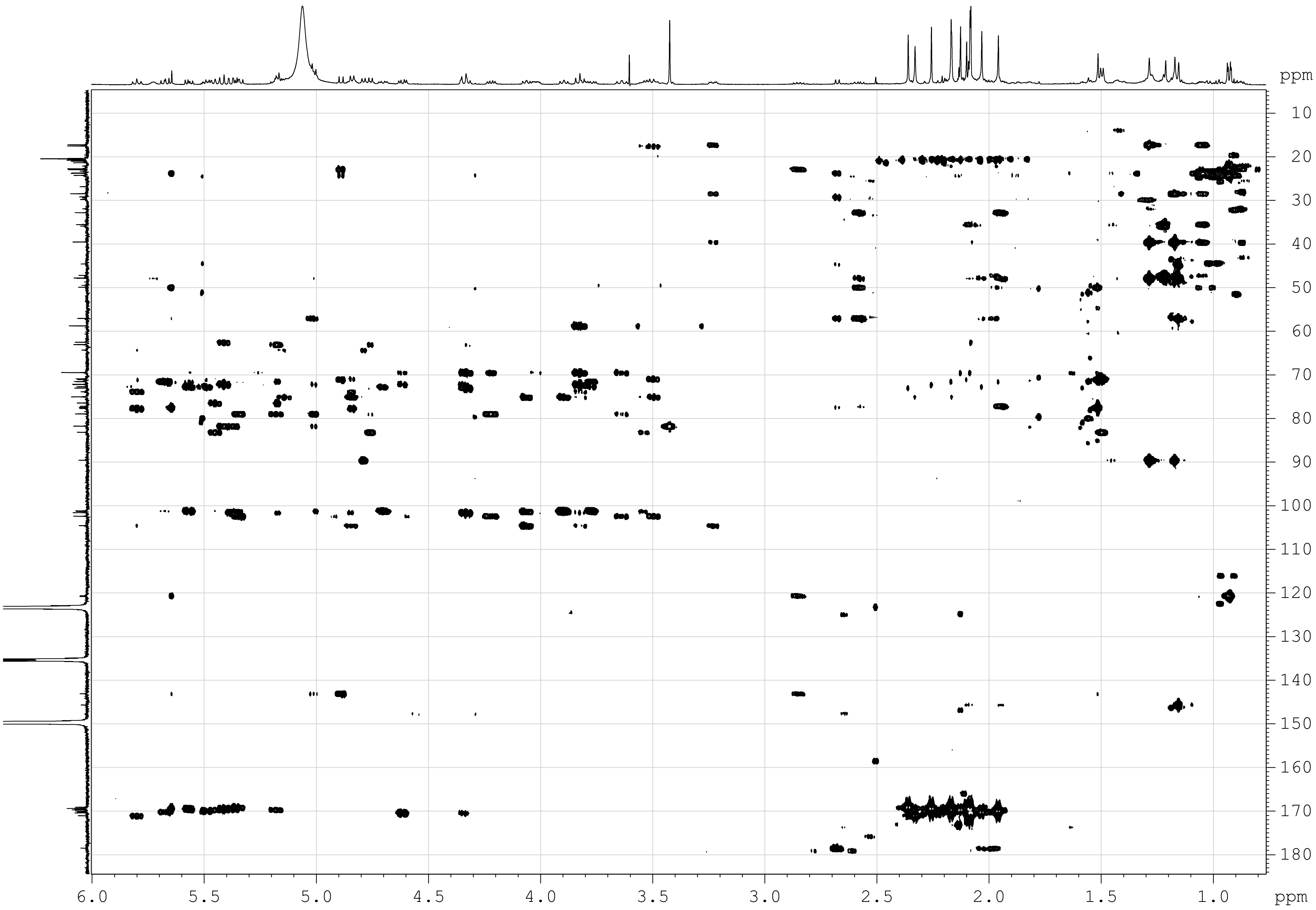

Figure S63. The HMBC (500.12 MHz) spectrum of acetylated derivative **7a** in C_5D_5N

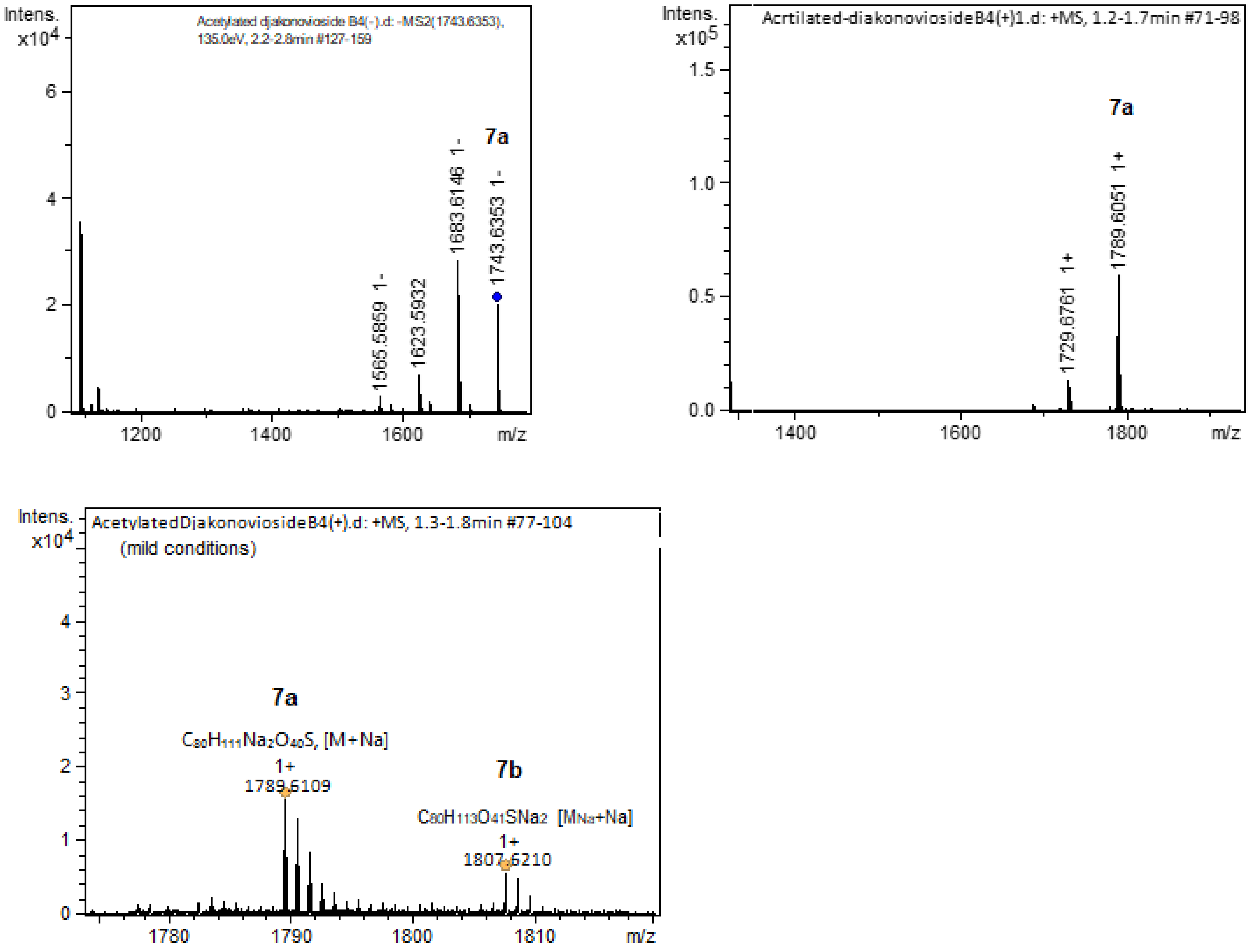

Figure S64. (–) and (+) HR-ESI-MS spectra of acetylated derivatives of djakonovioside B_4 (**7**)

Table S8. ^{13}C and ^{1}H NMR chemical shifts, HMBC and ROESY correlations of aglycone moiety of acetylated derivative **7a**.

Position	δ_C mult.[a]	δ_H mult. (*J* in Hz) [b]	HMBC	ROESY
1	35.9 CH_2	1.41 m		H-3, H-11, H-19
2	26.8 CH_2	2.04 m		
		1.88 m		
3	89.5 CH	3.23 dd (3.8; 11.4)	C: 1 Xyl1	H-1, H-5, H-31, H1-Xyl1
4	39.5 C			
5	47.7 CH	1.05 m		
6	23.2 CH_2	2.09 m		
7	120.7 CH	5.73 m		H-15, H-32
8	145.6 C			
9	47.1 CH	3.49 m		H-19
10	35.5 C			
11	22.1 CH_2	1.83 m		
		1.55 m		
12	29.1 CH_2	2.00 m	C: 18	
13	57.0 C			
14	47.8 C			
15	44.5 CH_2	2.58 dd (7.3; 12.6)	C: 13, 14, 16, 17, 32	
		1.94 m		
16	77.2 CH	5.02 brd (8.0)	C: 13, 14, 23	H-32
17	49.9 CH	2.68 d (7.8)	C: 12, 13, 15, 18, 20, 21	H-21, H-32
18	178.4 C			
19	23.6 CH_3	1.21 s	C: 1, 9, 10	H-9
20	77.4 C			
21	23.7 CH_3	1.51 s	C: 17, 20, 22	H-17, H-22
22	71.0 CH	5.65 s	C: 17, 20, 21, 24, OAc	H-17, H-21, H-24
23	143.2 C			
24	120.6 CH	4.89 d (9.0)	C: 22, 23, 25, 26, 27	
25	24.2 CH	2.85 m	C: 23, 24, 26, 27	H-26 (27)
26	22.9 CH_3	0.93 d (6.7)	C: 24, 25, 27	H-24
27	22.7 CH_3	0.93 d (6.7)	C: 24, 25, 26	H-24
30	17.2 CH_3	1.17 s	C: 3, 4, 5, 31	H-2, H-6, H-31
31	28.4 CH_3	1.28 s	C: 3, 4, 5, 30	H-3, H-5, H-6, H-30, H-1 Xyl1
32	32.7 CH_3	1.15 s	C: 8, 13, 14, 15	H-7, H-12, H-15, H-16, H-17

[a] Recorded at 125.67 MHz in C_5D_5N. [b] Recorded at 500.12 MHz in C_5D_5N.

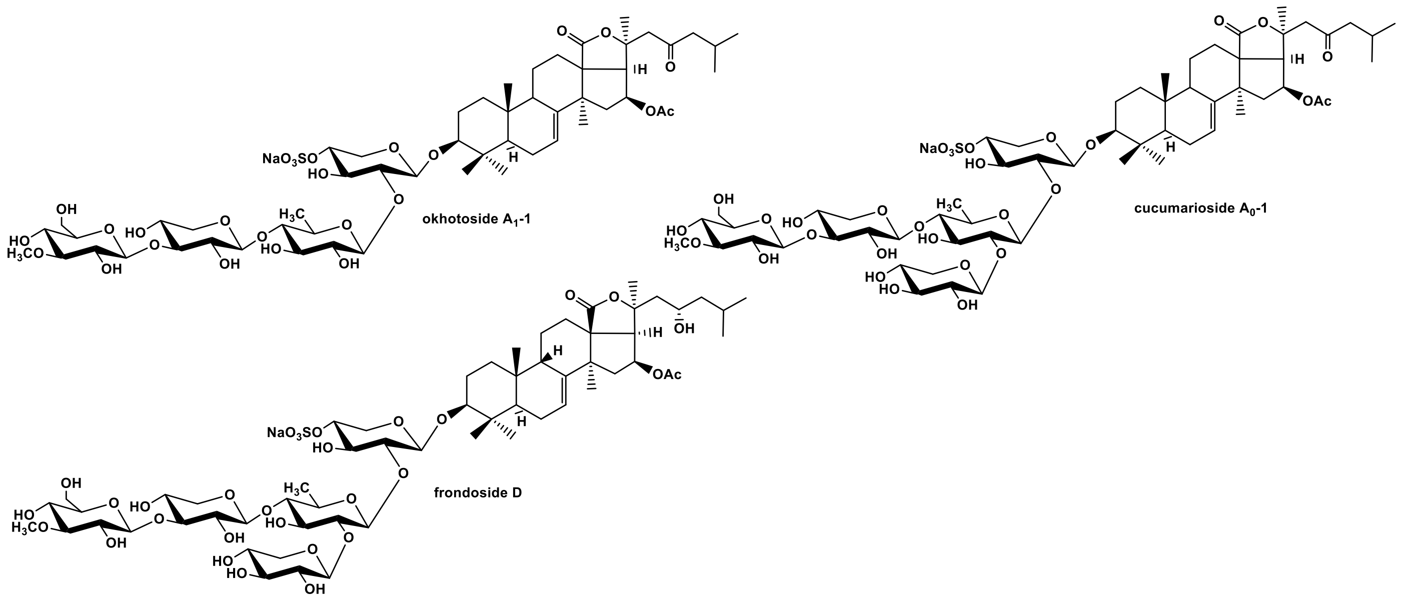

Figure S65. Formulae of known glycosides isolated from *C. djakonovi*
